# A subcomplex comprising TRAPPC11, TRAPPC12, TRAPPC13 and the fungal TRAPPC2L homolog, Tca17, directs TRAPPIII to autophagy

**DOI:** 10.1242/jcs.264822

**Published:** 2026-08-27

**Authors:** Mario Pinar, Vivian de los Ríos, Silvia Rodríguez-Pires, Eduardo A. Espeso, Miguel A. Peñalva

**Affiliations:** ^1^ Department of Molecular and Cellular Biosciences, CSIC Centro de Investigaciones Biológicas Margarita Salas, Ramiro de Maeztu, 9, Madrid 28040, Spain; ^2^ Proteomics and Genomics facility, CSIC Centro de Investigaciones Biológicas Margarita Salas, Ramiro de Maeztu, 9, Madrid 28040, Spain

**Keywords:** Autophagy, TRAPP complex, Intracellular traffic, *Aspergillus nidulans*

## Abstract

Transport protein particle complexes (TRAPPs) are master regulators of membrane trafficking. TRAPPs are targeted to different locales by pathway-specific subunits decorating a core hetero-heptamer to build TRAPPII (Golgi exit) and TRAPPIII (autophagosomes and ER-Golgi trafficking). Metazoan and *Arabidopsis* TRAPPIII have three components, TRAPPC11, TRAPPC12 and TRAPPC13 (hereafter denoted TRAPPC11/12/13), that are absent from budding yeast. We studied TRAPPC11/12/13 in the related ascomycete *Aspergillus nidulans*, where TRAPPC11 and TRAPPC12 localize to pre-autophagosomes and their ablation impairs autophagy. We found that two stable subcomplexes containing Tca17, the homolog of metazoan TRAPPC2L, coexist – one includes the TRAPPII-specific subunits Trs120, Trs130 and Trs65 whereas the other contains the TRAPPIII-specific subunits TRAPPC11/12/13. Both are recruited to core TRAPP by Tca17, which therefore plays a crucial role by determining the physiological role of TRAPP. TRAPPIII also exists in two versions, TRAPPIIIa and TRAPPIIIb, both of which contain Trs85, the homolog of metazoan TRAPPC8, but with only TRAPPIIIb containing TRAPPC11/12/13, which target TRAPPIII to autophagy. This study might help characterize potentially pathogenic mutations affecting human TRAPPC11/12/13, facilitating assessment of their functional consequences in a genetically amenable ascomycete.

## Teaser

**Summary:** The TRAPPIII complex regulates both the autophagy and the secretory pathways. A subcomplex comprising TRAPPC11, TRAPPC12, TRAPPC13 and the fungal TRAPPC2L homolog, Tca17, targets *Aspergillus* TRAPPIII to autophagy.

## INTRODUCTION

Rab GTPases (RABs) regulate organelle identity by recruiting cytosolic proteins to membranes, thereby determining the characteristic composition of each subcellular compartment (Behnia and Munro, 2005). RABs are molecular timers alternating between active GTP-loaded, and inactive GDP-loaded, conformations, of which only the former exposes and inserts the prenylated C-terminus into a membrane and binds its cognate effectors efficiently (Pfeffer and Aivazian, 2004; Lamber et al., 2019). The high affinity that GDP has for the active site implies that the exchange of GTP for GDP requires the action of guanine nucleotide exchange factors (GEFs), which facilitate the release of GDP, making the nucleotide pocket accessible to GTP (Barr, 2013; Lamber et al., 2019). It is broadly accepted that the membrane specificity of RABs is determined by the cognate GEF. Therefore, understanding the molecular physiology of these GEFs is a fundamental issue in cell biology.

**Fig. 1.**
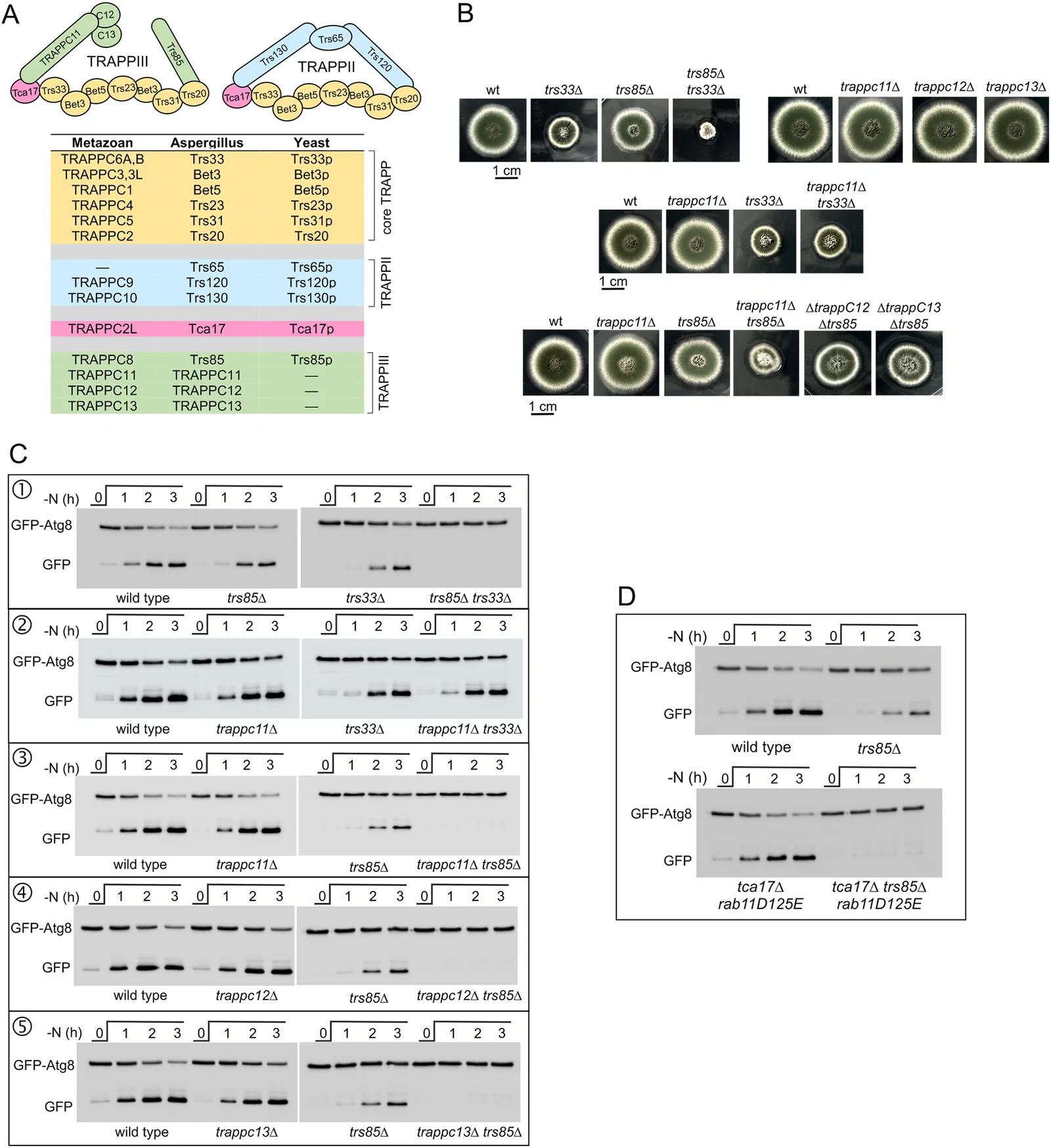
**Loss of Trs85 alone is insufficient to block autophagy.** (A) Nomenclature of the proteins composing the fungal and metazoan TRAPP complexes and schematic representation of *A. nidulans* TRAPPII and TRAPPIII. (B) Growth tests of the indicated *A. nidulans* strains cultured on complete medium for 3 days at 37°C. (C) GFP–Atg8 processing assays in cells of the indicated genotypes that had been shifted from N-sufficient medium to N-starved medium to induce autophagy. In these assays, GFP–Atg8 present in the membrane of autophagosomes is delivered to the lumen of the vacuoles and proteolytically processed until the GFP moiety accumulates due to its resistance to vacuolar proteases. Full-length GFP-Atg8 and ‘free’ GFP were detected by anti-GFP antibody western blotting. (D) GFP–Atg8 processing assays demonstrating that autophagy is abolished in a *tca17*Δ *trs85*Δ double mutant. Blots shown are representative of one biological repeat, with confirmation provided by other blots in this figure.

Transport protein particle complexes (TRAPPs) mediate nucleotide exchange on the GTPases RAB1 and RAB11 (Jones et al., 2000; Cai et al., 2008; Zou et al., 2012; Pinar et al., 2015; Riedel et al., 2018; Thomas et al., 2018; Galindo et al., 2021; Glick, 2021; Joiner et al., 2021; Mi et al., 2022). Several TRAPP complexes have been described in the literature, all consisting of a core hetero-heptamer that is targeted to RAB1 or RAB11 depending on the presence of additional accessory TRAPP components. Intensive research has shaped the currently accepted paradigm according to which there are only two TRAPP complexes, denoted TRAPPII and TRAPPIII; TRAPPII is targeted to RAB11 by three or four subunits that modify the core, whereas TRAPPIII is targeted to RAB1 by Trs85 (TRAPPC8 in metazoans) (Sacher et al., 2000; Morozova et al., 2006; Lynch-Day et al., 2010; Tan et al., 2013; Pinar et al., 2015; Riedel et al., 2018; Thomas et al., 2018, 2019; Jenkins et al., 2020; Bagde and Fromme, 2022; Mi et al., 2022) (the composition of TRAPPs and the nomenclature of the different subunits is described in Fig. 1A).

RAB1, the substrate of TRAPPIII, participates in two physiologically antithetic pathways, exocytosis, specifically during transport between the ER and early Golgi, where RAB1 is recruited to COPII vesicles, and autophagy, where RAB1 is recruited to pre-autophagosomes (Lynch-Day et al., 2010; Tan et al., 2013; Zhao et al., 2017; Thomas et al., 2018; Joiner et al., 2021). As yet outstanding is the problem of how TRAPPIII can differentially recruit and activate RAB1 to each of these locales. Although a substantial amount of data on disease-causing amino acid substitutions in the human TRAPP components has accumulated (Sacher et al., 2019), the only structure available to date for a metazoan TRAPPIII is that from *Drosophila melanogaster* (Galindo et al., 2021). Studies with *Saccharomyces cerevisiae* have made key contributions to our understanding of the structural organization and physiology of the TRAPP complexes, but this unicellular eukaryote lacks homologues of TRAPPC11, TRAPPC12 and TRAPPC13 (hereafter denoted TRAPPC11/12/13), three of the 11 subunits of metazoan and plant TRAPPIII (Kalde et al., 2019; Glick, 2021; Galindo and Munro, 2023), thereby limiting further insight into mammalian TRAPPs exploiting this unicellular ascomycete. The genome of *S. cerevisiae* (12 Mbp and 5900 coding genes) was shaped by reductive evolution resulting in extensive gene loss (Shen et al., 2018), a process that has not occurred in *Aspergillus nidulans* (30 Mbp and 12,000 genes), another genetically amenable ascomycete.

Using *A. nidulans*, we previously determined how TRAPPII is directed towards RAB11 and characterized the composition of TRAPPs (Pinar et al., 2019; Pinar and Peñalva, 2020). We showed that unlike *S. cerevisiae*, *A. nidulans* has homologues of TRAPPC11/12/13 and that TRAPPIII is composed of the core hetero-heptamer plus Trs85, like in the budding yeast (Pinar et al., 2019). Here, we revisit this conclusion by demonstrating the existence of two different TRAPPIII complexes, TRAPPIIIa (formerly called TRAPPIII), which does not contain TRAPPC11/12/13, and TRAPPIIIb, which does contain these proteins. We also establish that TRAPPC11, the subunit that links this subcomplex to the core, targets TRAPPIIIb to autophagy.

## RESULTS

### Blocking autophagy requires ablation of both Trs85 and TRAPPC11/12/13

We reported previously that *A. nidulans* TRAPPIII, like its *S. cerevisiae* counterpart, consists of the banana-shaped core heterohexamer of essential subunits flanked at each end by a non-essential subunit, Trs33 (TRAPPC6 in metazoans) and Trs85, respectively (Fig. 1A) (Tan et al., 2013; Pinar et al., 2019). Filamentous fungi grow by apical extension, a mechanism that is exquisitely dependent on exocytosis (Oakley and Peñalva, 2025). Defects in exocytosis can be easily scored with growth tests monitoring colony size. A paradoxical observation was that *trs85*Δ impairs growth only slightly (Pinar and Peñalva, 2020) (Fig. 1B, top left), despite the fact that TRAPPIII is known to activate RAB1 during ER-to-Golgi transport (Thomas et al., 2018). The second TRAPPIII non-essential subunit, Trs33 is also a component of TRAPPII (the GEF for RAB11 at the trans-Golgi network), and therefore plays a dual exocytic role, which is reflected in the noticeable growth inhibition resulting from its ablation (Fig. 1B, top left). We reasoned that sensitizing cells with a *trs33*Δ mutation could uncover the role of Trs85 in exocytosis. This was indeed the case. A double *trs33*Δ *trs85*Δ mutation showed a strong synthetic negative interaction (Fig. 1B, top left).

In addition to its exocytic role, Trs85 is necessary and sufficient to target TRAPPIII to pre-autophagosomes (PASs), the Atg8-containing structures that give rise to autophagosomes, as *S. cerevisiae trs85*Δ precludes N-starvation-induced autophagy (Nazarko et al., 2005). However, mutant *Aspergillus trs85*Δ hyphae still retain significant Atg8 processing activity, a proxy of autophagy (Pinar et al., 2013) (Fig. 1C-1), establishing that Trs85-independent mechanisms activating RAB1 in PASs exist. Notably, ablation of Trs33 impaired Atg8 processing to a similar extent to what was seen with *trs85*Δ (Fig. 1C-1), suggesting that in *Aspergillus* Trs33 cooperates with Trs85 in autophagy. Indeed, Atg8 processing was completely blocked in a *trs85*Δ *trs33*Δ double mutant strain (Fig. 1C-1). Thus, *A. nidulans* Trs33 plays a direct or indirect role in autophagy.

**Fig. 2.**
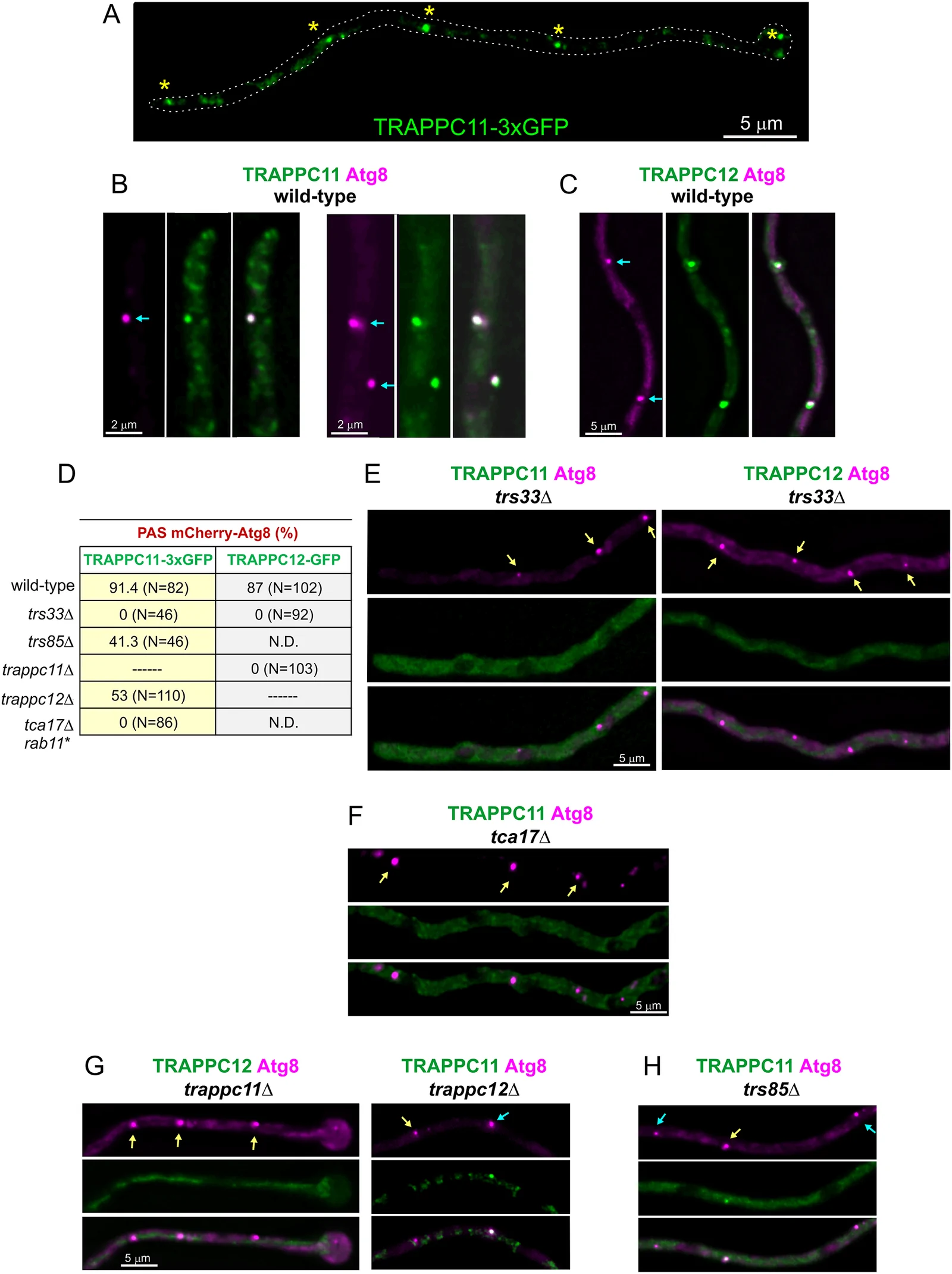
**Endogenously tagged TRAPPC11 and TRAPPC12 localize to pre-autophagosomes.** (A) localization of TRAPPC11 to pre-autophagosomes (asterisks) across the length of a hypha (dotted outline). (B,E–H) PASs are labeled with mCherry–Atg8. PASs containing TRAPPC11 or TRAPPC12 are indicated with cyan arrows, whereas those that do not contain TRAPPC11 or TRAPPC12 are indicated with yellow arrows. (B) TRAPPC11 colocalizes with PASs. Examples of PASs located near and away from the tip are shown. (C) TRAPPC12 colocalizes with Atg8 in PASs. (D) Quantitative data for localization experiments. Data for TRAPPC11 correspond to a total of 32 wild-type hyphae, 16 *trs33*Δ hyphae, 18 *trs85*Δ hyphae, 37 *trapp12*Δ hyphae and 30 *tca17*Δ hyphae (viability of *tca17*Δ cells was rescued with a hyperactive *rab11* allele, indicated with a superscripted asterisk). Data for TRAPPC12 correspond to a total of 35 wild-type hyphae, 25 *trs33*Δ hyphae and 26 *trapp11*Δ hyphae; N.D., not done. (E) Neither TRAPPC11 nor TRAPPC12 localize to the PAS in cells ablated for Trs33. (F) TRAPPC11 does not localize to PASs in the absence of Tca17. (G) TRAPPC12 does not localize to PASs in the absence of TRAPPC11*,* whereas localization of TRAPPC11 is not prevented by the absence of TRAPPC12. (H) Ablation of Trs85 reduces the proportion of Atg8 PASs containing TRAPPC11.

**Fig. 3.**
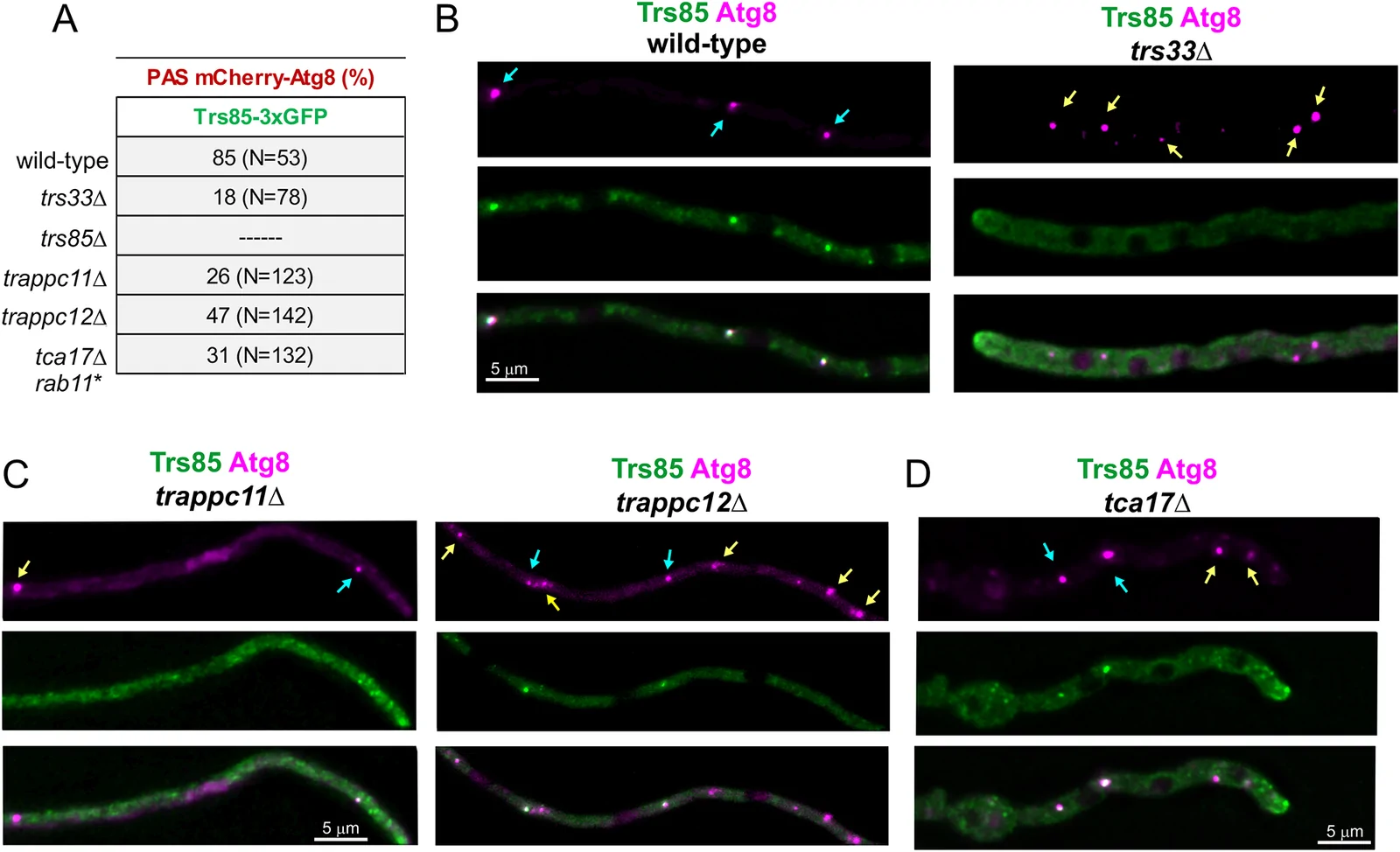
**The localization of Trs85 to PASs is impaired in cells lacking Trs33 or the Tca17 and TRAPPC11/12/13 subcomplex.** (A) Quantification of Trs85–3×GFP localization to the mCherry–Atg8 PASs in different mutant backgrounds. Data correspond to a total of 12 wild-type hyphae, 15 *trs33*Δ hyphae, 34 *trappc11*Δ hyphae, 34 *trapp12*Δ hyphae and 39 *tca17*Δ hyphae. (B) Trs85 localization to PASs is markedly reduced in cells lacking Trs33. (C) Trs85 TRP85 localization to PASs is impaired in cells lacking TRAPPC11, TRAPPC12 or Tca17. PASs containing TRAPPC11 or TRAPPC12 are indicated with cyan arrows, whereas those that do not contain TRAPPC11 or TRAPPC12 are indicated with yellow arrows.

**Fig. 4.**
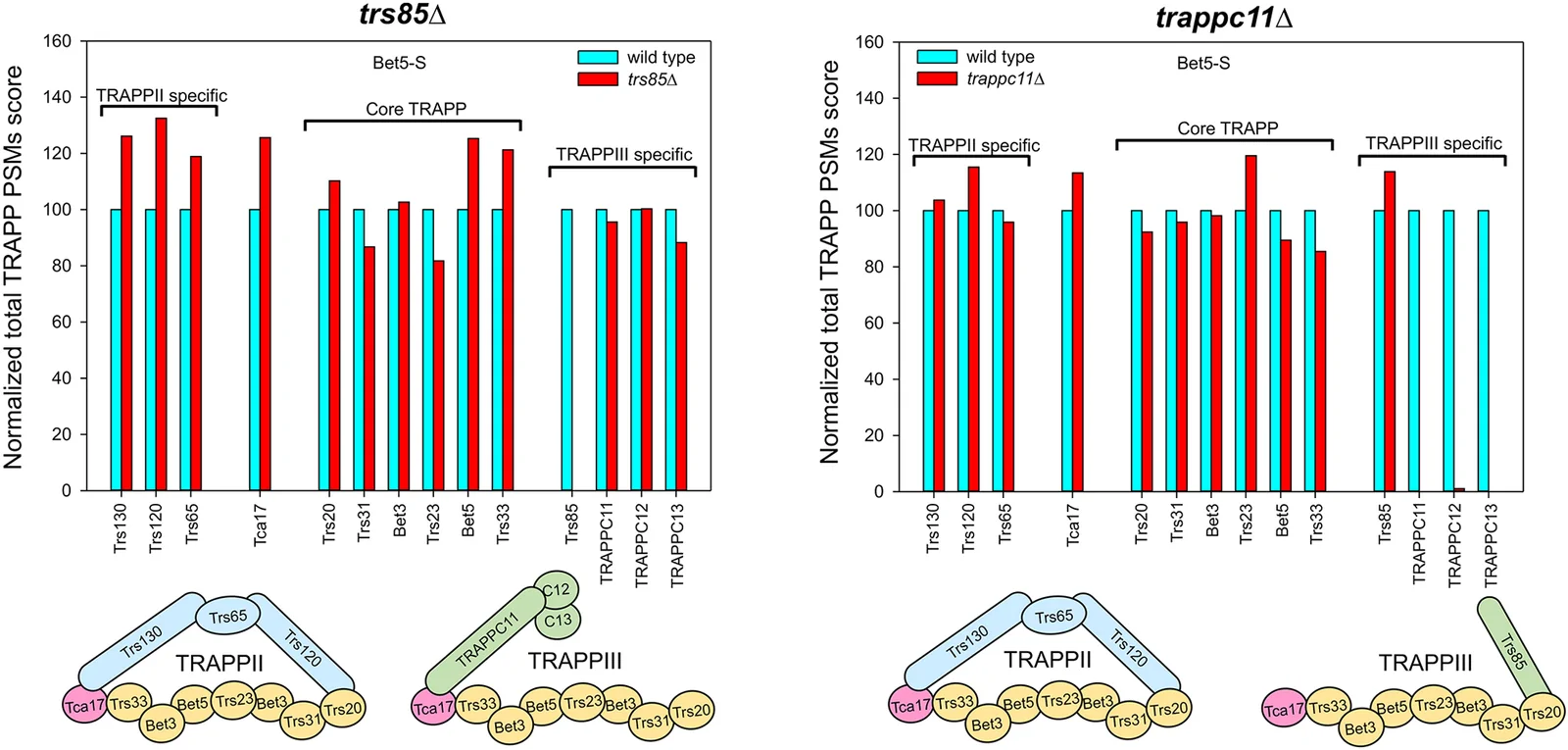
**The absence of either Trs85 or TRAPPC11 does not disrupt TRAPPIII organization.** The composition of TRAPPs, purified from *trs85*Δ and *trappc11*Δ cells as assessed by Bet5-S affinity chromatography, was analyzed by MS/MS. The actual PSM counts for each protein in the wild-type condition was set as 100%. Data represent the results of one biological replicate for each experiment. In both cases, the relevant mutant and wild-type strains carrying the S-tagged bait were cultured and lysed, subjected to S-tag purification of TRAPPCs and analysed by MS/MS in parallel throughout the entire procedure, as detailed in the Materials and Methods.

**Fig. 5.**
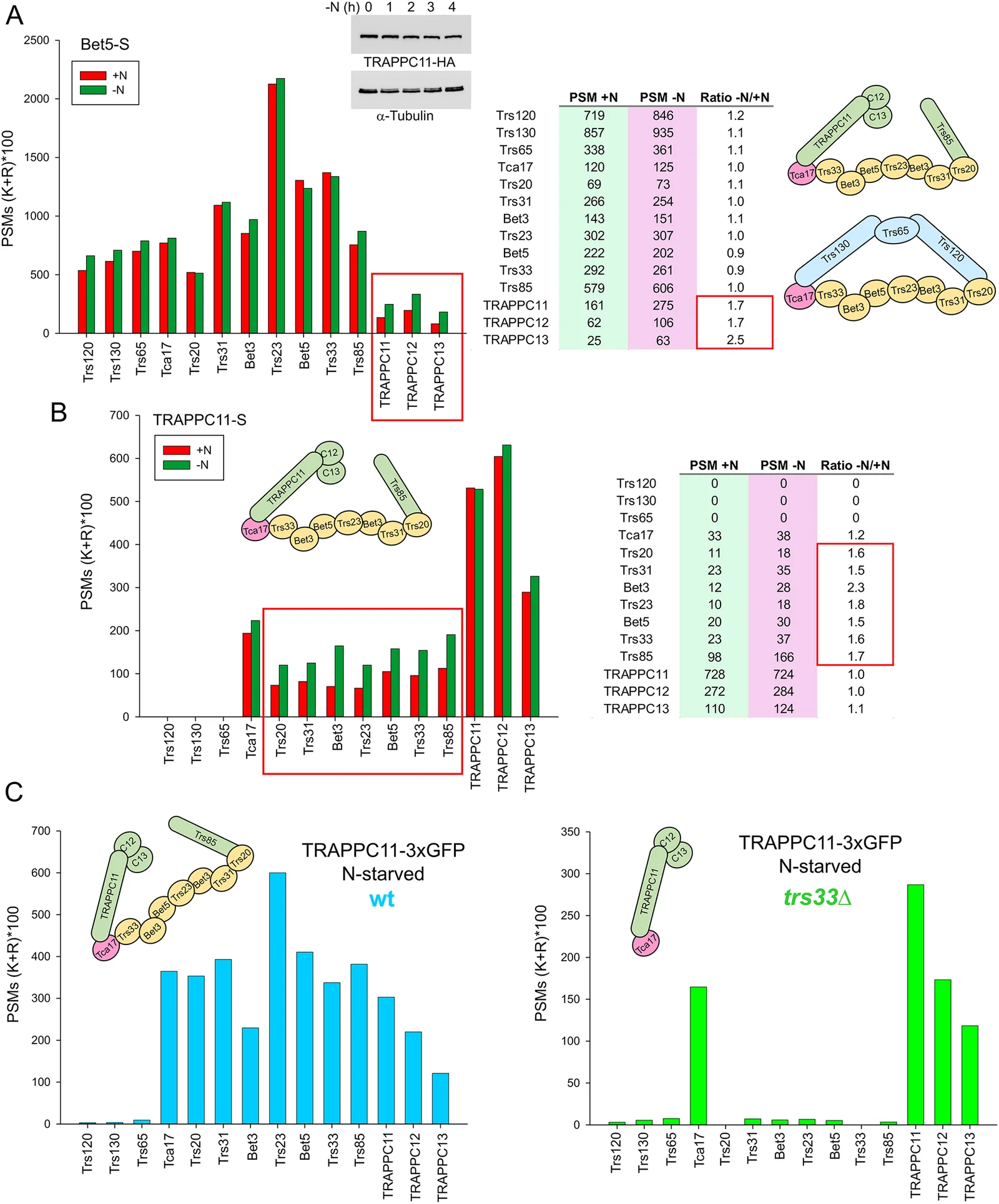
**The amount of core TRAPP associated with a subcomplex containing TRAPPC11/12/13 and Tca17 increases upon shifting cells to N-starved conditions.** (A) Abundance of TRAPP subunits in Bet5-S purified material. Bet5 is a pan-TRAPP subunit. Bars indicate spectral counts normalized to the number of Lys and Arg residues for each protein (which is used as an approximation to the number of tryptic peptides potentially detectable by MS/MS for each protein) multiplied by 100. Displayed on the right table are the ratios of actual spectral counts obtained under N-sufficient (N+) and N-starving (N-) conditions. The western blot in the middle shows that the amount of HA_3_-tagged TRAPPC11 does not increase after starving cells for nitrogen (anti-α-tubulin was used as a loading control). (B) The amount of TRAPP core subunits associated with TRAPPC11 increases upon shifting cells to nitrogen-starved conditions. Note that TRAPPC11 does not associate at all with TRAPPII-specific subunits Trs120, Trs130, and Trs65, but pulls down Tca17, a subunit reportedly associated with TRAPPII. The table on the right indicates relative values obtained by comparing the ratio between N-sufficient (N+) and N-starving conditions (N-). (C) A stable subcomplex containing Tca17 and TRAPPC11/12/13 can be isolated from cells lacking Trs33. GFP-trapping experiments in which proteins were immunoprecipitated with anti-GFP magnetic agarose beads and subjected to MS/MS analysis. For all three experiments, data represent the results of one biological replicate. For A and B, strains carrying the relevant S-tagged bait were cultured in parallel under N-sufficient and N-starving conditions and subsequently lysed, subjected to S-tag purification of TRAPPCs, and analysed by MS/MS in parallel, as described for Fig. 4. For C, wild-type and *trs33∆* strains expressing the GFP bait were cultured under N-starved conditions, lysed, subjected to immunoprecipitation using anti-GFP beads and analysed by MS/MS in parallel throughout the entire procedure. The TRAPPC11 western blot included in A represents data from one biological replicate, internally controlled by reacting the same blot with anti-α-tubulin antibody to confirm equal loading of the lanes.

Previously, we identified TRAPPC11/12/13 as minor components of TRAPPIII (Pinar et al., 2019). However, mutant *trs33*Δ TRAPPIII does not contain TRAPPC11/12/13 (Pinar et al., 2019), indicating that these three subunits associate with TRAPPIII by way of Trs33 [or Tca17 or both; note that in the absence of Tca17, they do not associate with the complex (Pinar et al., 2019)] (Fig. 1A). TRAPPC11/12/13 do not appear to have any exocytic role, as the corresponding single mutants grow to the same extent as wild type (Fig. 1B, top right), and in double mutants *trs33*Δ and *trappc11*Δ do not show any synthetic negative interaction (Fig. 1B, middle). Therefore, we hypothesized that these three subunits might be responsible for the autophagy deficit displayed by *trs33*Δ cells. We started by testing the effects of ablating TRAPPC11, because TRAPPC12 and TRAPPC13 are recruited to TRAPPIII by way of TRAPPC11 (Galindo et al., 2021), and TRAPPC11, TRAPPC12 and TRAPPC13 form a stable subcomplex (see below). Indeed, GFP–Atg8 processing assays showed that ablating TRAPPC11 alone or in combination with *trs33*Δ impairs N-starvation-induced autophagy to a similar extent to what was seen with *trs33*Δ (Fig. 1C-2). In sharp contrast ablation of TRAPPC11 completely prevented autophagy when combined with ablation of Trs85, mimicking the phenotype of *trs33*Δ *trs85*Δ double mutants (Fig. 1C-3). Similar results were obtained after ablating TRAPPC12 and TRAPPC13, alone or in combination with *trs85*Δ (Fig. 1C-4 and C-5). Therefore, TRAPPC11/12/13 play a physiological role in autophagy that accounts for the role of Trs33, explaining why *trs85*Δ alone does not fully prevent it. In conclusion efficient functioning of autophagy necessitates the concerted action of TRAPPC11/12/13 and Trs85. Notably, whereas single *trappc11*Δ, *trappc12*Δ, and *trappc13*Δ do not show any growth defect by themselves, *trappc11*Δ, but not *trappc12*Δ or *trappc13*Δ, reduces colony growth markedly when combined with *trs85*Δ (Fig. 1B, bottom), suggesting that TRAPPC11 can take over, at least partially, the role of Trs85 in the ER–Golgi interface.

### Efficient recruitment of TRAPPIII to pre-autophagosomes requires both TRAPPC11 and Trs85

Consistent with its proposed role in autophagy, subcellular localization studies of TRAPPC11 endogenously tagged with triplicated GFP showed that it localizes to puncta, a localization typical of *A. nidulans* pre-autophagosomes (PASs) (Fig. 2A; Fig. S1) (Pinar et al., 2013). Indeed, these puncta colocalized with the prototypic PAS marker Atg8 (Fig. 2B). Like TRAPPC11, TRAPPC12 also localizes to PAS structures (Fig. 2C; Fig. S1) (TRAPPC13 was undetectable after endogenous tagging). As expected from the recruitment of TRAPPC11/12/13 to TRAPPIII being mediated by Trs33, TRAPPC11 and TRAPPC12 localization to PASs is dependent on the presence of Trs33 (Fig. 2D). Thus, 91% of TRAPPC11 and 87% of TRAPPC12 puncta contain Atg8 in the wild-type, contrasting with none when the reporters were expressed in the *trs33*Δ background (Fig. 2D,E). TRAPPC11 is not recruited to PASs either in *tca17*Δ hyphae (Fig. 2D,F), in agreement with the fact that TRAPPC11/12/13 do not associate with TRAPPs in the absence of Tca17 (Pinar et al., 2019). Consistent with the recruitment of TRAPPC12 to TRAPPs being mediated by TRAPPC11 (Galindo et al., 2021), TRAPPC12 is not recruited to PASs in *trappc11*Δ hyphae, contrasting with 53% of the PASs containing TRAPPC11 in *trappc12*Δ cells (Fig. 2D,G).

Notably, subcellular localization experiments supported the view that Trs85 and TRAPPC11 cooperate to target TRAPPIII to the PAS: less than half (41%) of Atg8 PASs contained TRAPPC11 in *trs85*Δ hyphae (Fig. 2D,H), only 18% (*N*=78) of the Atg8 PASs displayed detectable levels of Trs85–3×GFP in *trs33*Δ cells compared to 85% (*N*=53) in the wild-type (Fig. 3A,B), and only 26% and 47% of Atg8 PASs contained Trs85–3×GFP in *trappc11*Δ and *trappc12*Δ cells, respectively (Fig. 3A,C). As neither *trs85*Δ nor *trappc11*Δ disorganizes TRAPPIII (Fig. 4), it follows that the efficient recruitment of TRAPPIII to PASs necessitates both Trs85 and TRAPPC11, explaining the partial defect in Atg8 processing documented in the absence of either. Similar to the reduction of Trs85 in the PASs in *trappc11*Δ and *trappc12*Δ cells, in cells ablated for Tca17, which is required for the recruitment of TRAPPC11 and TRAPCC12 to TRAPP, only 31% of Atg8 PASs contained Trs85 (Fig. 3A,D).

### TRAPPC11/12/13 associate with Tca17 in a stable subcomplex

Previous tandem mass spectrometry (MS/MS) analysis of the composition of TRAPPIII pulled down by S-tagged Trs85 had revealed that TRAPPC11/12/13 subunits are sub-stoichiometric (Pinar et al., 2019), but this analysis had been carried out with cells cultured under nitrogen (N)-sufficient conditions. Therefore, we compared the composition of TRAPP complexes isolated from cells cultured under N-sufficient and N-starving conditions. To this end, we purified TRAPPs by S-agarose affinity with S-tagged Bet5 (metazoan TRAPPC1), a pan-TRAPP subunit (Pinar et al., 2019). Notably, the amount of TRAPPC11/12/13 associated with TRAPP was markedly augmented when cells were shifted to N-starving conditions (Fig. 5A), which occurred despite the fact that levels of TRAPPC11 did not increase (Fig. 5A, middle top). As none of the other TRAPP components increased its association with the complex, these data strongly indicate that N-starvation stimulates the incorporation of TRAPPC11/12/13 onto TRAPPIII, contributing to target TRAPPIII to autophagy. MS/MS data in Fig. 5A also indicate that TRAPPC11/12/13 are sub-stoichiometric compared to Trs85, even under N-starving conditions, suggesting that a proportion of TRAPPIII does not contain them.

To buttress the conclusion that N-starvation stimulates the incorporation of TRAPPC11/12/13 onto TRAPPIII, we purified TRAPPs from cells expressing TRAPPC11–S-tag. These experiments reinforced the conclusion that the amount of core TRAPP associated with TRAPPC11 increases when cells are cultured under N-starving conditions (Fig. 5B). MS/MS analysis of the composition of TRAPP complexes additionally provided a key piece of information, namely that Tca17, a component of *Aspergillus* TRAPPII, co-purifies with TRAPPC11, which is in agreement with the *Drosophila* structure establishing that TRAPPC11 is recruited to TRAPPIII by way of TRAPPC2L (the metazoan equivalent to Tca17) (Galindo et al., 2021). Therefore, our data suggest thar two TRAPPIII complexes exist, one containing core TRAPP plus Trs85, denoted TRAPPIIIa and a second containing, in addition, Tca17 and TRAPPC11/12/13, denoted TRAPPIIIb. The finding that a fungal TRAPPC2L is a component of TRAPPIII is notable because Tca17 is absent from *S. cerevisiae* TRAPPIII (Montpetit and Conibear, 2009; Lynch-Day et al., 2010; Tan et al., 2013; Joiner et al., 2021).

In previous work, we identified a stable TRAPPII subcomplex consisting of Trs120, Trs130, Trs65 and Tca17 that can be assembled onto the core TRAPP hetero-heptamer to generate TRAPPII (Pinar et al., 2019). To test whether TRAPPC11/12/13 act in a complex, we purified the proteins associated with TRAPPC11–3×GFP in the wild-type cells and in cells lacking Trs33, which disengages TRAPPC11/12/13 from core TRAPP. Although the complete TRAPPIIIb complex co-purified with TRAPPC11–3×GFP in the wild type, the only proteins co-purifying with TRAPPC11 in the *trs33*Δ mutant were TRAPPC12, TRAPPC13 and Tca17 (Fig. 5C), indicating that these four components form a stable subcomplex. This is a remarkable finding, as it implies that Tca17 determines the fate of the TRAPP core by recruiting the TRAPPII-specific subcomplex or the TRAPPIIIb-specific subcomplex to core TRAPP.

As *Aspergillus* Trs33 recruits TRAPPC11/12/13 by way of Tca17, ablation of the latter should phenocopy the consequences of removing TRAPPC11/12/13 on autophagy by impeding the assembly of the subcomplex that incorporates them into TRAPPIII. Tca17 is essential for viability because its ablation disorganizes TRAPPII, preventing exocytosis (Pinar et al., 2019). However, the role of Tca17 in autophagy can be assessed after bypassing the role of TRAPPII with a constitutively active rab11* allele carrying a D125E mutation affecting the GDP-binding pocket (Pinar et al., 2015; Pinar et al., 2019). rab11* tca17Δ mutants display negligible impairment of autophagy, whereas rab11* tca17Δ *trs85*Δ triple mutants show a complete blockage (Fig. 1D). These data strongly support a dual role of Tca17 in both autophagy and exocytosis by way of its ability to scaffold TRAPPII- and TRAPPIIIb-specific subcomplexes.

Two regions of *D. melanogaster* TRAPPC11 (residues 442–453 and 478–486) within a 15-α-helix solenoid that comprises most of N-terminal half of the protein interact with an α-helix in TRAPPC2L that contains Leu-Asp-Φ motif (where Φ indicates a hydrophobic residue) (Galindo et al., 2021). These features are conserved in humans, *Arabidopsis* and *Aspergillus* (Figs S2, S3). Indeed, AlphaFold predicts that the TRAPPC11 15-helix solenoid and its interaction with the Leu-Asp-Φ motif in Tca17 is conserved (Fig. S3). Density maps of the available TRAPPIII structure did not allow modelling of the C-terminal half of TRAPPC11, nor of TRAPPC12 or TRAPPC13 (Galindo et al., 2021). Thus, we ran AlphaFold predictions with these polypeptides, using the *Aspergillus*, *Drosophila* and human sequences. These three predictions yielded remarkably similar models (Fig. S4A). In all three cases the C-terminal half of TRAPPC11 consisted of three consecutive β-sandwiches, of which the most C-terminal interacted with both TRAPPC12 and TRAPPC13. TRAPPC13 consisted of two β-sandwiches in *Aspergillus* and three in humans and *Drosophila* (Fig. S4B). TRAPPC12 is a 16-helix α-barrel. The two β-sandwiches of *Aspergillus* TRAPPC13 and its equivalents in the human and *Drosophila* predicted protein structures are docked against TRAPPC12. The β-sandwiches are separated by an ∼50-residue loop that passes through the central cavity of the TRAPPC12 α-barrel, contributing to the intimate association between these two subunits (Fig. S4B,C).

### Analysis of TRAPPs under N-sufficient and N-starved conditions support the existence of two TRAPPIII complexes

To investigate the recruitment of TRAPPC11/12/13 to TRAPPIII, we compared the elution profiles of TRAPPC11–HA_3_ and Trs85–HA_3_ after chromatographing cell-free extracts of hyphae co-expressing the respective tagged proteins through a gel filtration column and determining by anti-HA Western blotting the amount of Trs85 and TRAPPC11 in each fraction (Fig. 4A). The predicted molecular mass of TRAPPIIIa (core TRAPP plus Trs85) is 237 kDa, whereas that of TRAPPIIIb (TRAPPIIIa plus TRAPPC11/12/13 and Tca17) is 485 kDa. Because TRAPPIIIa is rod-shaped, it elutes between the 669 kDa and 440 kDa standards (Pinar et al., 2019).

**Fig. 6.**
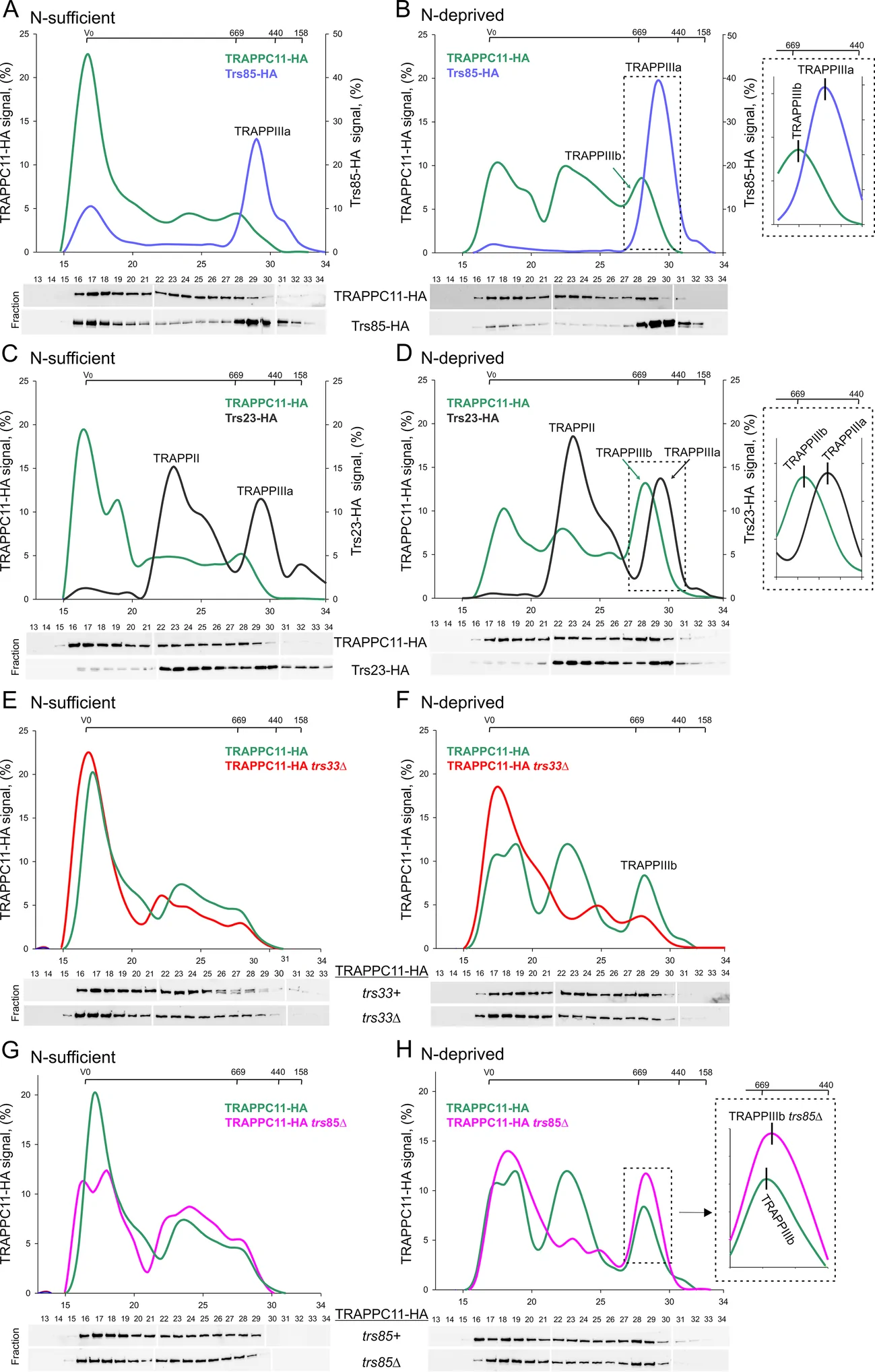
**Two versions of TRAPPIII, containing or not TRAPPC11/12/13 are present in *A. nidulans* extracts.** (A,B) Extracts of cells co-expressing HA_3_-tagged versions of TRAPPC11 and Trs85 cultured under N-sufficient (A) and N-deprived conditions (B) were run through a Superose 6 column and the resulting fractions analyzed by anti-HA western blotting. Samples run on different membranes are separated by white dividers. TRAPPC11, is part of a complex that we denote TRAPPIIIb whose relative abundance increases upon nitrogen starvation and that eludes ahead of TRAPPIII labeled with Trs85, that we denote TRAPPIIIa. (C,D) As in A,B but using a HA_3_-tagged core subunit Trs23 instead of Trs85 to mark the position of all TRAPP complexes. (E,F) The assembly of TRAPPIIIb is dependent on Trs33. (G,H) TRAPPIIIb does not dissemble in absence of Trs85, but its elution is delayed, demonstrating that it contains Trs85. Each panel represents an individual experiment in which two strains were compared in parallel, with confirmation provided by other profiles in this figure.

Under N-sufficient conditions most of TRAPPC11 eluted with the void volume, whereas Trs85 was resolved as a single peak corresponding to TRAPPIIIa (Fig. 6A). In contrast, with N-starved cells, a peak of TRAPPC11 was detected at the region of Trs85, although eluting ahead of it (Fig. 6B). We interpreted that this peak containing both TRAPPC11 and Trs85 (Fig. 6B, blots) corresponds to TRAPPIIIb. To buttress this interpretation, we chromatographed extracts expressing the core subunit Trs23, which marks the positions of all TRAPPs (Pinar et al., 2019) (Fig. 6C). In N-starved cells a large proportion of TRAPPC11 was shifted to this peak eluting ahead of TRAPPIIIa, whose position was here labelled with Trs23–HA_3_ (Fig. 6D).

Two additional pieces of data contributed to the evidence that this forward-shifted TRAPPIII peak corresponds to TRAPPIIIb. One was that it disappeared when Trs33 was ablated, irrespective of the nitrogen condition (Fig. 6E,F). The second was that it is present in cells ablated for Trs85, but its elution is slightly retarded as would be expected from the loss of Trs85 (Fig. 6G,H, see box). In summary, these experiments established that inducing autophagy increased the abundance of TRAPPIII containing TRAPPC11/12/13 (i.e. TRAPPIIIb), in agreement with the above MS/MS analysis. Importantly, they strongly support the existence of two versions of TRAPPIII, including a form (TRAPPIIIb) containing TRAPPC11/12/13 and one (TRAPPIIIa) that does not contain TRAPPC11/12/13. It is tempting to speculate that TRAPPIIIa plays a general role serving both the autophagy and secretory routes whereas TRAPPIIIb is specialized for a role in autophagy.

## DISCUSSION

The mechanistic bases of the autophagy pathway have been largely dissected through studies with *S. cerevisiae*. Subsequent work has demonstrated that this pathway is conserved in metazoans, with a few exceptions. A notable example is that of the oligomeric complex TRAPPIII, which serves as a GEF for the RAB GTPase, RAB1 (Ypt1 in yeast), a master regulator of autophagy and exocytosis. How the same complex discriminates between two such opposing physiological roles (one anabolic, the second catabolic) has started to be understood only recently (Gómez-Sánchez et al., 2025). One important caveat is that *S. cerevisiae* TRAPPIII differs from that in metazoans and plants, which contains three subunits denoted TRAPPC11, TRAPPC12 and TRAPPC13 that are absent in the former (Kalde et al., 2019; Bagde and Fromme, 2023; Galindo and Munro, 2023). This is relevant because mammalian TRAPPC11 has been shown to play a role in autophagy, mediated by its interaction with ATG2B, a lipid transfer protein which facilitates phagophore expansion (Stanga et al., 2019). We have previously shown that the filamentous ascomycete *A. nidulans*, a close relative of *S. cerevisiae* that has not undergone a genomic reduction, is a useful model to study autophagy. For example, in higher cells, autophagosomes are formed in the proximity of ER domains denoted omegasomes, which are absent in the budding yeast, but present in *Aspergillus* (Pinar et al., 2013). We have also reported that RAB1, besides being present in secretory membranes, localizes to PASs and the expanding phagophores (Pinar et al., 2013) and that, in agreement, Trs85, a diagnostic subunit of TRAPPIII, is present both on Golgi membranes and in the PASs (Pinar and Peñalva, 2020). Importantly, *A. nidulans* contains homologs of TRAPPC11, TRAPPC12 and TRAPPC13 (Pinar et al., 2019).

We show that Tca17 and TRAPPC11/12/13 are components of a TRAPPIII complex targeted to the autophagy pathway. Efficient autophagy requires both these four components and Trs85. TRAPPC11/12/13 form a stable subcomplex with Tca17. Tca17 binds to Trs33 and Bet3a (Choi et al., 2011; Galindo et al., 2021; Bagde and Fromme, 2022). This dual interaction of Tca17 with both Trs33 (the homolog of metazoan TRAPPC6) and Bet3a (the homolog of metazoan TRAPPC3a) explains that whereas *Aspergillus* Tca17 is essential for growth, Trs33 is not. Trs20, located at the TRAPPIII end opposite to Tca17, connects the core to Trs85, demonstrating that the organization of TRAPPIII in *A. nidulans* is akin to that in *D. melanogaster*, the only organism for which a three-dimensional structure is available (Galindo et al., 2021). Importantly, our studies strongly suggest that there are two co-existing versions of TRAPPIII, both containing Trs85 but with only TRAPPIIIb containing Tca17 and TRAPPC11/12/13, which are required to target efficiently the complex to the PAS. TRAPPIIIa, which does not contain these four proteins, would play its role in the secretory pathway.

Somewhat paradoxically, considering that fungal growth is exquisitely dependent on the efficient functioning of the secretory pathway, ablation of Trs85 results in a weak growth phenotype, despite the fact that TRAPPIII is involved in transport between the ER and the Golgi. Our finding that *trappc11*Δ shows a synthetic negative interaction with *trs85*Δ strongly suggests that in the absence of the latter, TRAPPC11, but not TRAPPC12 or TRAPPC13, are able to perform partially the role of Trs85. Trs85 anchors the complex to membranes (Joiner et al., 2021). Thus, this interaction is consistent with the possibility raised by Galindo and Munro that TRAPPC11 shares the ability to anchor the complex to membranes with Trs85 (Galindo and Munro, 2023).

Mutations affecting human TRAPPC11, TRAPPC12 and TRAPPC2L cause a complex spectrum of severe developmental and neurological disorders (Milev et al., 2017, 2018; Ramírez-Peinado et al., 2017; Sacher et al., 2019). An α-helix in fungal, plant and metazoan TRAPPC2L homologues containing a conserved Leu-Asp-Φ motif is crucial for its interaction with TRAPPC11 (Fig. S3; Sacher et al., 2019; Galindo et al., 2021). In humans, a homozygous missense mutation resulting in an Asp37Tyr substitution within this motif causes a developmental disorder, but this mutation is relatively uninformative in terms of understanding the basis of the disease at the molecular level, as it affects both TRAPPII and TRAPPIII through interaction with TRAPPC10 (known as Trs130 in yeast) and TRAPPC11, respectively (Galindo et al., 2021). In contrast, *A. nidulans* can be a useful model for the functional analysis of non-synonymous mutations in TRAPPC11, which might help to improve the prognosis of the spectrum of diseases caused by pathogenic variants in this gene.

The finding that two stable subcomplexes containing Tca17 are present in *Aspergillus* is important. One contains Trs120, Trs130 and Trs65 and, when combined with core TRAPP, gives rise to TRAPPII, targeting TRAPP to the trans-Golgi network. The second contains Tca17, TRAPPC11, TRAPPC12 and TRAPPC13, and, when combined with TRAPPIIIa, gives rise to TRAPPIIIb, thereby targeting TRAPP to autophagosomes.

## MATERIALS AND METHODS

### *Aspergillus* techniques

For strain propagation and harvesting of conidiospores, we used standard *Aspergillus nidulans* media (Cove, 1966). Deletion and epitope-tagged alleles were introduced into appropriate genetic backgrounds by meiotic recombination and/or transformation. For the latter, we used *nkuA*Δ strains deficient in the non-homologous end-joining pathway as recipients (Nayak et al., 2006). The complete genotypes of all strains are detailed in Table S1.

### Null mutant strains and protein tagging

*trs33*Δ, *trs85*Δ, *trappc11*Δ, *trappc12*Δ and *trappc13*Δ were generated by gene replacement, using as donor DNA molecules cassettes constructed by fusion PCR (Szewczyk et al., 2006). A gain-of-function *rab11* allele (denoted rab11*) was used to rescue lethality resulting from *tca17*Δ (rational reported in Pinar et al., 2019). Integration events were confirmed by PCR with external primers. The following proteins were C-terminally tagged endogenously: Trs85–3×GFP (Pinar and Peñalva, 2020), TRAPPC11–3×GFP, TRAPPC11–HA_3_, TRAPPC11–Stag, TRAPPC12–GFP, Bet5–Stag, Trs85–HA_3_ and Trs23–HA_3_ (Pinar et al., 2015; Pinar et al., 2019). The oligonucleotides used to construct these cassettes are listed in Table S2. GFP–Atg8 and mCherry–Atg8 were expressed under the control of *gpdA^mini^* promoter (Pinar et al., 2013).

### Autophagy assays

GFP–Atg8 processing experiments were carried out essentially as described previously (Pinar et al., 2013), *A. nidulans* strains were cultured for 13–14 h at 30°C on synthetic complete minimal medium supplemented with 1% (w/v) glucose as carbon source and 40 mM ammonium sulfate as nitrogen source. Mycelia were harvested by filtration, and an adequate proportion was freeze-dried (zero time point). The remaining mycelia were transferred to flasks containing the above medium without any nitrogen source, to induce autophagy. Mycelial samples were collected every hour for up to 3 h and freeze-dried. 5 mg of each freeze-dried mycelial lyophilizate was ground to a fine powder with a ceramic ball using a FAStprep-24 (MP Biomedicals) and 2-ml screw-cap tubes. Total protein extracts were obtained by alkaline lysis (Hervás-Aguilar and Peñalva, 2010). 10 μl of each sample was run through 12% SDS-polyacrylamide gels before being electro-transferred to nitrocellulose filters, which were reacted with anti-GFP antibody (1:4000; Merck cat. no. 11814460001) and HRP-coupled anti-mouse-IgG (1:5000; Jackson Immunoresearch cat. no. 115035003) as primary and secondary antibodies, respectively. The protein signal was detected with Clarity western ECL substrate (Bio-Rad) and the chemiluminescence was recorded with a Bio-Rad Chemidoc Imaging System (Fig. S5).

### Determining levels of TRAPPC11 upon inducing autophagy

Conidiospores from a strain expressing TRAPPC11–HA_3_ were cultured for 14 h at 30°C on synthetic complete minimal medium with 1% (w/v) glucose as carbon source and 20 mM ammonium sulfate as nitrogen source. Mycelia were harvested by filtration and transferred to the above medium without ammonium sulfate. Samples of mycelia were collected every hour for 4 h; total proteins were extracted as described above and analyzed by western blotting. TRAPPC11–HA_3_ signal was detected with rat anti-HA primary antibody (1:1000; Merck cat. no. 118674423001) and HRP-coupled anti-rat IgM plus IgG secondary antibody (1:4000; Southern Biotechnology cat. no. 301005). α-Tubulin, used as a loading control, was detected with anti-α-tubulin antibody (1:5000; Merck cat. no. T9026) and HRP-coupled anti-mouse-IgG antibody (1:4000; Jackson Immunoresearch cat. no. 115035003) (Fig. S5).

### Fluorescence microscopy

*In vivo* fluorescence microscopy experiments were carried out on μ-Slide 8-well uncoated chambers (Ibidi), with 0.3 ml of ‘watch minimal medium’ (WMM) (Peñalva, 2005) per well, which were inoculated with conidiospores suspensions and incubated overnight at 26–28°C. The microscope setup consisted of a Leica DMI6000B inverted microscope, a Leica 63×/1.4 N.A. plan apochromatic objective coupled to an objective heater (Peacon) adjusted to 28°C and a Hamamatsu ORCA-ER CCD (1344×1024 pixels, 6.45 μm cell size) camera. The hardware was driven by Metamorph Premier software (Molecular Dynamics) and coupled Single-channel acquisition of fluorescence images was made with Semrock GFP-3035B and TXRED-4040B ‘BrightLine’ filter cubes. All colocalization experiments were made with images acquired simultaneously in the two channels with a Photometrics Dual-View beam splitter (CCD camera). *Z*-stacks of images acquired with a *Z*-pass of 0.5 μm were transferred to the computer RAM using the ‘stream acquisition’ function of Metamorph to minimize the time elapsed between acquisition of the first and last planes of the stack. *Z*-stacks were deconvolved using Huygens Professional software (Hilversum). Maximum intensity projections (MIPs) of the red and green channels were merged using the ‘color align’ Metamorph plug in. Images were annotated with Corel Draw.

### Reproducibility of quantitative colocalization data

For the colocalization data in Figs 2D and 3A, we ensured results were reproducible across experiments. For all colocalization experiments involving combinations of TRAPPC11–GFP, TRAPPC12–GFP and Trs85–GFP with mCherry–Atg8 in the wild type and various mutant backgrounds, eight independent gene-replaced clones were screened by fluorescence microscopy to confirm the reproducibility of the observed phenotypes, irrespective of whether they were obtained by transformation or by genetic crossing. Quantitative data were obtained from two biological replicates, except for the­ TRAPPC11–GFP and Trs85–GFP colocalization experiments with the mCherry–Atg8-labeled PAS in the wild-type background, for which four biological replicates were analyzed. For those mutants in which the GFP reporter was not detectable in the PAS, the presence of the transgene was verified by diagnostic PCR.

### Superose 6 size exclusion chromatography

Strains were cultured on *Aspergillus* fermentation medium (MFA) consisting of minimal medium supplemented with 2.5% (v/v) of corn steep liquor syrup (Solulys 048R, Roquette Laisa S.A., Valencia, Spain), 50 mM each of Na_2_HPO_4_ and NaH_2_PO_4_, 50 mM NaCl, 3% (w/v) sucrose as the main carbon source, 20 mM (NH4)_2_SO_4_ as the main nitrogen source and vitamins as required to supplement the auxotrophic mutations present in each strain. After 13–14 h of incubation at 30°C with shaking, a mycelial sample was collected by filtration and freeze-dried (N-sufficient conditions). The rest of the mycelium was transferred to synthetic complete minimal medium without nitrogen and further incubated for 1 h at 30°C before harvesting and freeze-drying the resulting biomass (N-starved conditions). 80 mg of each freeze-dried mycelial sample were ground in 2 ml crew-cap microcentrifuge tubes containing a ceramic bead with a 20 s pulse of FastPrep set at power 4. The powder was resuspended in 1.5 ml of lysis buffer containing 25 mM Tris-HCl, pH 7.5, 0.5% (v/v) NP40, 600 mM KCl, 4 mM EDTA, 1 mM DTT, 5 μM MG132 and Complete ULTRA EDTA-free protease inhibitor cocktail (Roche). Next, ∼100 μl of 0.6 mm glass beads was added to the suspension, which was homogenized with a 15 s pulse at full power of the FastPrep followed by incubation for 10 min at 4°C in a rotating wheel. This step was repeated two additional times before the resulting homogenate was centrifuged at 15,000 ***g*** and 4°C in a refrigerated microcentrifuge. 200 μl of the supernatant were loaded onto a Superose 6 10/300 column (Cytiva) equilibrated in lysis buffer without protease inhibitors, which was run at a flow rate of 0.5 ml/min in a Biorad NGC chromatography system. Fractions of 0.5 ml were collected. The column was calibrated with the following standards: aldolase (158 kDa), ferritin (449 kDa) and thyroglobulin (669 kDa). The exclusion volume (V_o_) was determined with Dextran Blue (Pharmacia Fine Chemicals). 22 fractions of 0.5 ml starting from the excluded volume were analyzed: 80 μl of each were mixed with 40 μl of 2× Laemmli loading buffer, denatured at 100°C and 25 μl of the mixture were loaded onto 8% (for TRAPPC11–HA_3_ or Trs85–HA_3_ detection) or in 12% (for Trs23–HA_3_ detection) SDS-PAGE gels, transferred to a nitrocellulose membrane and analyzed by western blotting using anti-HA rat monoclonal 3F10 antibody (1:1000; Merck cat. no. 118674423001) and HRP-coupled goat anti-rat-IgG (1:4000; Southern Biotechnology cat. no. 301005), as primary and secondary antibodies, respectively. HRP signal was detected with Clarity western ECL substrate (Bio-Rad). Chemiluminescence was recorded with a Bio-Rad Chemidoc Imaging System (Fig. S5), and the signal in anti-HA-reacting bands was quantified with Image Lab 5.2.1 (Bio-Rad) and plotted with SigmaPlot using the ‘multiple Spline Curve’ option of the Graph menu.

### S-agarose purification of TRAPPs under N-sufficient and N-starved conditions

Strains expressing Bet5–Stag or TRAPPC11-Stag were cultured for 14–15 h at 30°C on MFA medium before harvesting the mycelia by filtration and dividing the resulting biomass into two halves. One half was freeze-dried. The second was transferred to a minimal medium without nitrogen and incubated for 1 h at 30°, filtered and freeze-dried. 1.75 g of each freeze-dried sample was ground with ceramic beads in a Fast Prep. The powder was resuspended with 45 ml of extraction buffer [25 mM Tris-HCl, pH 7.5, 0.5% (v/v) NP40, 200 mM KCl, 4 mM EDTA, 1 mM DTT, 5 μM MG132, Complete ULTRA EDTA-free protease inhibitor cocktail (Roche)]. Then 5 ml of 0.6 mm glass beads was added, and the mixture was homogenized with a 15 s full-power pulse of the FastPrep followed by incubation for 10 min at 4°C in a rotating wheel. This step was repeated two additional times before centrifuging the resulting homogenate at 15,000 ***g*** for 30 min at 4°C and collecting ∼35 ml of supernatant, which was spiked with 1% (w/v) BSA.

Then, 0.5 ml of S-protein agarose beads equilibrated with extraction buffer containing 1% BSA was added to each extract. The mixture was incubated for 2 h at 4°C in a rotating wheel. Beads were collected by centrifugation at 4°C at 300 ***g*** in a tabletop centrifuge and washed four times for 10 min at 4°C with 10 ml of washing buffer (25mMTris-HCl, pH 7.5, 300 mM KCl, 4 mM EDTA, 1 mM DTT). For protein elution, the resin was transferred to a 2 ml Eppendorf tube and resuspended in washing buffer containing 4 mg/ml of S-peptide. The mixture was incubated at 37° for 15 min with gentle agitation, and the supernatant was collected by centrifugation (300 ***g*** for 1 min). This step was repeated a second time. The eluate was precipitated with 10% TCA and the pellet was resuspended with 60 μl of Laemmli buffer. Half of the sample was loaded onto a 10% SDS-polyacrylamide gel that was run until proteins moved ∼1 cm into the separation gel. The portion of the gel containing the protein mixture (identified after staining with colloidal Coomassie) was excised and processed for MS/MS analysis.

### GFP-trapping and MS/MS

Conidiospores from wild-type or *trs33*Δ strains were inoculated in flasks containing MFA, which were incubated overnight at 30°C. The mycelium was then filtered and transferred to nitrogen-free minimal medium for 1 h at 30°C, filtered and freeze-dried. Cell extracts were prepared as described above, but using lysis buffer containing 25 mM Tris-HCl pH 7.5, 150 mM NaCl, 0.5 mM EDTA, 0.5% NP40, Complete ULTRA EDTA-free protease inhibitor cocktail (Roche) and 5 μM MG132. 4 ml of extract (∼100 mg of total protein) were immunoprecipitated with 25 μl of GFP-Trap magnetic agarose beads (Chromotek) for 2 h at 4°C in a rotating wheel. Beads were washed four times with the same buffer. For MS/MS analysis, the proteins immobilized on the resin were digested ‘on-beads’. The beads were resuspended in 25 μl of buffer I [25 mM Tris-HCl pH 7.5, 2 M urea, 100 ng trypsin (Pierce Trypsin Protease MS-Grade, Thermo Fisher Scientific)], and 1 mM dithiothreitol, and incubated in a thermomixer at 37°C and 400 rpm for 30 min. The beads were pelleted on a magnetic stand, and the supernatant was transferred to a fresh tube. Pelleted beads were resuspended in 50 μl of elution buffer II (50 mM Tris-HCl pH 7.5, 2 M urea and 5 mM iodoacetamide), and pelleted a second time to recover the supernatant, which was combined with the first one. This step was repeated a second time before combining the supernatants and incubating them in a thermomixer at 32°C and 400 rpm overnight protected from light. The digestion was terminated by adding 1 μl of trifluoroacetic acid and the sample dried in a speed vacuum centrifuge. The resulting peptides were purified on a Zip Tip with 0.6 μl of C18 resin (Merck), dried and resuspended in 10 μl of 0.1% formic acid in water. A total of 4 μl of each sample was subjected to nano-LC ESI-MS/MS analysis using a Vanquish Neo nano system (Thermo Scientific) coupled to an Orbitrap Exploris 240 mass spectrometer (Thermo Fisher Scientific). Samples were loaded onto an Acclaim PepMap 100 precolumn (Thermo Fisher Scientific), and eluted in a RSLC PepMap C18, 50-cm long, 75-μm inner diameter and 2-μm particle size. The mobile phase flow rate was 250 nl/min using 0.1% formic acid in water (solvent A) and 0.1% formic acid and 80% acetonitrile (solvent B). The gradient profile was set as follows: 4% solvent B for 2 min, 4–31% solvent B for 93 min, 31–50% solvent B for 7 min, 50–100% solvent B for 1 min and 100% solvent B for 15 min. For ionization, a liquid junction voltage of 1900 V and a capillary temperature of 275°C were used. The full scan method employed a *m*/*z* 350–1200 mass selection, an Orbitrap resolution of 60,000 (at *m*/*z* 200), a normalized target automatic gain control (AGC) value of 300% and maximum injection time in automatic mode. After a survey scan, the 20 most intense precursor ions were selected for MS/MS fragmentation. Fragmentation was performed with a normalized collision energy of 50% and MS/MS scans were acquired with a starting mass of *m*/*z* 120, AGC target was custom, resolution of 15,000 (at *m*/*z* 200), intensity threshold of 1.0×10^4^, isolation window of 1.0 *m*/*z* units and maximum injection time in automatic mode. Charge state screening was enabled to reject unassigned, singly charged, and equal or more than seven protonated ions. A dynamic exclusion time of 10 s was used to discriminate against previously selected ions.

### MS/MS data analysis

MS data were analyzed with Proteome Discoverer (version 3.0.0.757, Thermo Fisher Scientific) using the Mascot search engine (version 2.6, Matrix Science). Searches were performed against *Aspergillus nidulans* FGSC A4 version_s10m02-r03_orf_trans_allMODI. (10,489 protein sequence entries). Precursor and fragment mass tolerance were set to 10 ppm and 0.02 Da, respectively. Trypsin/P was selected as the protease, with a maximum allowance of two missed cleavage sites, Cys carbamidomethylation as fixed modification, with N-terminal acetylation, Asn or Gln deamination, methionine oxidation and N-terminal pyrrolidonation of Glu and Gln as variable modifications. Identified peptides were filtered using Percolator algorithm with a q-value threshold of 0.01. For Fig. 3 the abundance of each protein hit was given as the number of PSMs for each TRAPP protein divided by the number of Lys+Arg residues present in the protein sequence and multiplied by 100. For Fig. 2J,K involving parallel purifications from wild-type and mutant cells these scores were plotted as a percentage relative to the wild-type.

## Supplementary Material



10.1242/joces.264822_sup1 Supplementary information
