# Supplementary material for: A subcomplex comprising TRAPPC11, TRAPPC12, TRAPPC13 and the fungal TRAPPC2L homolog, Tca17, directs TRAPPIII to autophagy
Source: J Cell Sci. 2026 Aug 27;139(16):jcs264822. doi: 10.1242/jcs.264822 (PMC13571812; doi:10.1242/jcs.264822)
Supplement: Supplementary file 1 [file joces-139-264822-s1.pdf]

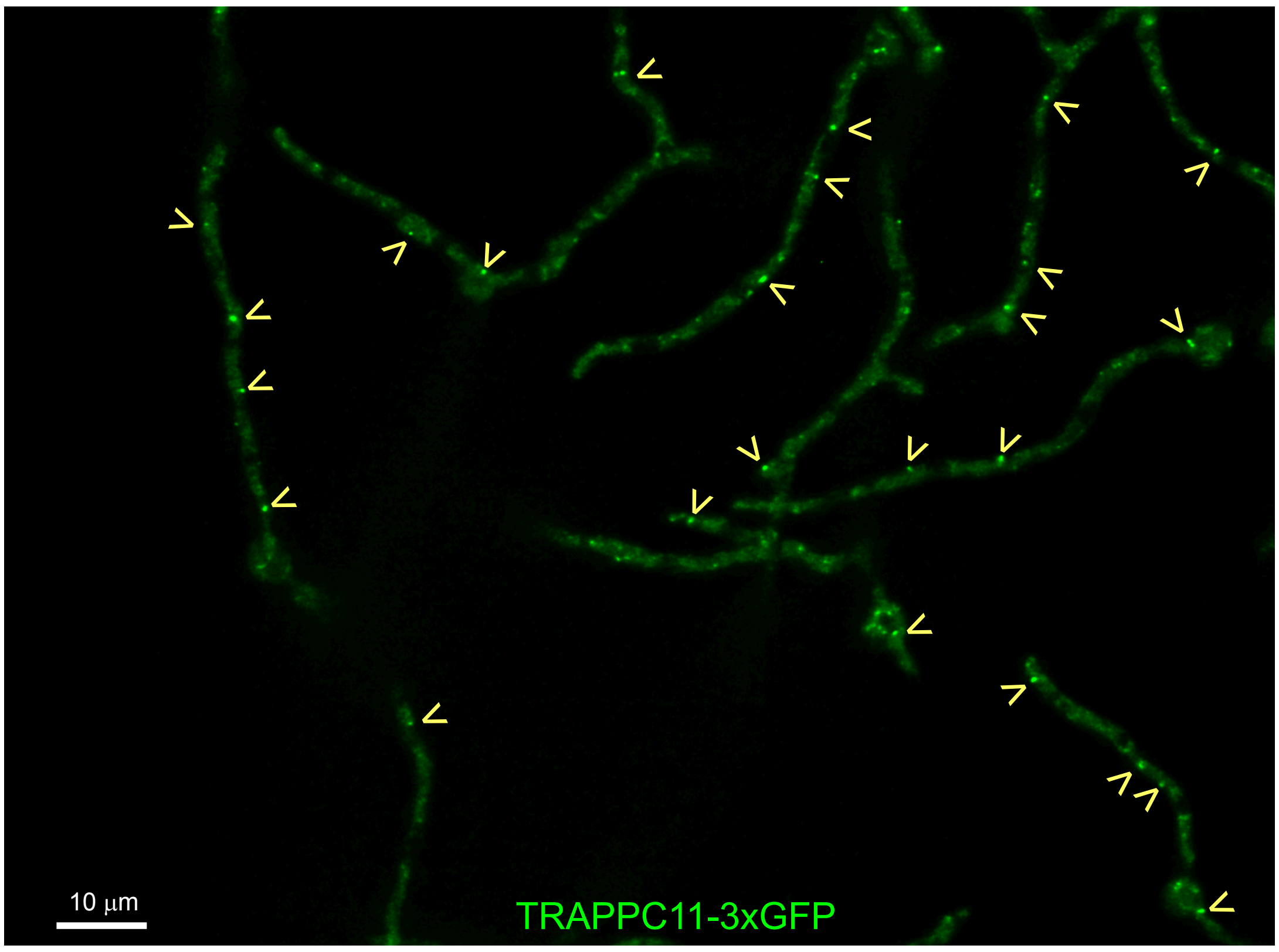

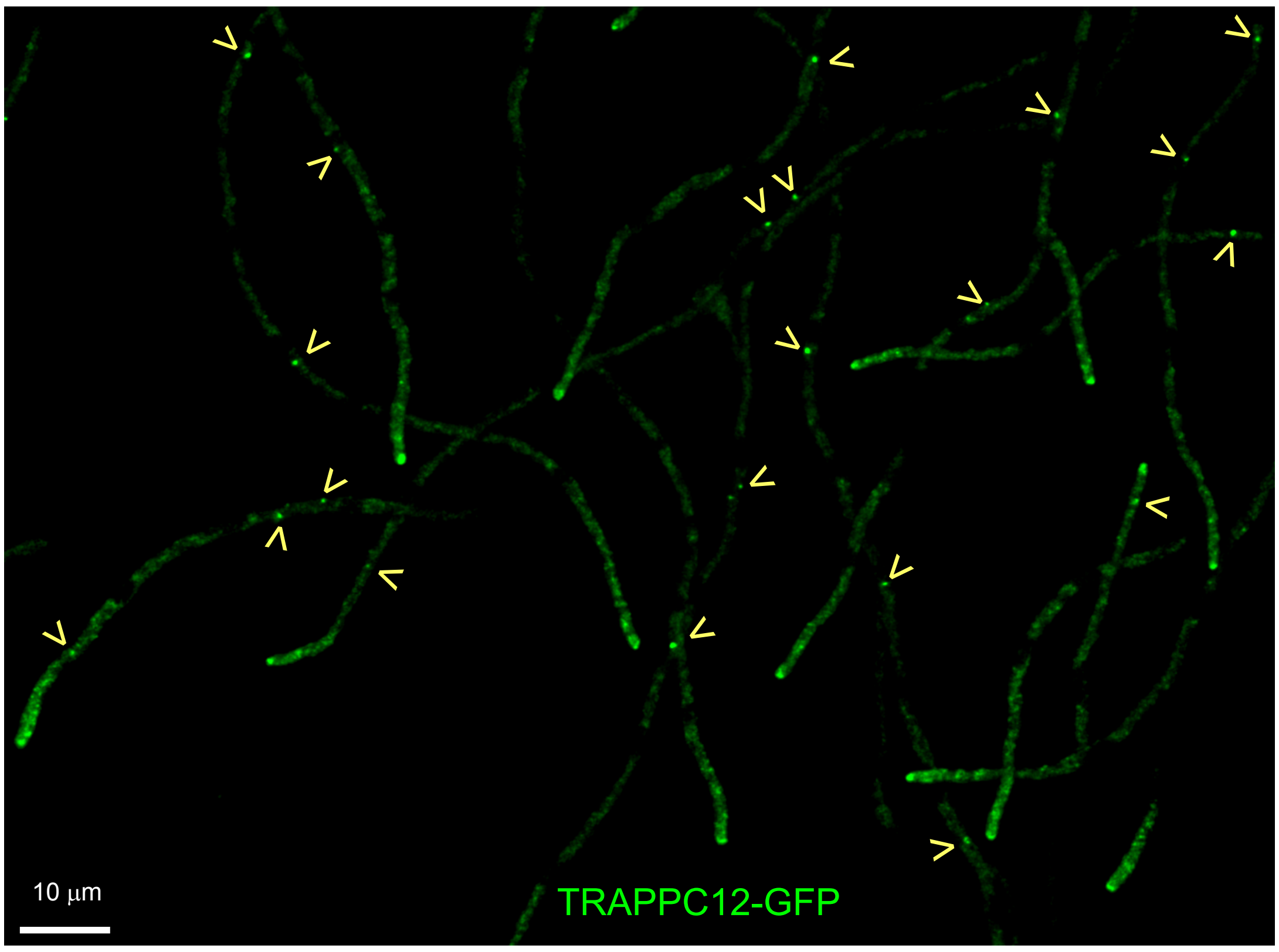

Fig. S1. Localization of GFP-tagged TRAPPC11 and TRAPPC12 to secretory compartments and to the PASs. The latter are indicated by arrowheads.

Journal of Cell Science • Supplementary information

```
                 *        20         *        40         *        60         *        80         *       100         *       120         *       140         *       160
HS : MSPTQWDFPVELCCRPMAFVTLTGLDVVYNAVH--------------------------RAVWDAFCAN---------------RRADRVPISFKVLPGDHEYP-----------------------------KCRPKRTSYEWYIPKGILKTGWMNKHL :   90
DM : MTMDATALPSELLVTPQPLVGFCGLDTARVSVH--------------------------KAVWEAFSGSLQR------------KAADRAAVQYKLLPPNYEFP-----------------------------VAKPKRASYEWYHPKGILKRNWMLKHL :   93
AT : ME----EYPEELRTPPVSLVALFGYAELHASITKY---------------LHSQQPPINALAFPDFSQISLLL----AHDDQISRT-----------SSFRDP-----------------------------LSVSDSASPIPSRCGGILKRDWLLKHR :   96
AN : MD----DYPRPYIANNLPFILLCGLEADGDRTEPEAEETNYPLLEENGIAIESDLPPLERPVAEEFRDVFFSEAGPRSQDNGIEGTSRAGYKVKSIGRNYRLPPRKANPPPASPPESPTVSRDHGLSMILHSPISPLTPA--SPTFPDGIMTPLWASKHQ :  154

                 *       180         *       200         *       220         *       240         *       260         *       280         *       300         *       320
HS : NLVPALVVVFYELD---WDEPQWKEKQSECATRVEIVRQS--LQGRNTKVAVVLIQKKTPLPPGEDVIA-SERAAALCNACELSGKSLFVLPH--TD-HLVGYIIRLENAFYEHAQTYYYTEIRRVKSHKEFLN------------K--TTHQLLFVRHQ :  227
DM : HVLPSVVVLFQDME---WNDLQWTEKQVQCAAIVQALKNT--LQERNTRLCLVLLQRAAPLPPGEDLLA-AERAASLTNACGITSKMLFILPH--TE-HLTGYALRLESAFLDMAQSYYALMSKRIRNHRDQLT------------A---AHTSLKIRHQ :  229
AT : TKVPALVAAFFPSHHIFGDPTQWL----QVCSDLDSLKSV--IRPKNIKLVVVVVQS-S---PHEDIS--DDRLVALRKRAELDSKYVLFFNSSIVS-ELTLSLSRLASAFAELALSYYREEGRRIKSRIEKRS---------------SNSLDLNVRYC :  228
AN : DLVPAAVINFFPFC---LDSTMNSLRDNQLKIEINGLKKQWSSSGYRTRFIVVLLSEKA---IGDDLGDLDDRVLGIRRATNLDQKSLFILPPDVSSEEMRDFTRSLLALLHPSLMEYYRDLSKHARRKRNRSSIPPPTAPPTSGTSQTLSLQGWNTRYE :  308

                 *       340         *       360         *       380         *       400         *       420         *       440         *       460         *       480
HS : FKIAFFSELKQDTQNALKNYRTAYNLVHELRAH------ETNILEIKTMAGFINYKICRLCFQHNTPLDAIAQFRKHIDLCKK---KIGSAELSFEHDAWMSKQFQAFGDLFDEAIKLGLTAIQTQ------------------------------NPG :  347
DM : FKLGFVAEMRQDFSTGQKHYFQAYANLDEIRIN------DGNCLEIKTLAGFLNYKICRLMFKLKTPRDAINQFIIHVEKHKS---RVGFKDLAFEHHAWLSTQHSVFAELFCEAIKNGLPALQTQ------------------------------HPG :  349
AT : FKVAVYAEFRRDWGEALKFYEDAYHSLHEMIGTSTRLPAIQRLVEIKIIAEQLHFKISTLLLHGGKLIEAVTWFHQHKTSYEK---VVGSTEFIFLHWDWMSRQFLVFAELLETSSATGQSLT-SSNQGT-A---------------EISL-TEFEFYPA :  367
AN : FKMGIFAEFRQEIDAALKNYESAYETLFGQEVFENIAGWDPKFNEARLFSDALAIRIIRCLLSTGHTSSAVRSWVYHRRRTQDIVNRRGKGTRNYGWEAWEARWSSVMAQLIHQAKIPHLTPLETSQNQVHEHNSIFVPPEKTFLSVDEVLPWERLHHEG :  468

                 *       500         *       520         *       540         *       560         *       580         *       600         *       620         *       640
HS : FYYQQAAYYAQERKQLAKTLC-NHEASVMYPN-PDPLETQTGVL-DFYGQRSWRQGILSFDLSDPEKEKVGILAIQLKERNVVHSEIIITLLSNAVAQFKKYKCPRMKSHLMVQMGEEYYYAKDYTKALKLLDY--VMCDYRSEGWWTLLTSVLTTALKC :  502
DM : IYYHKAAEFVMKRRDAAMEAY-AAMQASSEAT-PTPIQNPLSLYTEFFGIRAVKT----GDLVA---EQQANMQLCDQERSYNHSAAIIALLSQAMAQFKIYKCLRFRKKLAIDMAEEYLKSGDHAKALTLYSL--MLPDYRQEKWTTIFTDVLLKTLRC :  498
AT : YYYQLAAHYLKDKKS-ALELLLSMSEIAQEIDSSSASITPSVYVGQFAQLLEKGEAITLHSITD---EEYTRYTI-SEAKRVQDSLQIIAWLKRSYESFTNLKAQRMAALCAFEVAREYFDLADPNNAKFFFDI--AANLYRQEGWVTLLWEVLGYLREC :  520
AN : YWLYRSAKHTMHRRTLAGQIP-AEDR-------MPPGQSPASHIASKAYLYDT-------YLAP----ETHIEAARAQDGGVDHSTLILDTLKAALVEFSKRNQVRMTESLSLEIAEEYMRLGSWIEAYETLRPLWPALTWRRSGWWLIMAKFAWILREC :  609

                 *       660         *       680         *       700         *       720         *       740         *       760         *       780         *       800
HS : SYLMAQLKDYITYSLELLGRASTLKDDQKSRIEKNLINVLMNESPDPEPDCDILAVKTAQKLWADRI-----------SL----AGSNIFTIGVQ---DFVPFVQCKAKFHAPSFHVDVPVQFDIYLKA---DCPHPIRFSKLCVSFNNQE--------- :  632
DM : ALLSGSVADYIACSVEALSLRHQSDQSERILILENLWQVFQGVPPMPKTQL----TPEAQALWTSAL-----------AN----VKSP-IQIDLD---KVNDVVEMCATFERVQLSNDDLLQLQLIVRV---LTDIPLRIRSFHVILADAGNP---QNSY :  629
AT : SRNLDALKDFVEFSLEMVALPVTSYENSGNLRNKNYG---PGGPATISGRES-----IHQEVFTLVCREAELLSSTEGSGFKLATDSP-LHLEIDLVSPLRPVLLASVAFHDQMIKPHALCSFTLSLLS---HLPLPVEIDHLEVQFNQST--------- :  659
AN : AARAQDSETVLRVDWELLNFAFPPRPD---------------------WNYD-----IHQSLAEFAS-----------KE----QKPS-IVIKAE---DVVSSVTATFIFQKAEGNVGEPLQCQLVLRSCAQKSSVPVRFAELRVAFEGCLRPIRLQSDQ :  724

                 *       820         *       840         *       860         *       880         *       900         *       920         *       940         *       960
HS : -----YNQFCVIEE-------ASKANEVLENLTQ---------GKMCLVPGK---------------------------TRKLLFKFVA----------------------KTEDVG--------KKIEITSVDLALGNETGRCVVLNWQGGGGDAASS :  713
DM : KLEA-LKYFCFPTLTQLRGQKQP-DDEQLENPSQEPKNFE---KNMRLEPGS---------------------------YYQLFCSTEA----------------------QQFHEN--------TQLRIVRLEAHMGTDQVAALL---------TCSS :  717
AT : -----CN-FV-I-RNSQRPLWASA-SNTV-KSGS---QVE-NAPLLVLVPNN---------------------------WLRLTYAI------------------------KSEQS---------GKLECLSVLAKLGPLFTICSR-------AESPAA :  737
AN : NIDADTSTTCLISSPSLRDPSATS-DSSVHQSTTSALNALTGIADLTLGPAQTKVYNLACIPRESGESRVASIALLVSQEDFDLTYAITDLAQQEAFWWQQTTKGPARRRVGKGRDVDRCKIQPKPPKIRLTIPNLKSTYYTNERIVLNLVIDNGEDEA- :  882

                 *       980         *      1000         *      1020         *      1040         *      1060         *      1080         *      1100         *      1120
HS : QEALQAARSFKRRPKLPDNEVHWDSIIQASTMIISRVPNIS---------VHLLHEPPALTNEMYCLVVTVQSH-EKTQIRDVK-------------------------------------------LTAGLKPGQDAN---LTQ-KTHVTLHGTE- :  813
DM : NYSRQLFRHHTRSRDLDDN-VTINPICYIAPTFHLDTQTNLGHGHDNGLTDDKEMVATKMLVNEYFPVVTTVSNP-YNVYLQNVGVHISIPVGLRNSVFLTTDISPGRQKLHSQIQIDVGELSAHGSNTATFYIFSLTEAEIKLAQRL-SYTLDVDRGPG :  874
AT : MEDLPVWKHENRVESLPTKDP-VLAVFGQKATQVDEPEPQVD---------VSLGASGPALVGEDFAMPIVVTSKGHAVYSG-------------------------------ELKI-------NLVDVGGGGLFSPREAEP---FSLESHHVEICGIDG :  846
AN : ADVVAEARLFG-------------------------------------------------------------SPDS------------------------------AVNI-------SWLDQ-EGSVQASTESRP------------------ :  918

                 *      1140         *      1160         *      1180         *      1200         *      1220         *      1240         *      1260         *      1280
HS : -----------LCDESY---PAL---------LTDIPVGDLHPGEQLEKMLYVRCGTVGSRMFLVYVSYLINTT-VEEKEIVCKCHKDETVTIETVFPFDVAVKFVSTKFEHLERVYADIPFLLMT------------------------------------- :  915
DM : GGGSNARVSTAPNSSESTPDHEVKSVIPSVAAISPVQIEYLD-----------ETR-------------------------LRKSREDTLTVRCYGDFKFNARFYTMDRKPLNQVYRGENFLLRANTEVVAVDDVEILDSFFICDHNLVQSNYSFKQKK :  997
AT : A---------EGNNESESETGSIKKI---QQSFGLVSVPYLKEGESWSCKLEIKWHRPKPVMLFVSLGYLPHGS-EANTQ---KVHIHKSLQIEGKMPLLISNRFM--------LPYRRDHLLL-------NRIKPAPDSE-------DVSSLP------ :  962
AN : ------------NTPAEEEPHSLKRSVGVIESSSQREIPIVIS-----------GTESSFEYELEVSAAYNLFSDIQT----PIIATTRLTLPIIRPFEANYEF------------------L-------PRLNPLPWPDFFTIDDDLTETQP------ : 1019

                 *      1300         *      1320         *      1340         *      1360         *      1380         *      1400         *      1420         *      1440
HS : -----------------------------------------------------------------DLLSASPWALTIVSSELQLAPSMTTVDQLESQVDNVILQTG----------------ES-----ASE------------------ :  961
DM : YTNKYSAGEQLESVIVLRTNATLRDWATA--RDLDHRGKLEGKAVASKFIRPVPKPSSEEPLAPTPPAGISAYMSKSLVTKIPTT-------------MTIINSNSAVSNALQVANAGVMSNSTQSDDVNVAEE-----AINTHEGIKTTRLIYNKALE : 1136
AT : ------------------------LNEKSVLVVS----------------------------------AKNCSEIA------LKLVS---------MSIEFDDEQGETSCLIQQGGGCGDSPSSANLAPGEE-----FKKVFTVIPTTR--------- : 1033
AN : ------------------------NTEP--RGLQQRWCLDTK------------------------------VVSFAREP------LVIEN---------MSIAVLSLSG--GAISQVGHEVLVSPETREIHPEDLRHSNFVLDIQKIILGDR-------- : 1099

                 *      1460         *      1480         *      1500         *      1520         *      1540         *      1560         *      1580         *      1600
HS : ---------CFCLQC-PSLGNIEGGVATGHYIISWKRTSAMENIP-----IITTVITLPHVIVENI-PLHVNADLPSFGRVRESLPVKYHLQNKTDLVQD--VEISVEPSDAFMFSGLKQIRLRILPGTEQEMLYNFYPLMAGYQQLPSLNINLLRFPN- : 1102
DM : AVQGTGHCRGFIKKIYTLEGNPSAVPIFGVFCIRWRRANCKEE--------NESKFVIRGLDIAEP-PLNIYCTIEEKMFVKMPMAFKVVLKNPTTHVLHLIANLSISKTDNFICSGHKQLDISIMAYEEKELVYNLYPLQVGWQELPVLSLEYNTKADP : 1287
AT : ---------------------TPKLGLGSIHLKWRREGGNITEA-----YVSTKHKLPEVNVEAS-PLVMSLDSPPYAILGEPFTYAVRICNQTQLLQE--AKFGLADAQSFVLSGSHSNTVSVLPKSEHVLSYKLVPLTCGEQQLPKITLTSAR---- : 1159
AN : ---------------------RPTSLNLALEIQWRRSGEETDDSGRSDRLTTTKLAIPRFVIPAGEPRVLASAISSR-KFPGLIHVEYTLENPSLHFLT--FSLTMEASEYFAFSGPKTMVVQLAPVSRQTVRYNLLASKRGLWIQPQLVV------- : 1227

                 *      1620         *      1640
HS : --------FTNQLLRRFIPTSIFVKPQGRLMDDTS-----IAA-A : 1133
DM : QKKDSQNALLDELVQRALPKRVFVLPLPLKQQN-----------K : 1320
```

Fig. S2. Four-way amino acid sequence alignment of human, *Drosophila*, *Arabidopsis* and *Aspergillus* TRAPPC11. The foie gras domain is shaded in purple. The two regions that Galindo et al determined as being involved in the interaction with TRAPPC2L are boxed in yellow. Similar amino acids were according to the Blosum 45 score table.

Journal of Cell Science • Supplementary information

```
                 *        20         *        40         *        60         *        80
AN : MPGPKIACIGVIGKASDRLKDNPLHISLFPPYLGSAIDFSFLLNSSLDIFEIRQK------QTSVDQDLGLLQAVDERLASYGWITTTG :  83
HS : MA----VCIAVIAKE-----NYPLYIRS-T-PTENELKFHYMVHTSLDVVDEKISAMGKALVDQRELYLGLLYPTE-DYKVYGYVTNSK :  77
DM : MA----FCIAVIGKD-----NAPLYLTT-S-DMEQELELQYHVNAALDVVEEKCL-IGKGAPESKELYLGLLYSTE-NHKIYGFVTNTR :  76
AT : MI----VCVAVVGHQ-----NNPLYIQSFT-DADDALKLHHIVHCSLDVIEERVNNPKKSGTTLNEAFLGLLYPTV-NYKVYGYLTNTK :  78

       *       100         *       120         *       140         *       160         *       180
AN : LKFLIIVDLIGDQTSSVVNEVRGSVRAGVREADLKPAFRALQAAYIRLLQNPFYSPDDHAIINGAVQGTRPSQIT-DQRFIAEVNRIGN : 171
HS : VKFVMVVD---------------SSNTALRDNEIRSMFRKLHNSYTDVMCNPFYNPGDR--------------IQSSRAFDNMVTSMMI : 137
DM : VKFIVVID---------------SSNVALRENEVRAIFRNLHLLYTDAICNPFYIPGES--------------LT-SKKFDRAVQKLMS : 135
AT : VKFILVT----------------TDLDVRDTDVRSFFRKFHAAYVDAVSNPFHVPGKK--------------IT-SRTFAQTVSNIVG : 135

AN : SWGAVTSNV : 180
HS : QV--C---- : 140
DM : GT--A---- : 138
AT : SYGLN---- : 140
```

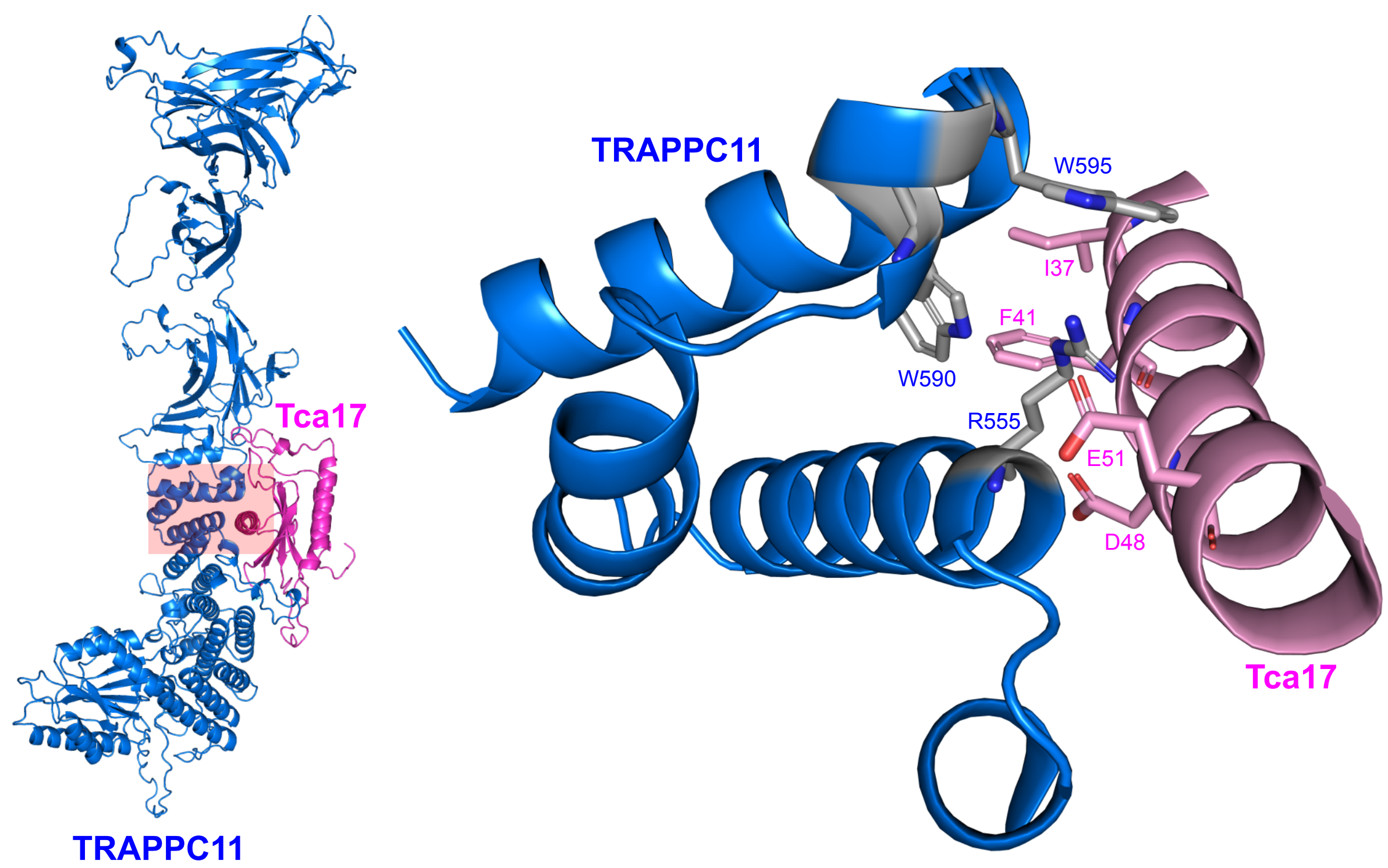

Fig. S3. Top, four-way amino acid sequence alignment of *Aspergillus*, human *Drosophila* and *Arabidopsis* TRAPPC2L homologues. The region containing the critical Leu-Asp-Φ motif is boxed (Φ indicates a hydrophobic residue). Similar amino acids were according to the Blosum 45 score table. Bottom, AlphaFold prediction of the interaction between the *foie gras* domain of *Aspergillus* TRAPPC11 and Tca17. Left, overall view of the predicted complex. Right, details of predicted interactions between the relevant region of the TRAPPC11 *foie gras* domain with α-helix 1 in Tca17. Asp48 within the conserved Leu-Asp-Φ motif corresponds to Asp37 in both *Drosophila* and humans.

Journal of Cell Science • Supplementary information

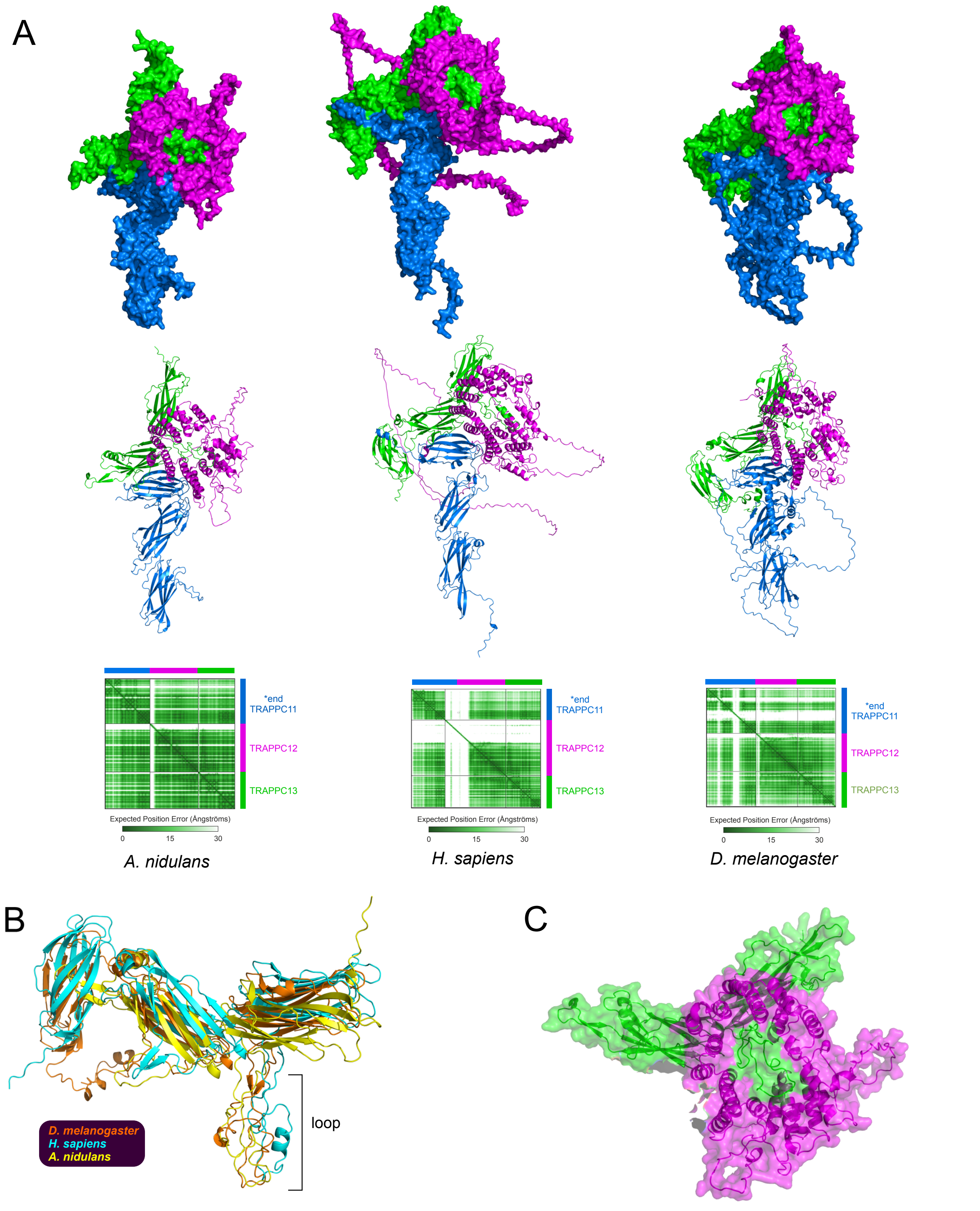

Fig. S4. AlphaFold predictions of the complex formed by the C-terminal half of TRAPPC11 with TRAPPC12 and TRAPPC13. (A) Predictions for the indicated complexes shown in surface and cartoon representations. (B) Structural alignment of TRAPPC13 from the three species showing the loop that is located between two beta-sandwiches. Note that *Aspergillus* TRAPPC13 contains only two beta-sandwiches, whereas the human and *Drosophila* proteins contain three. (C) AlphaFold prediction of the loop of *Aspergillus* TRAPPC13 inserted through the central channel of the TRAPPC12 alpha barrel.

Figure 1C-Source data

Figure 1C-1

α-GFP original images

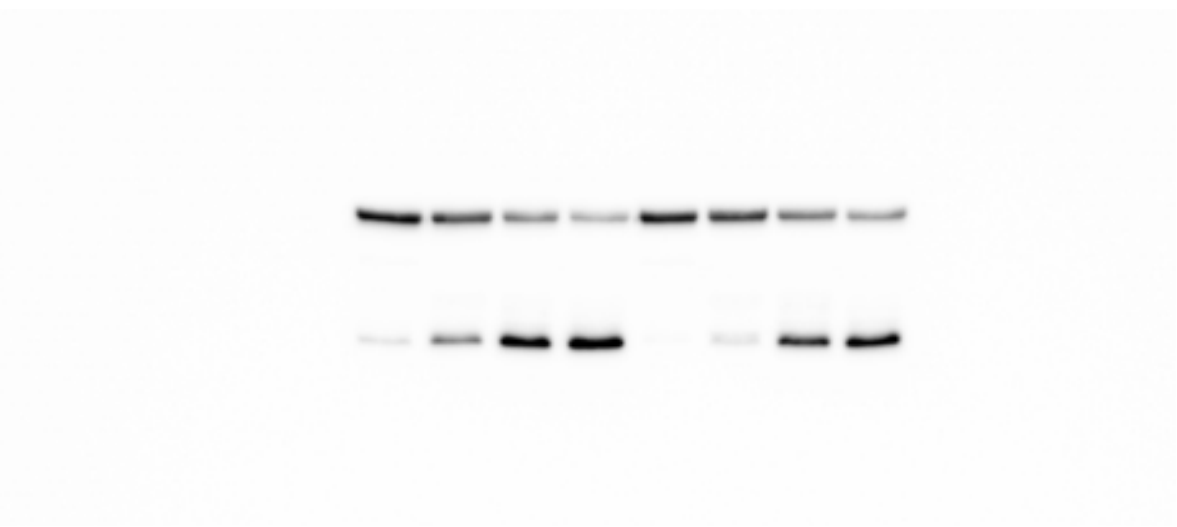

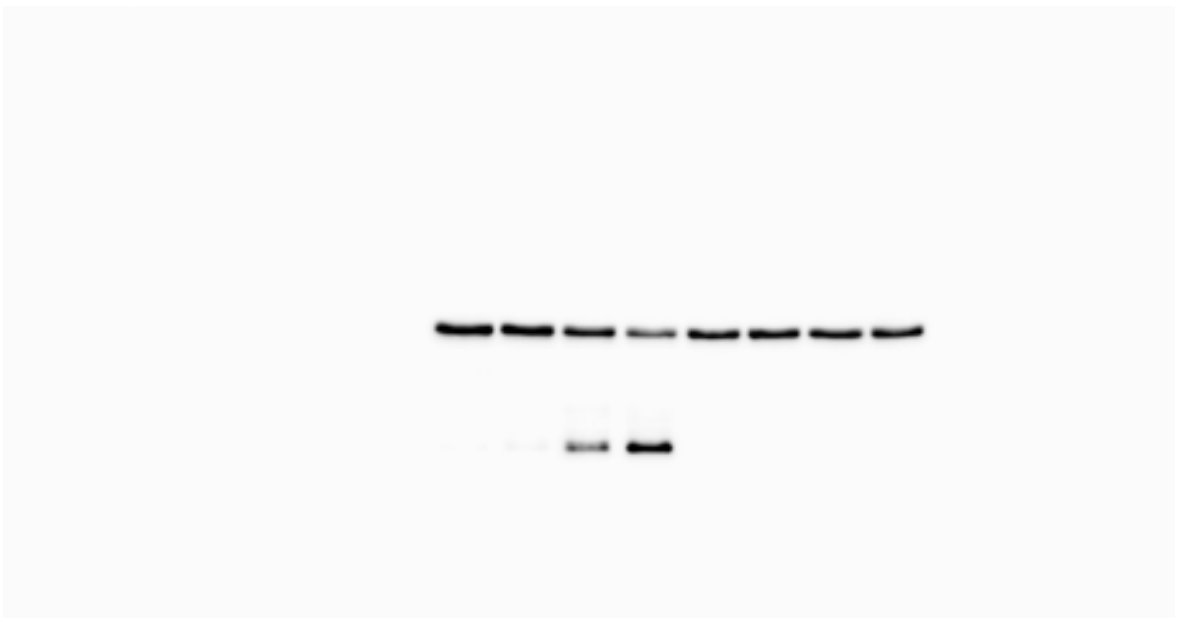

Used areas

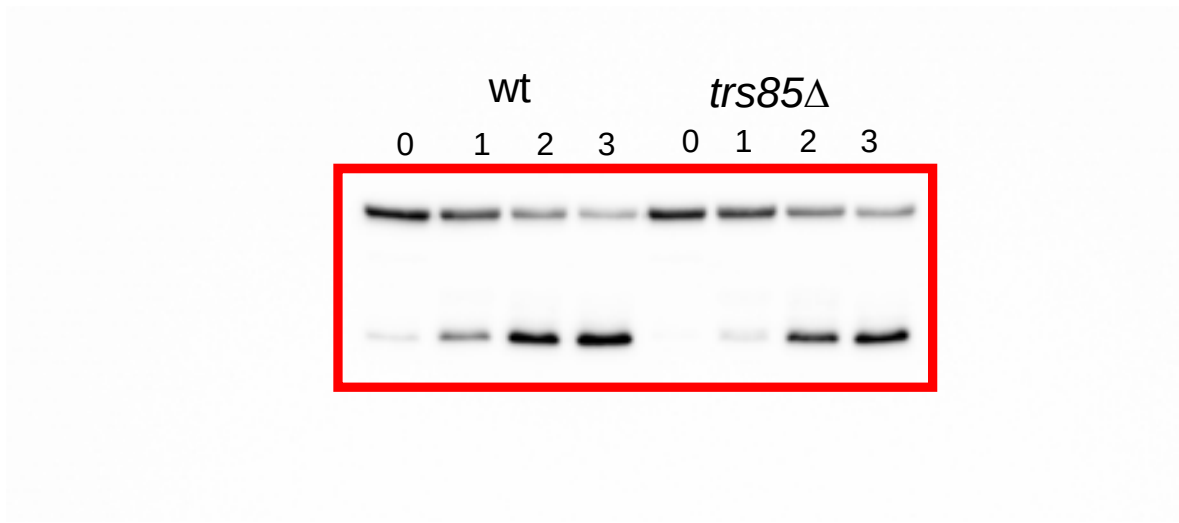

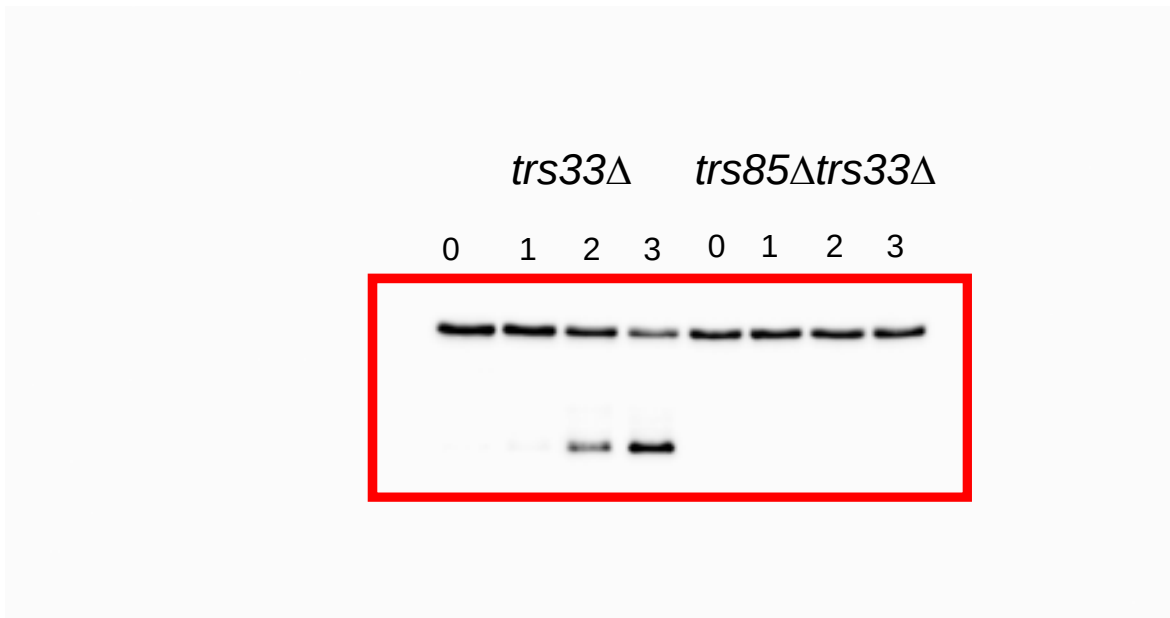

Figure 1C-2

α-GFP original images

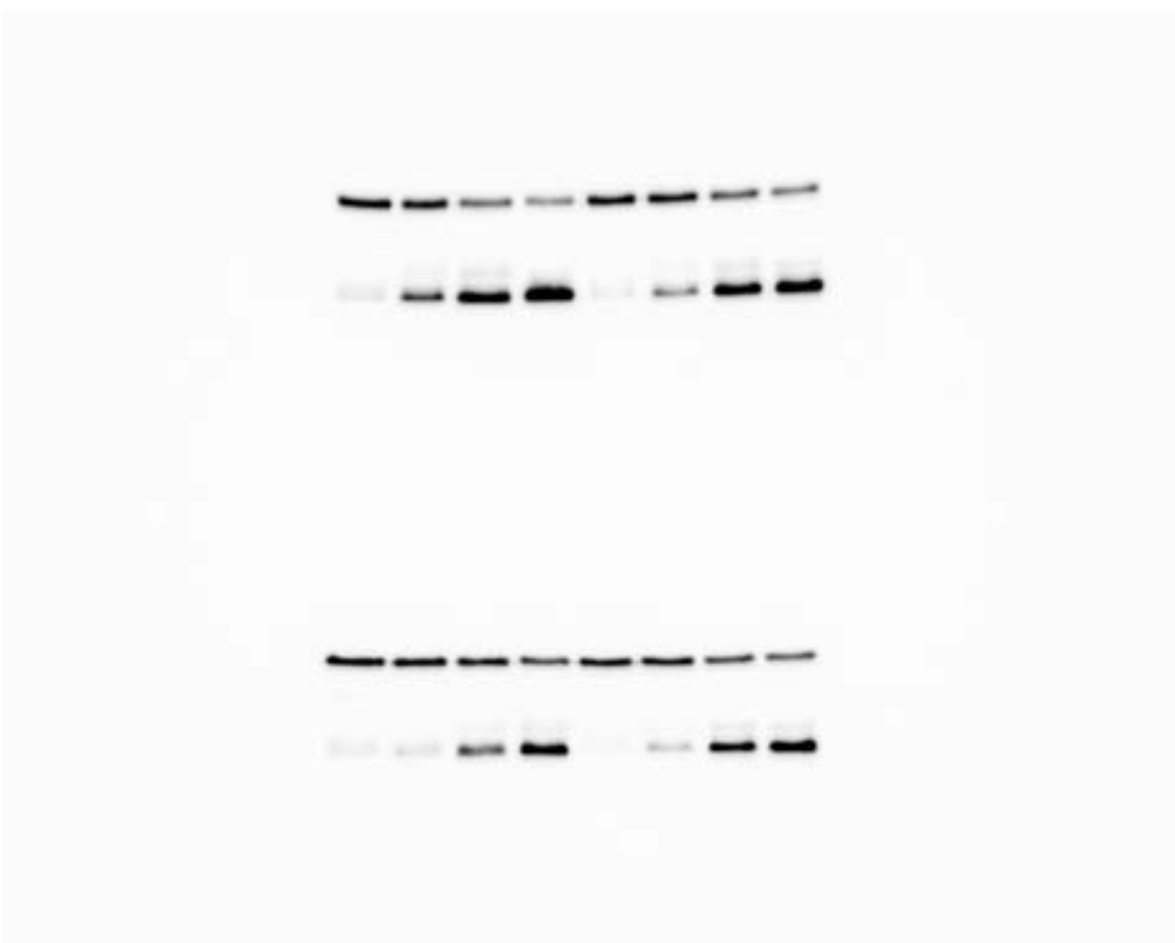

Used areas

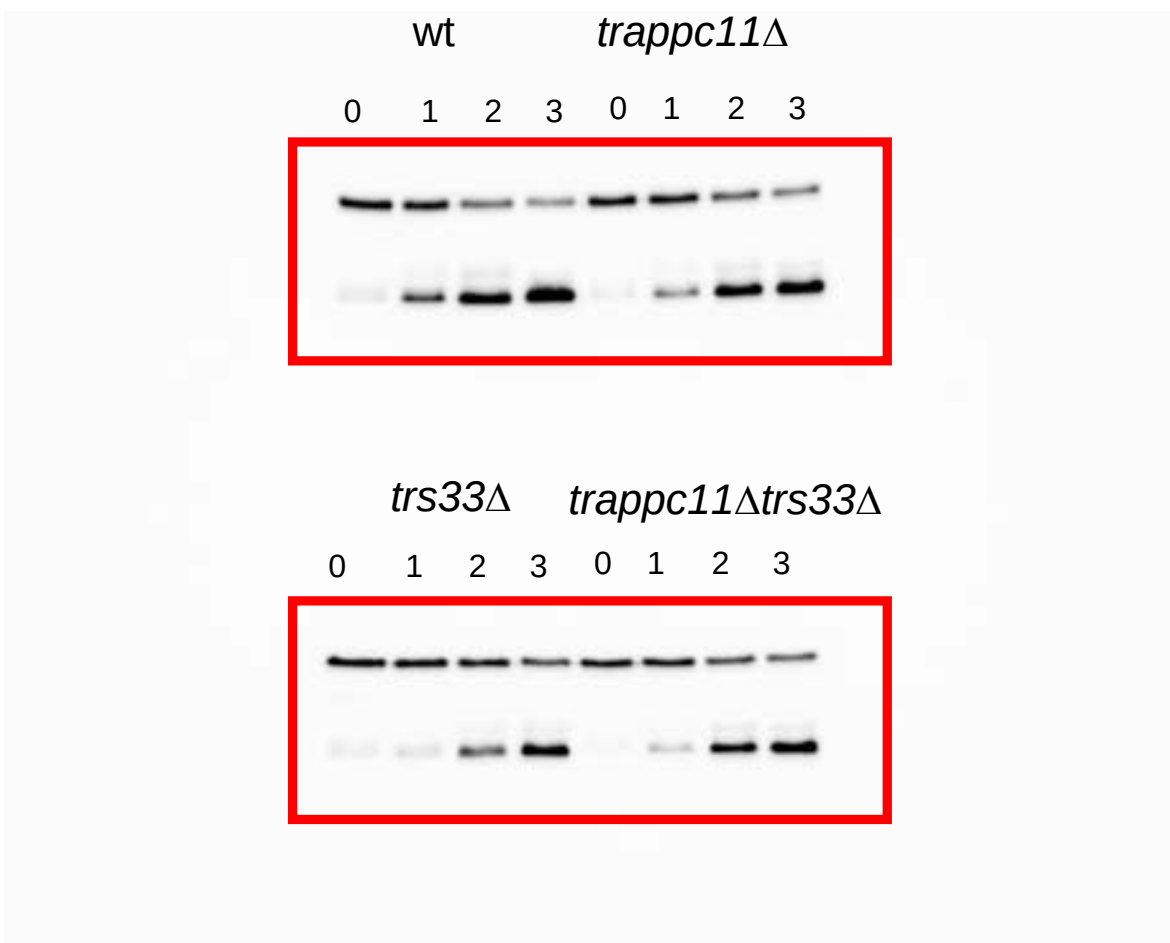

Journal of Cell Science • Supplementary information

Figure 1C-3

α-GFP original images

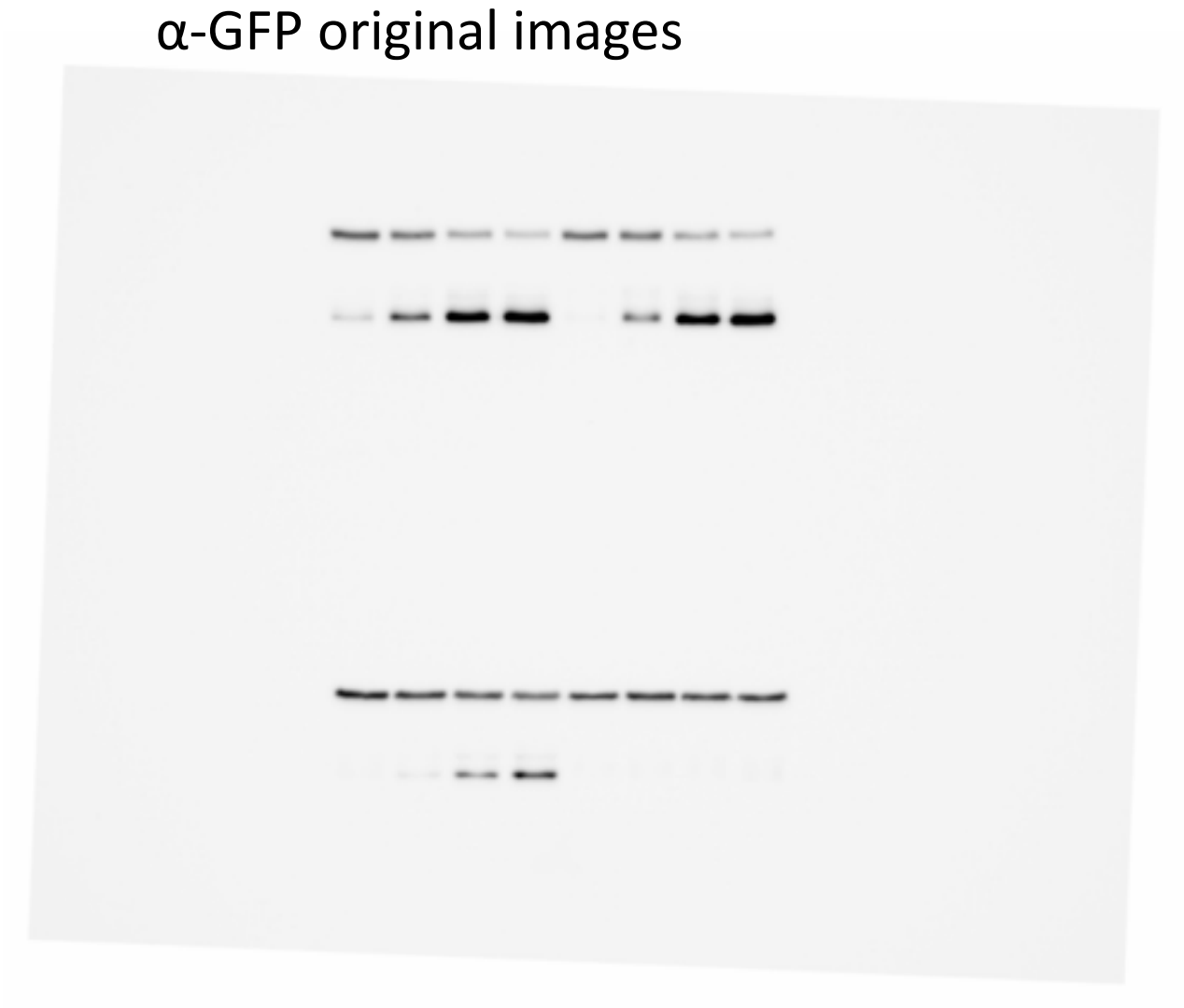

Used areas

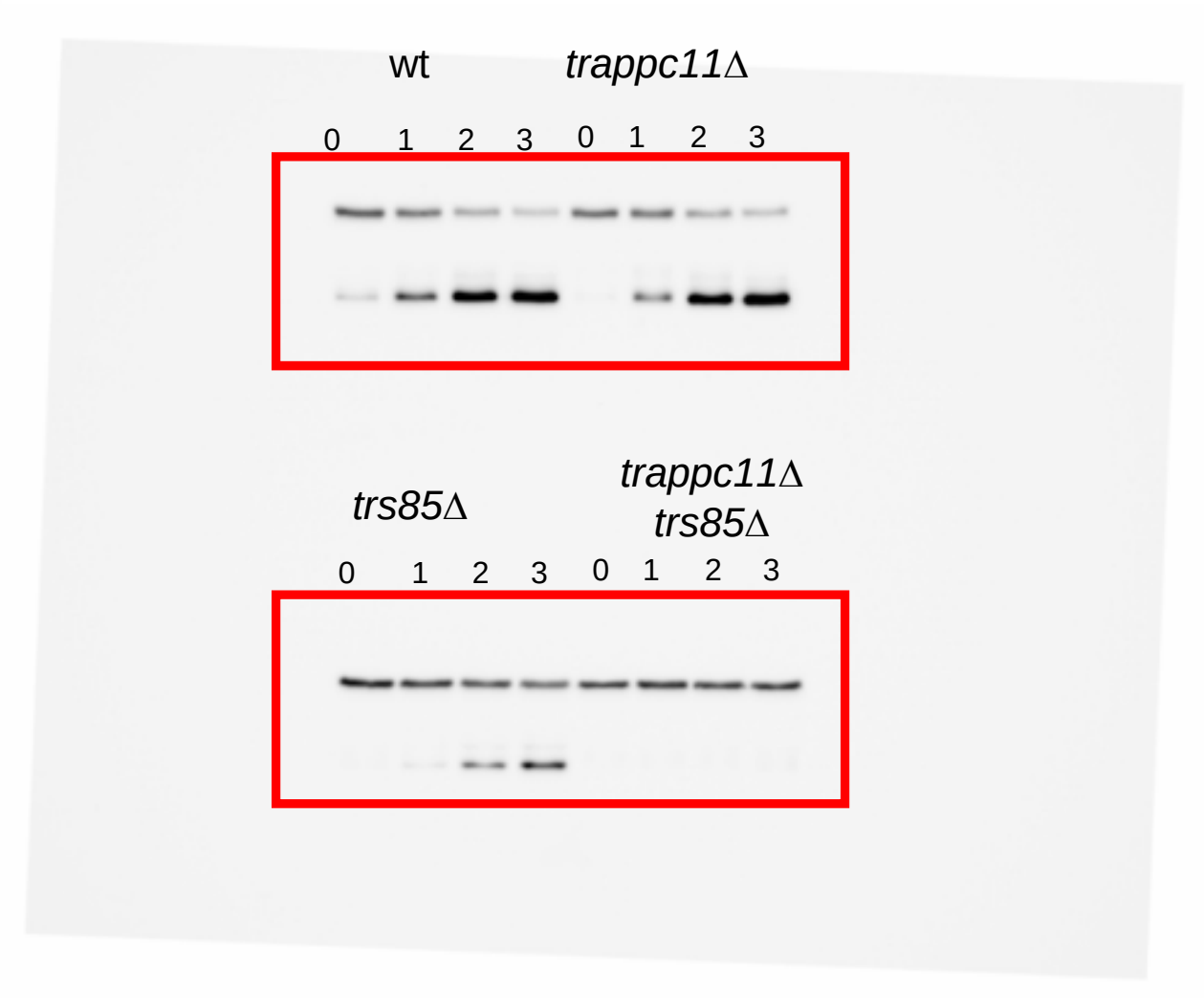

Figure 1C-4

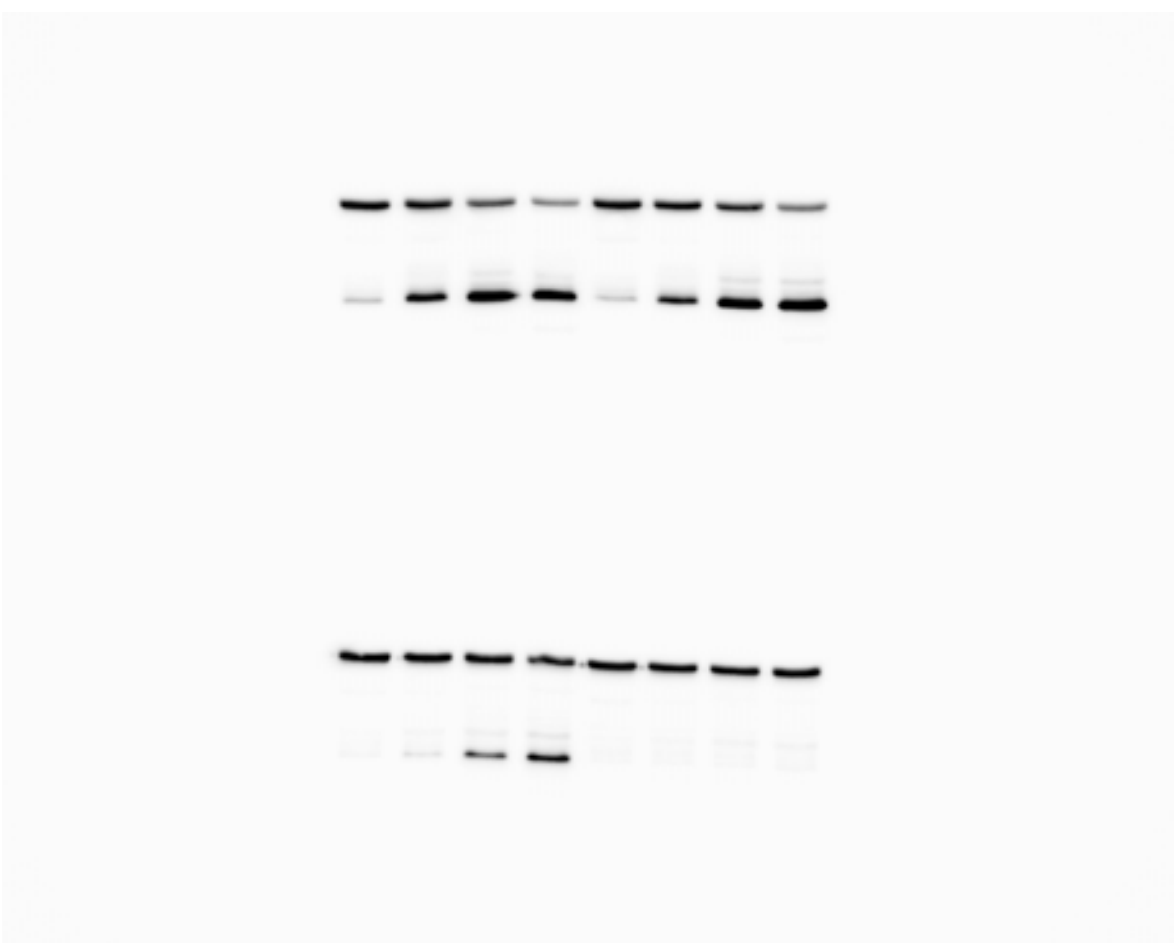

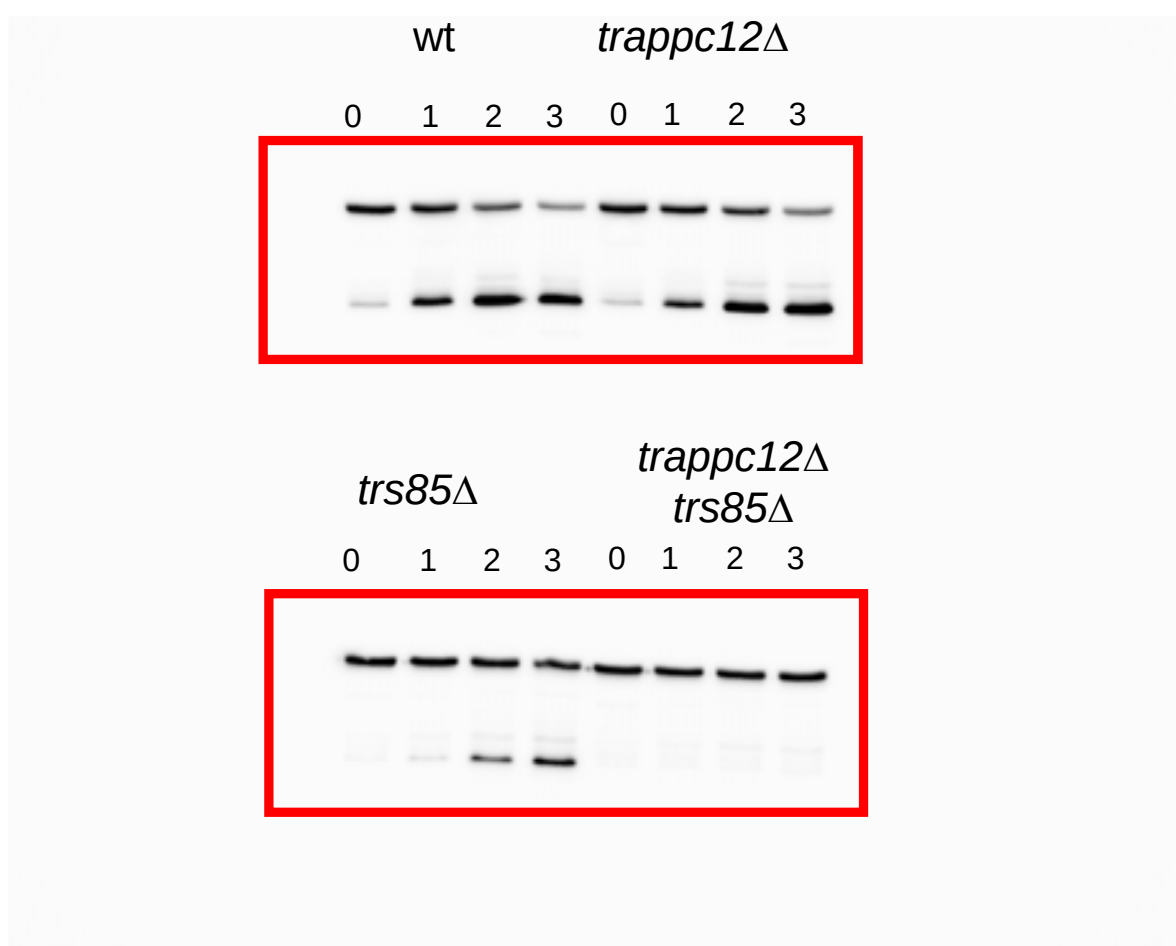

Journal of Cell Science • Supplementary information

Figure 1C-5

α-GFP original images

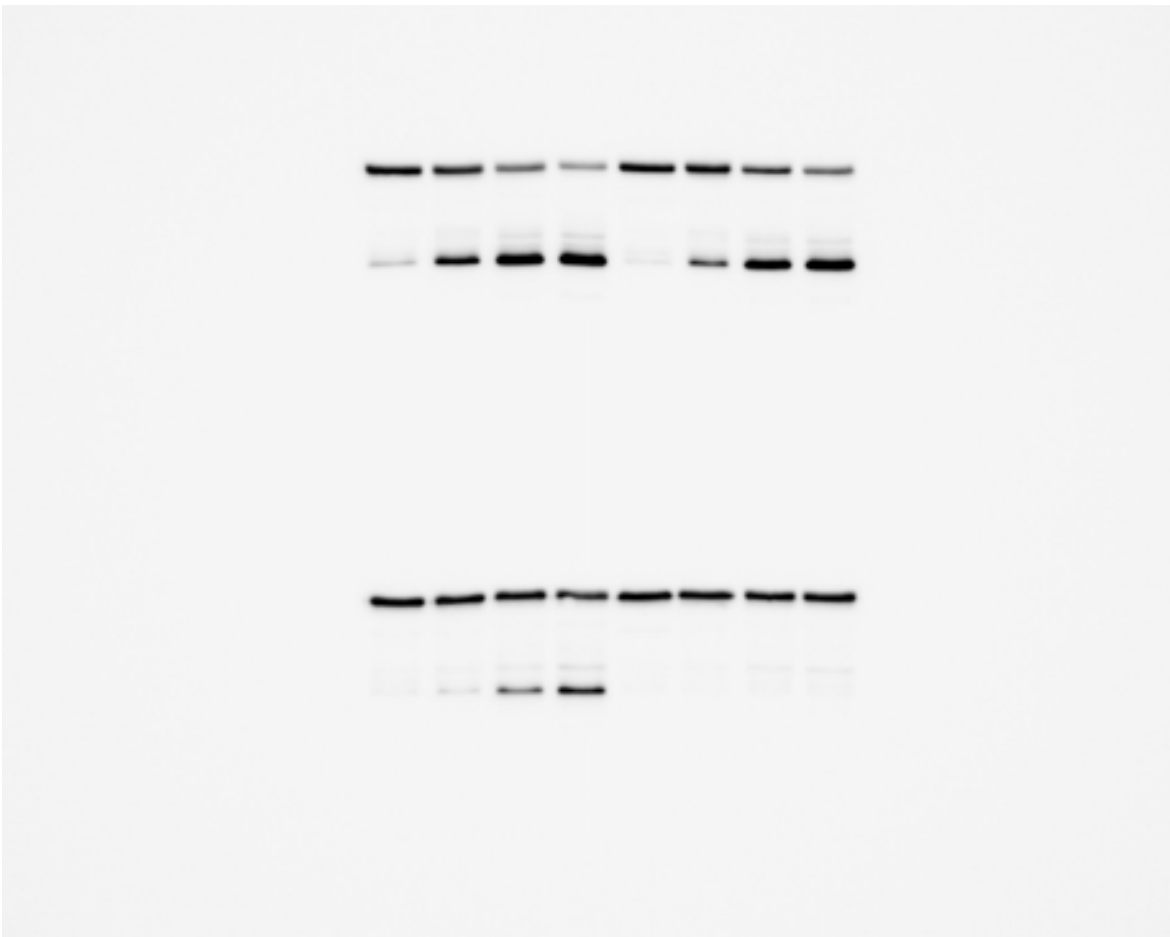

Used areas

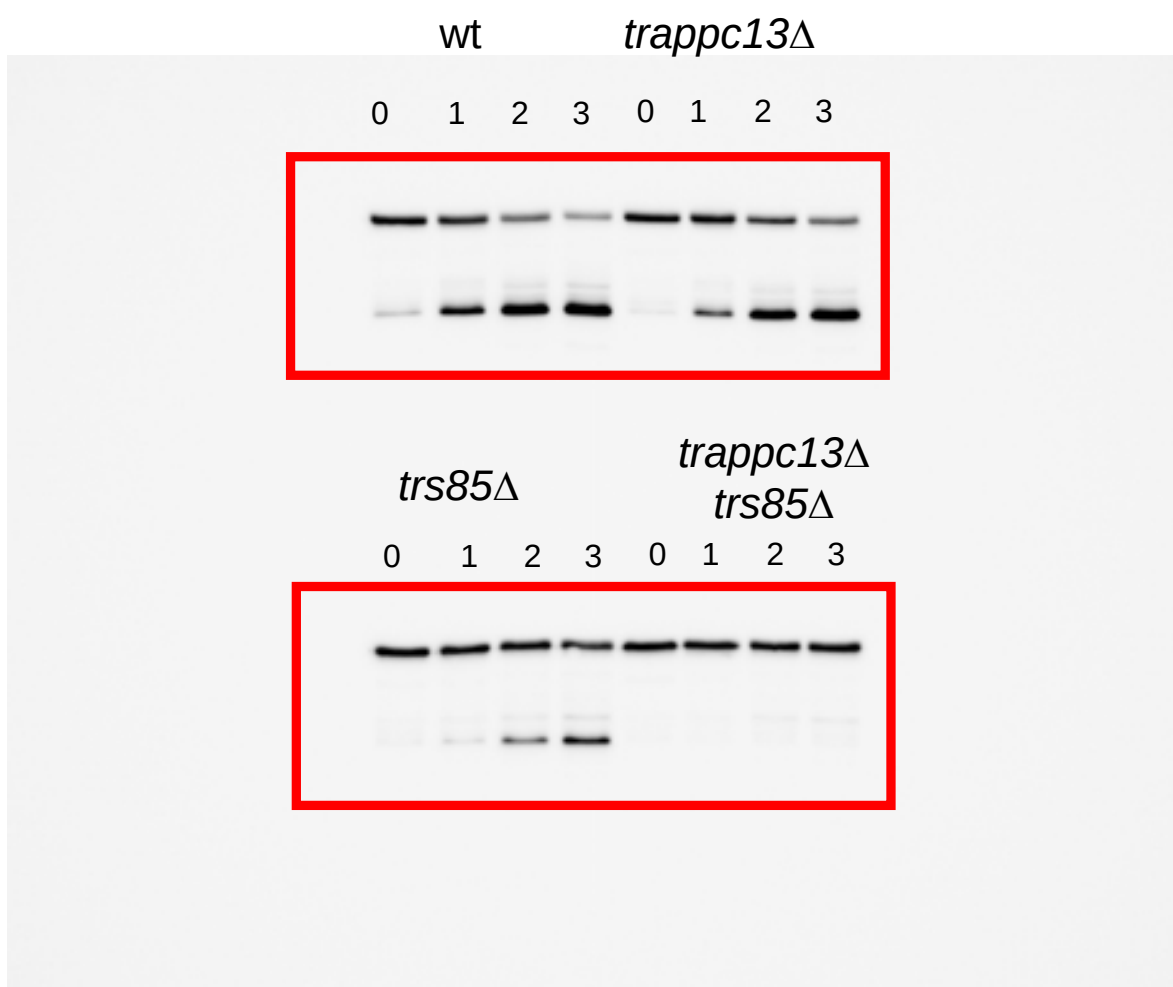

Figure 1D-Source data

α-GFP original images

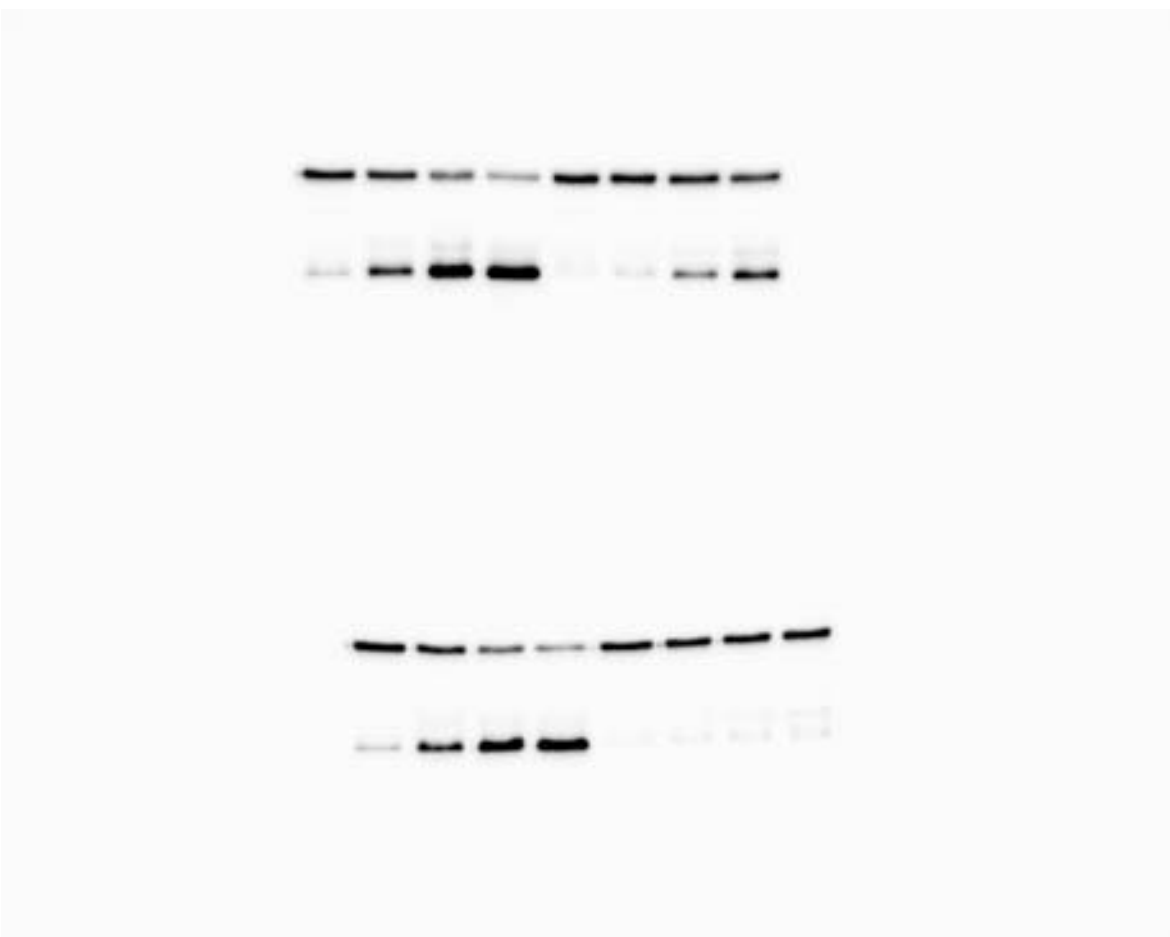

Used areas

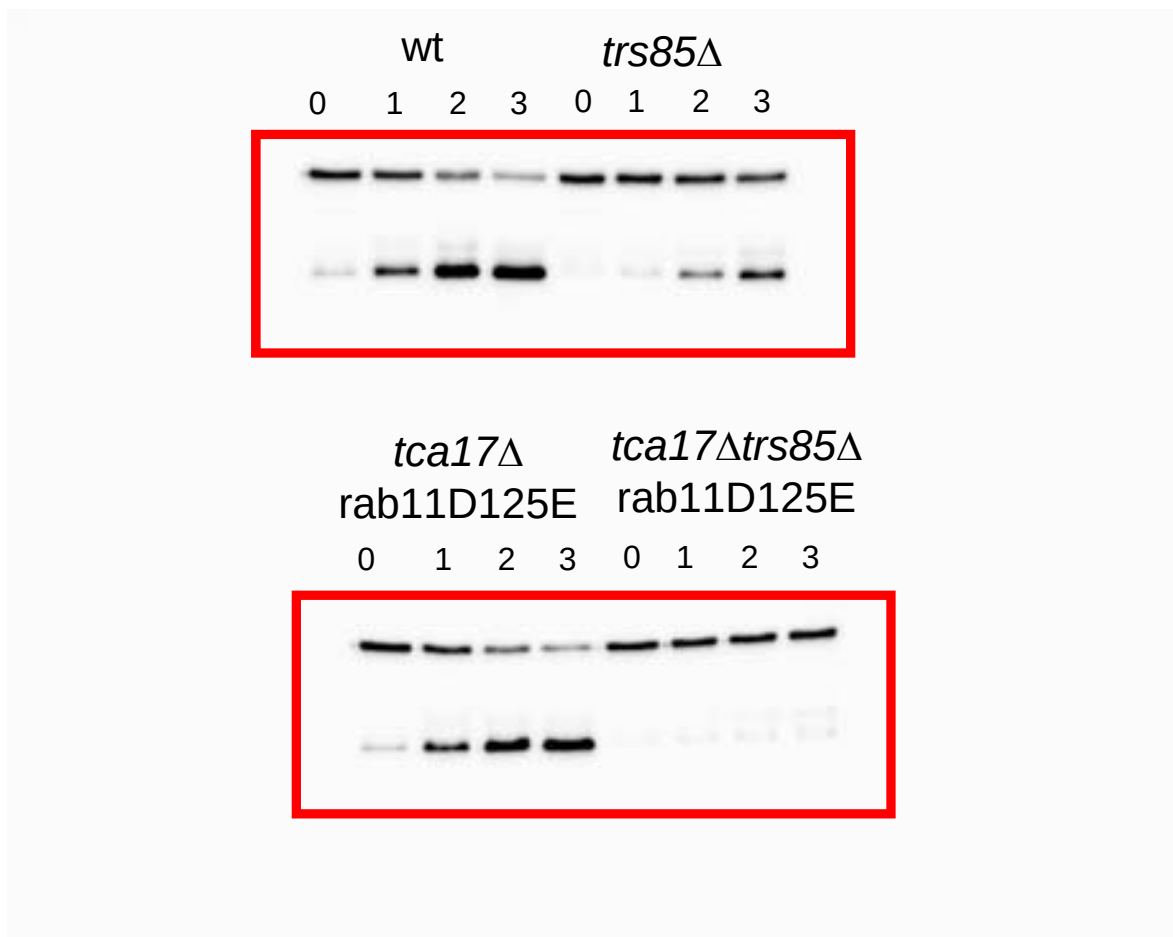

Journal of Cell Science • Supplementary information

Figure 6-Source data

Figure 6A

α-HA TRAPPC11 original images

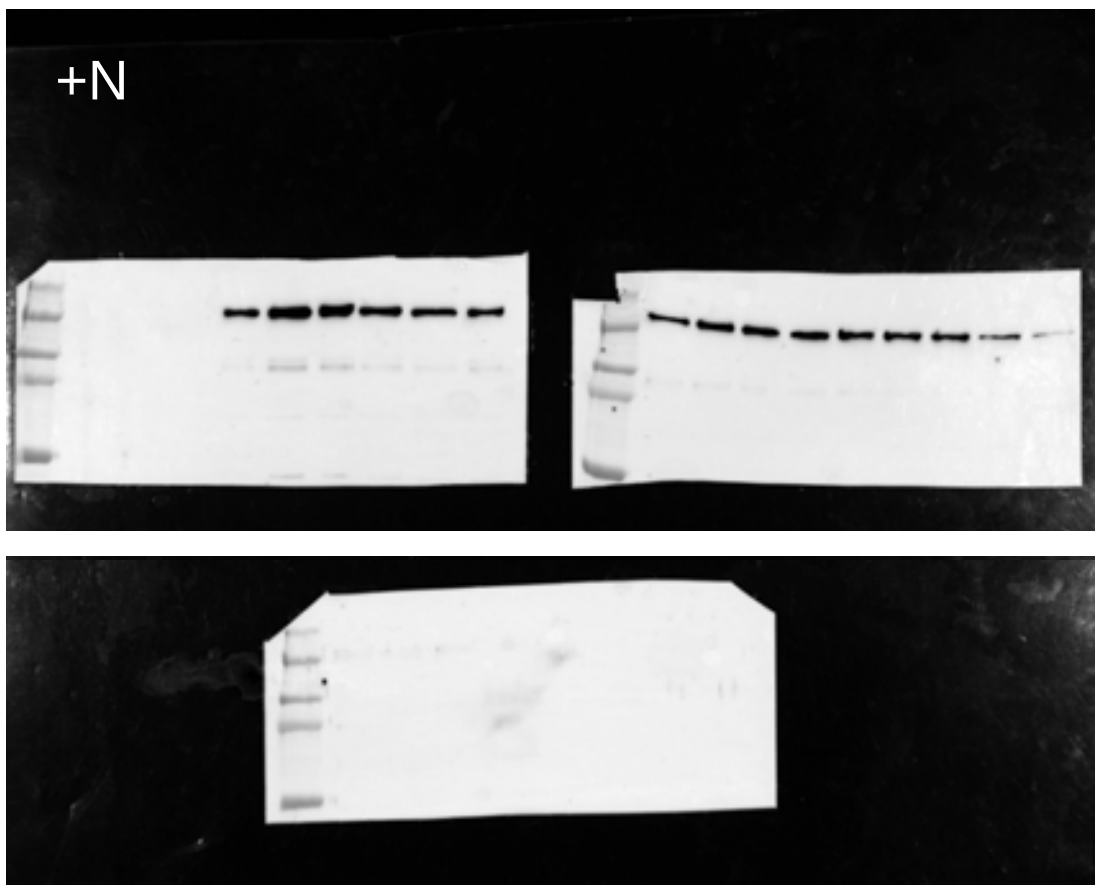

Used areas

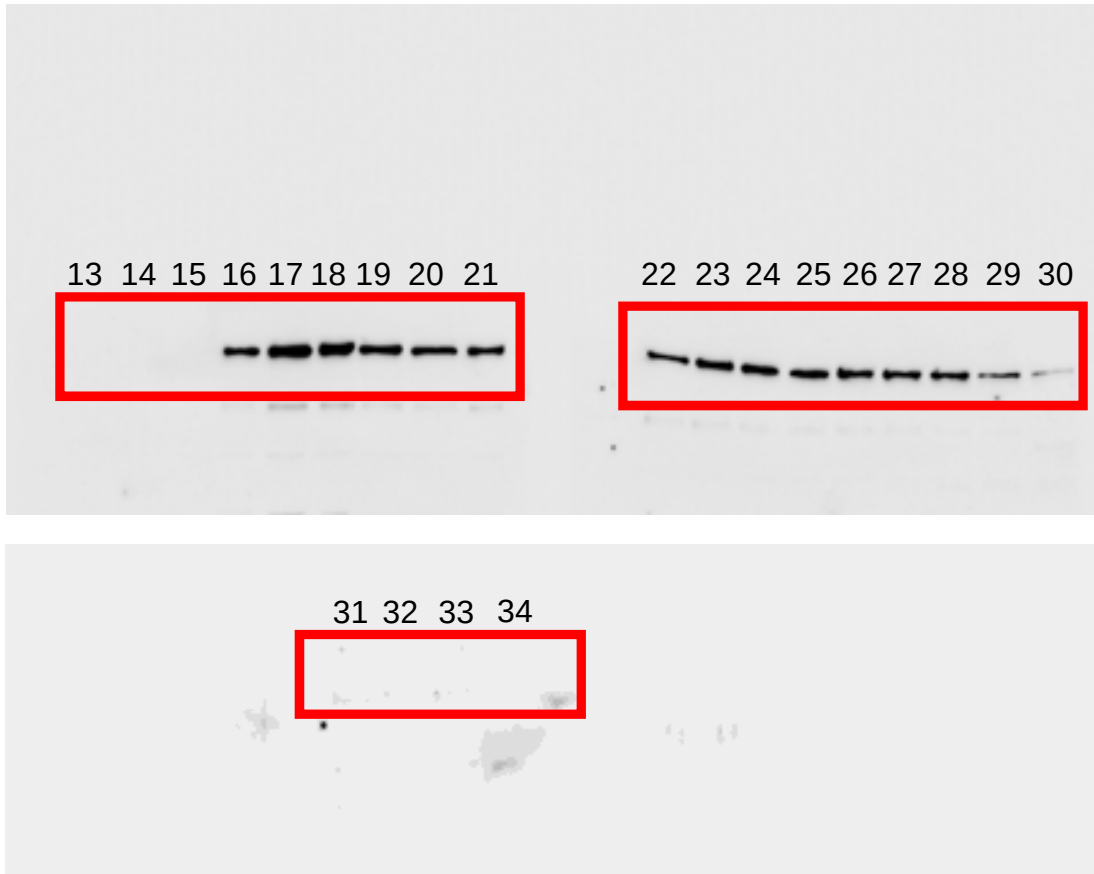

α-HA Trs85 original images

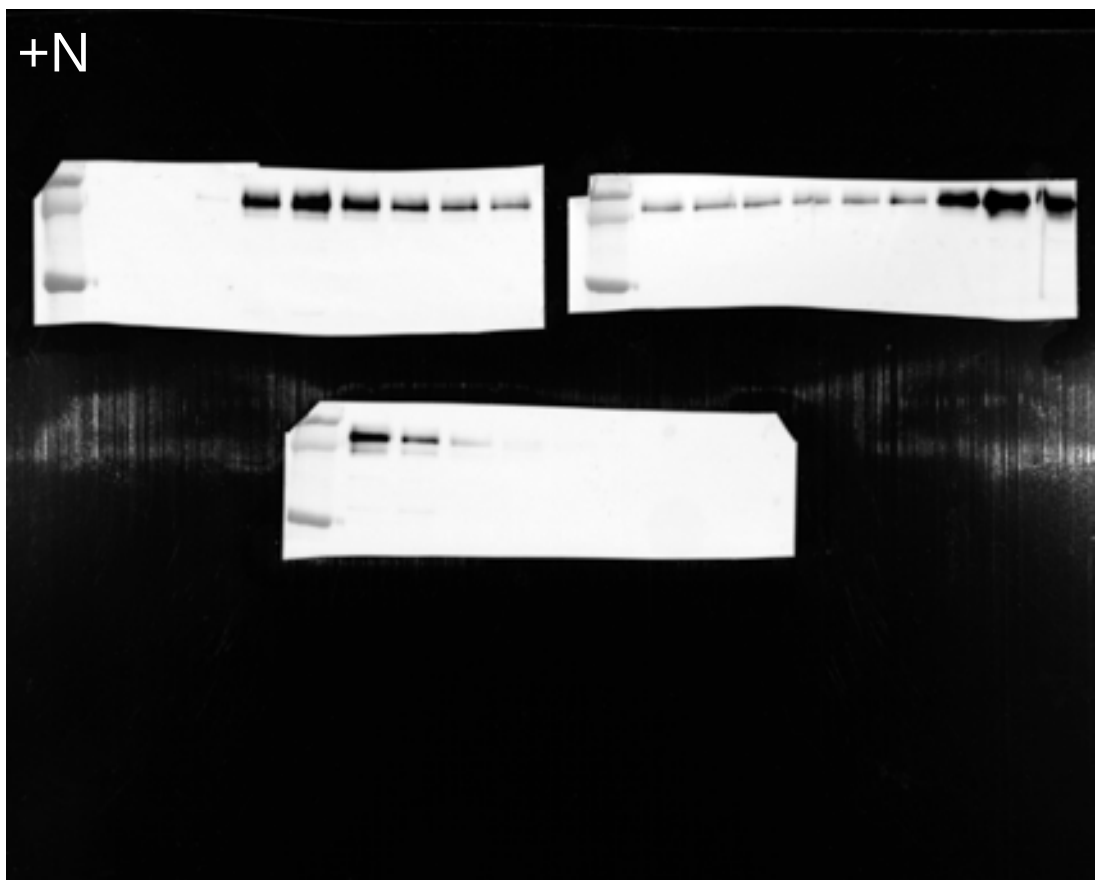

Used areas

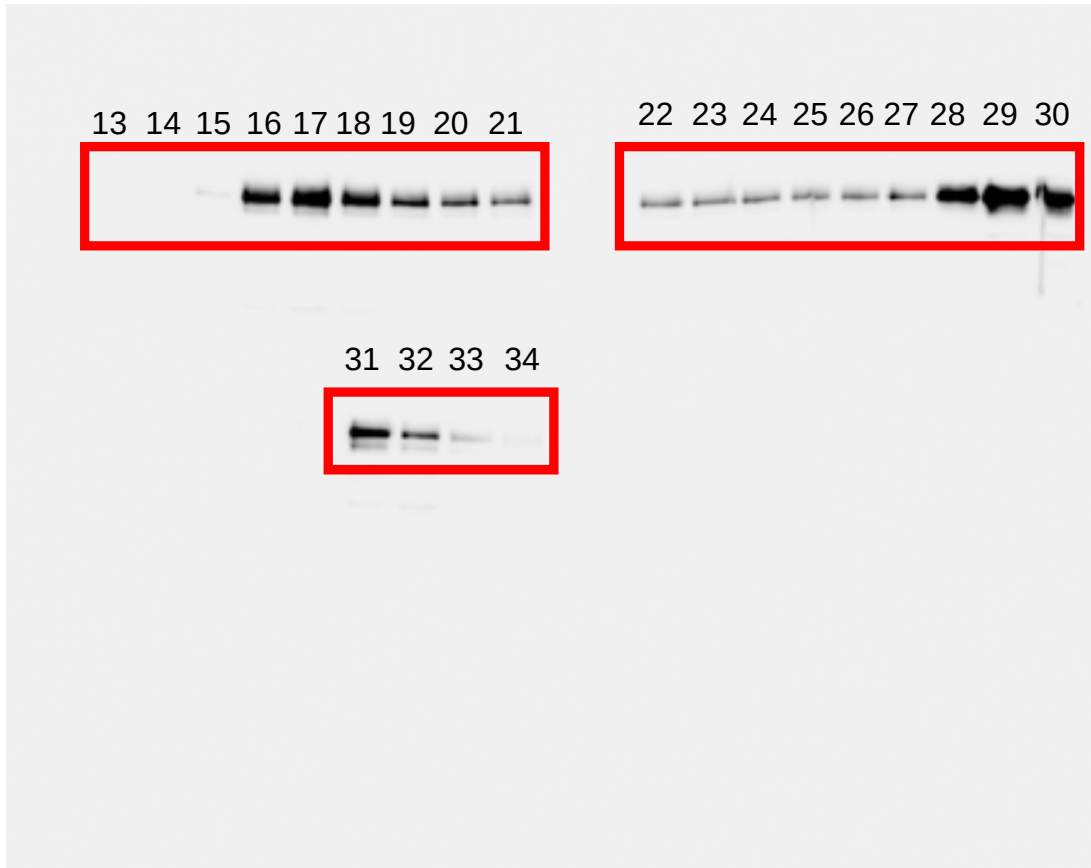

Figure 6B

α-HA TRAPPC11 original images

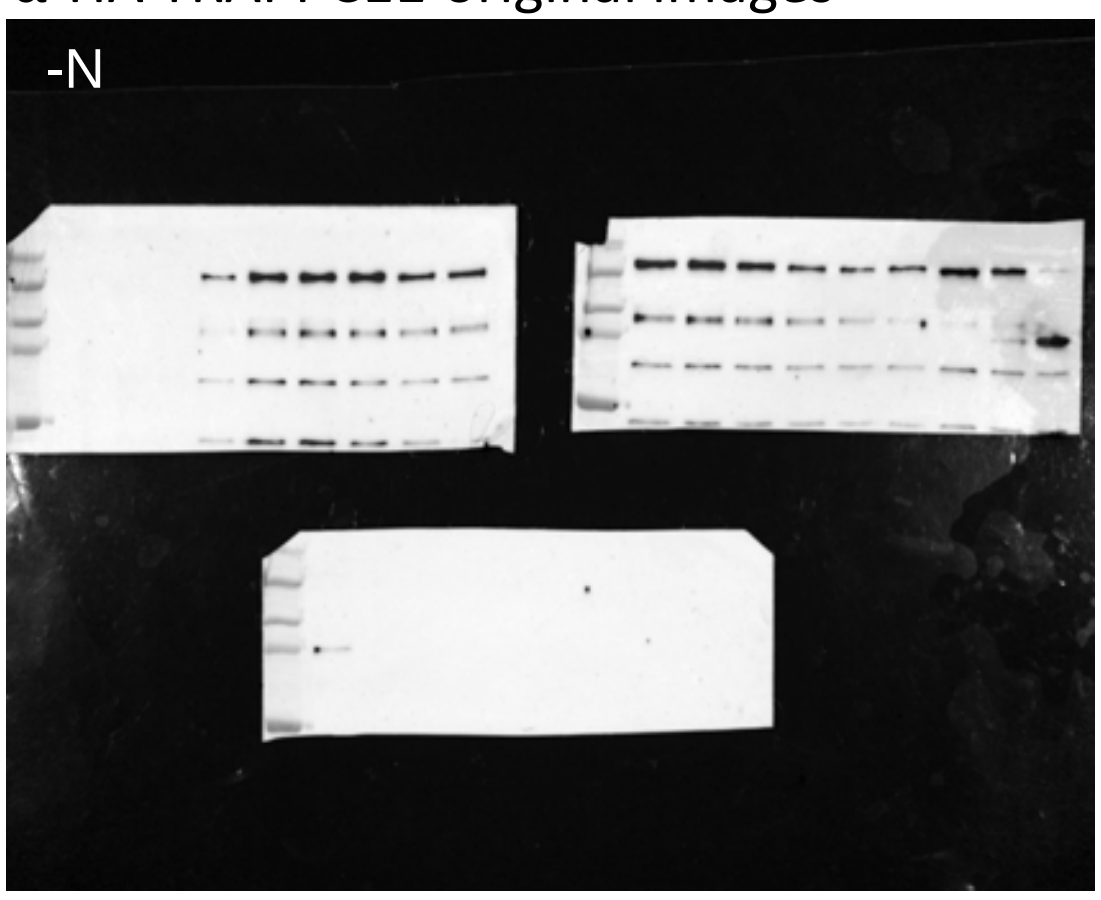

Used areas

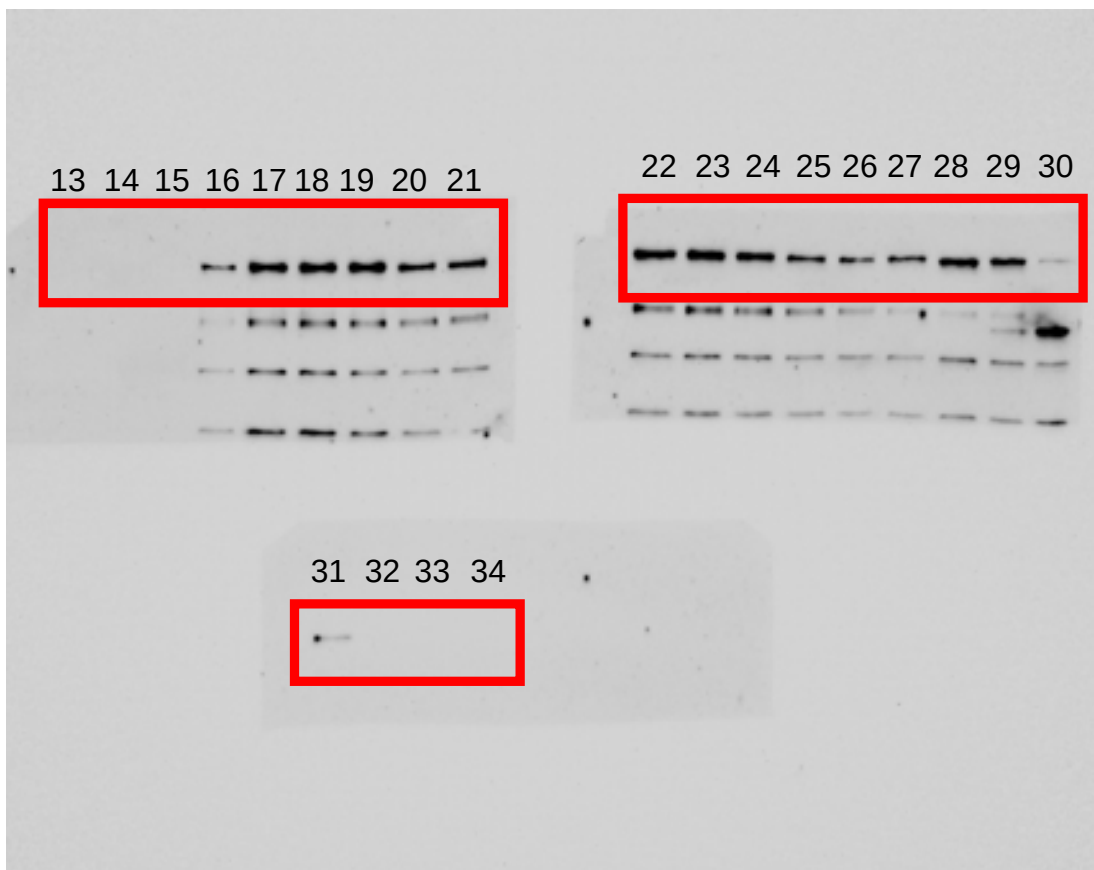

α-HA Trs85 original images

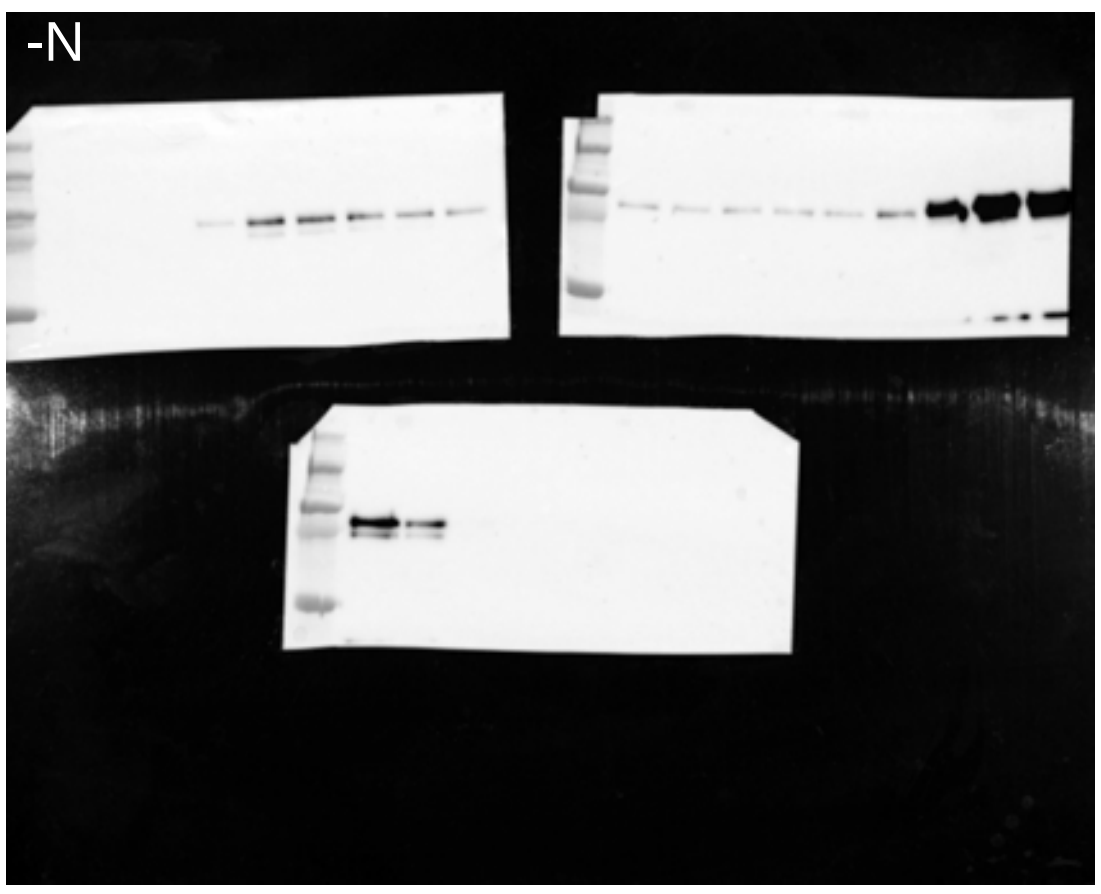

Used areas

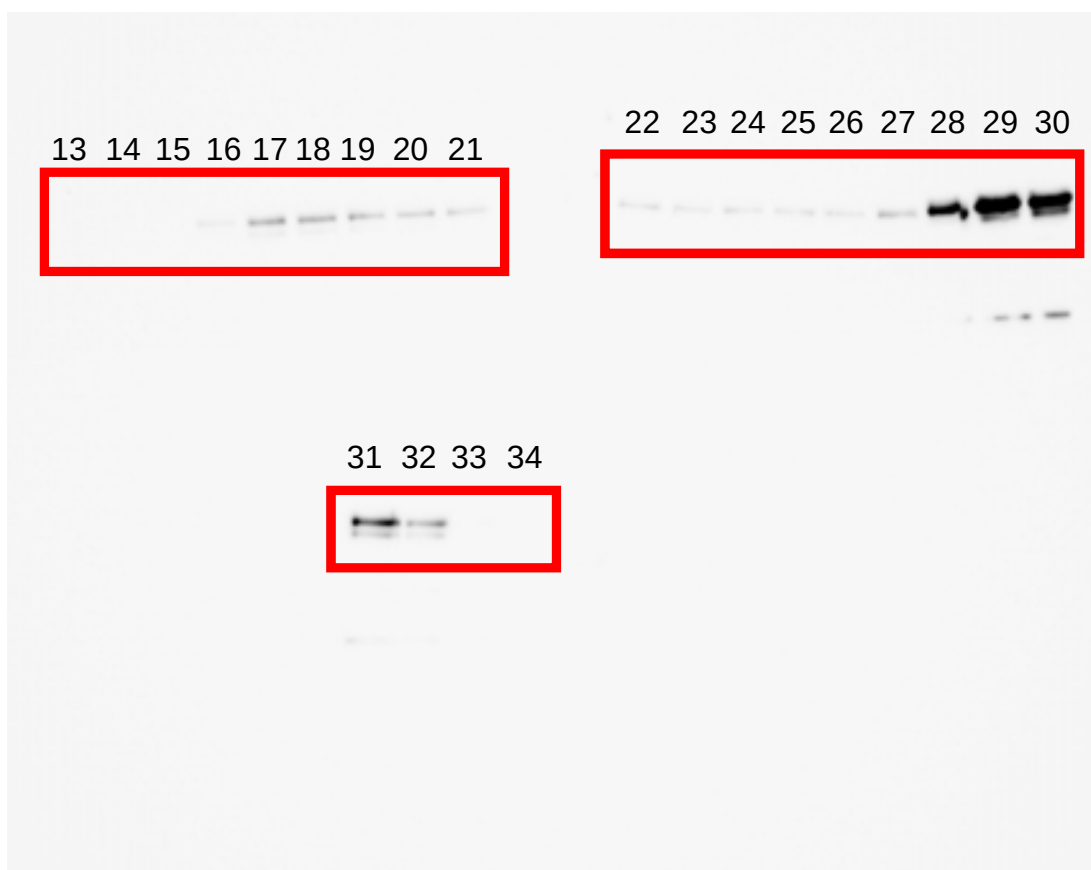

Journal of Cell Science • Supplementary information

Figure 6C

α-HA TRAPPC11 original images

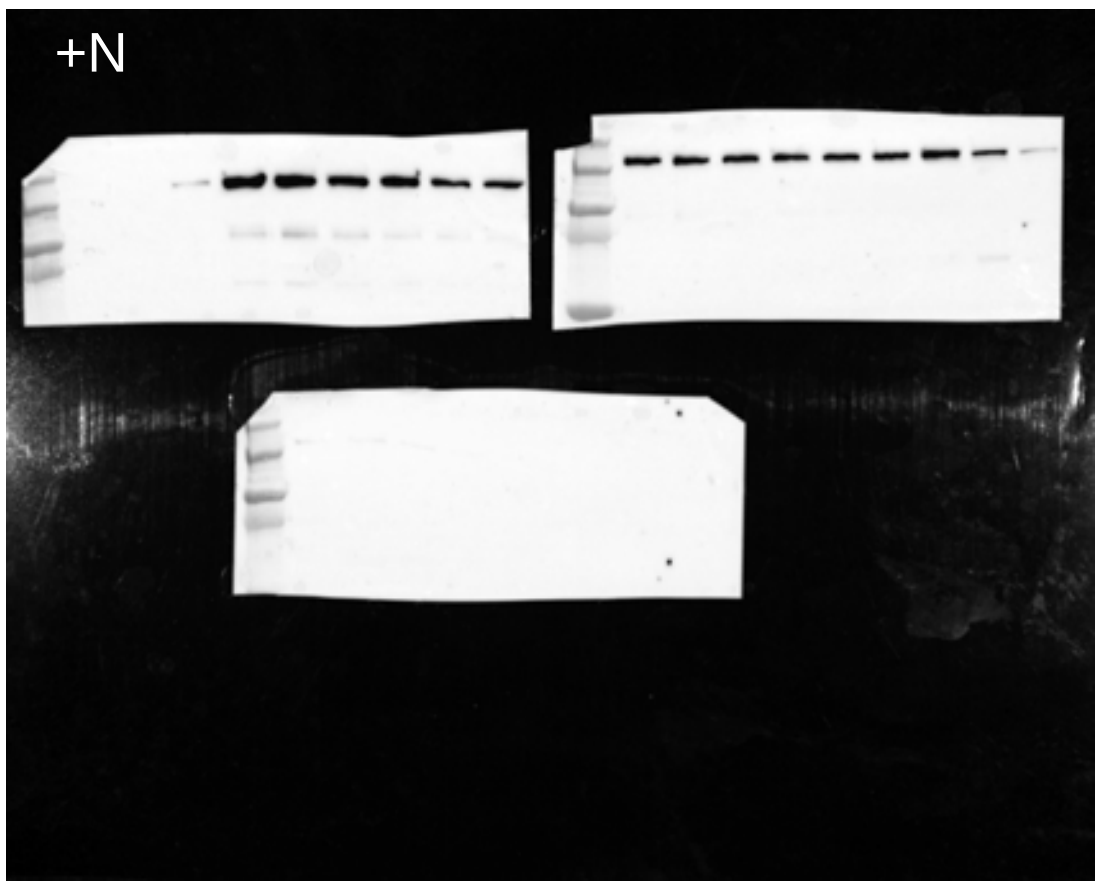

Used areas

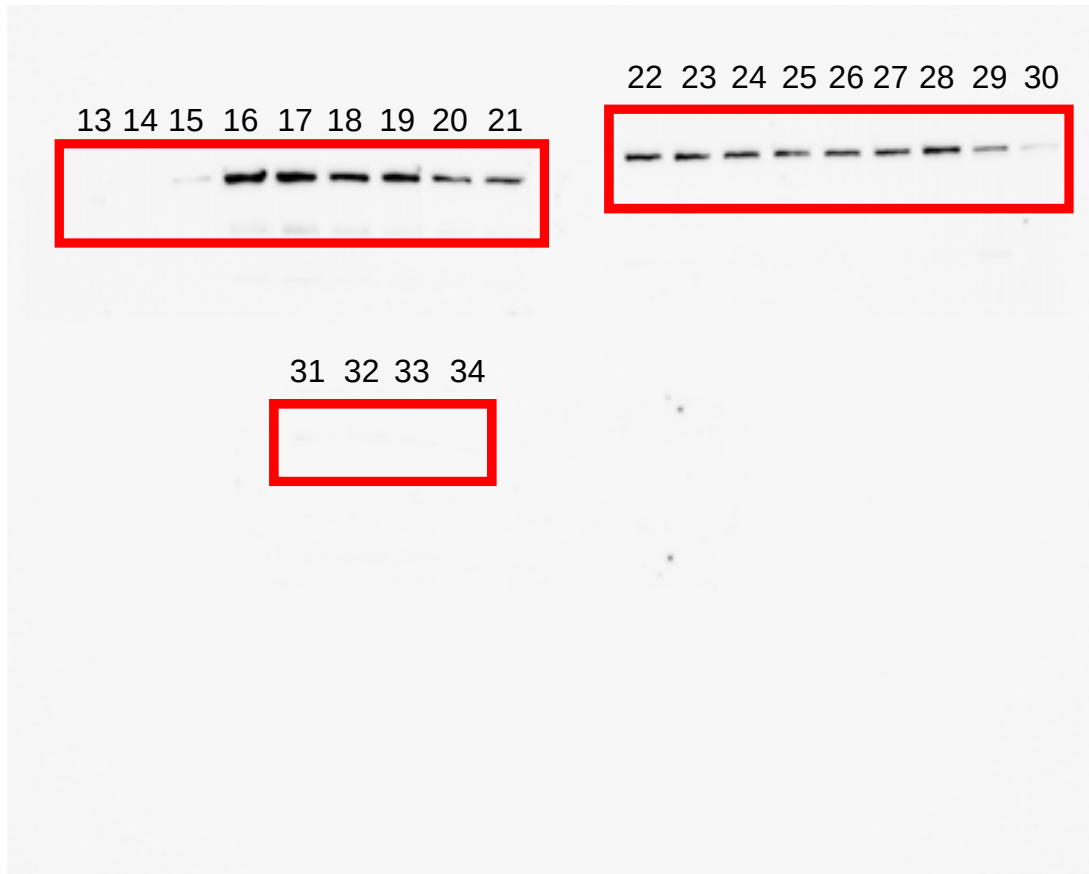

α-HA Trs23 original images

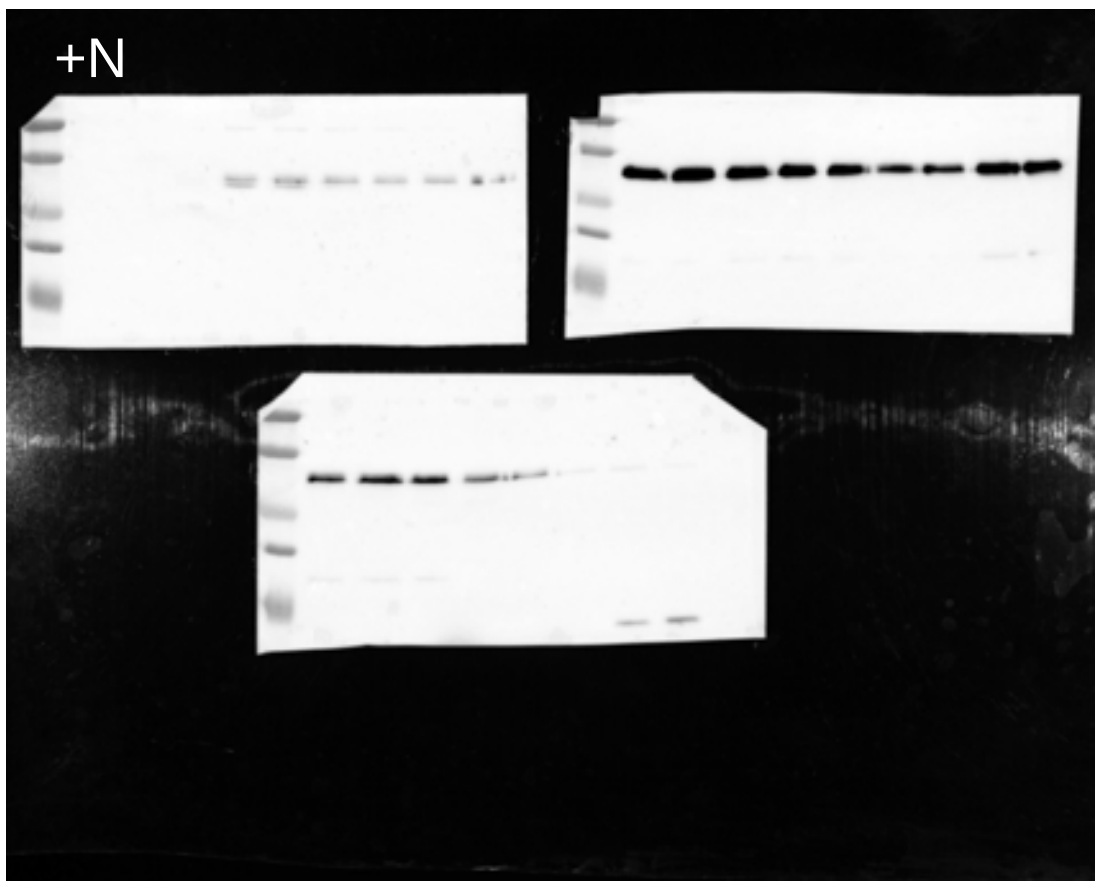

Used areas

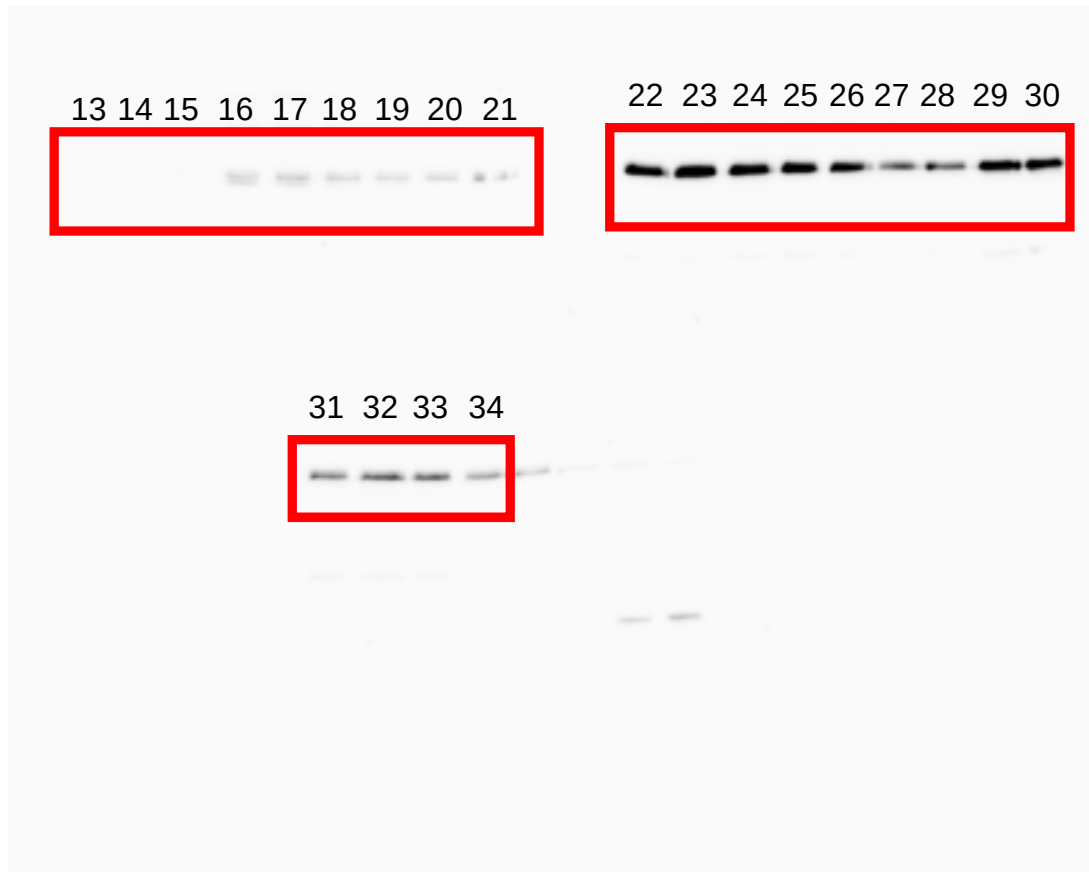

Figure 6D

α-HA TRAPPC11 original images

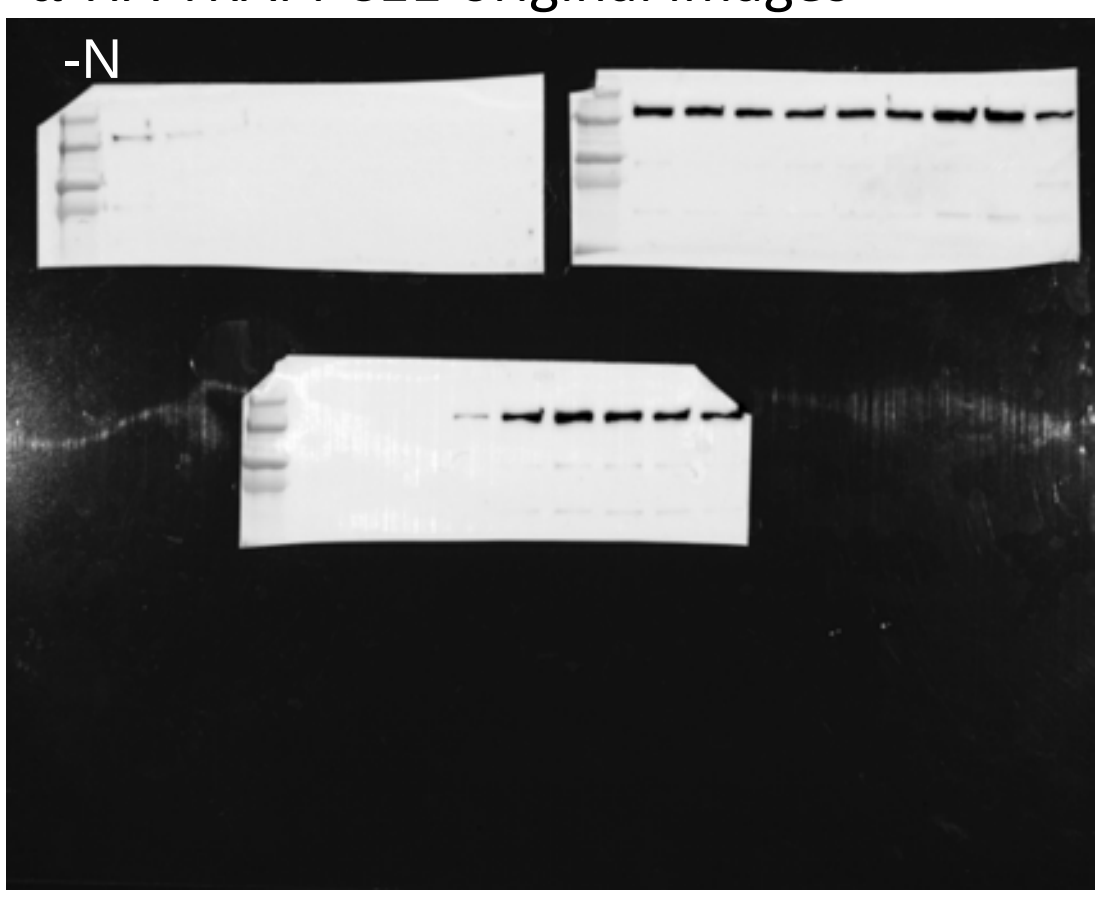

Used areas

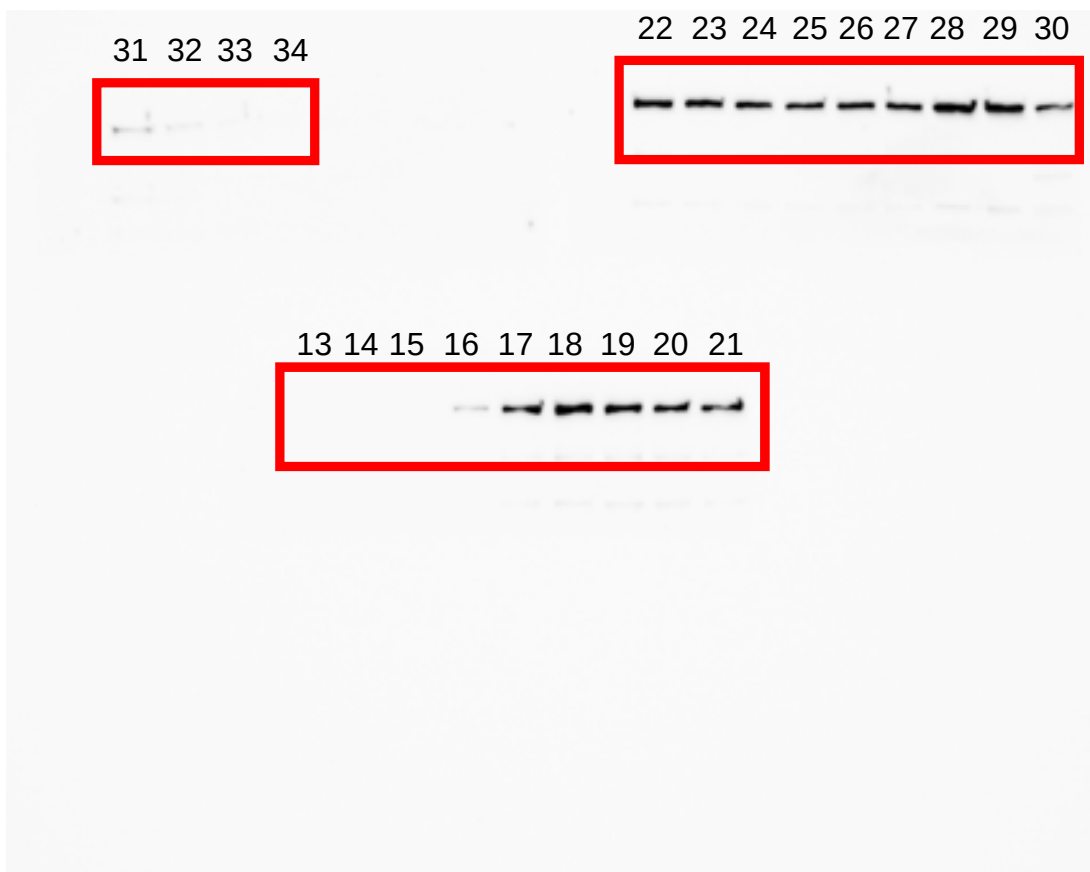

α-HA Trs23 original images

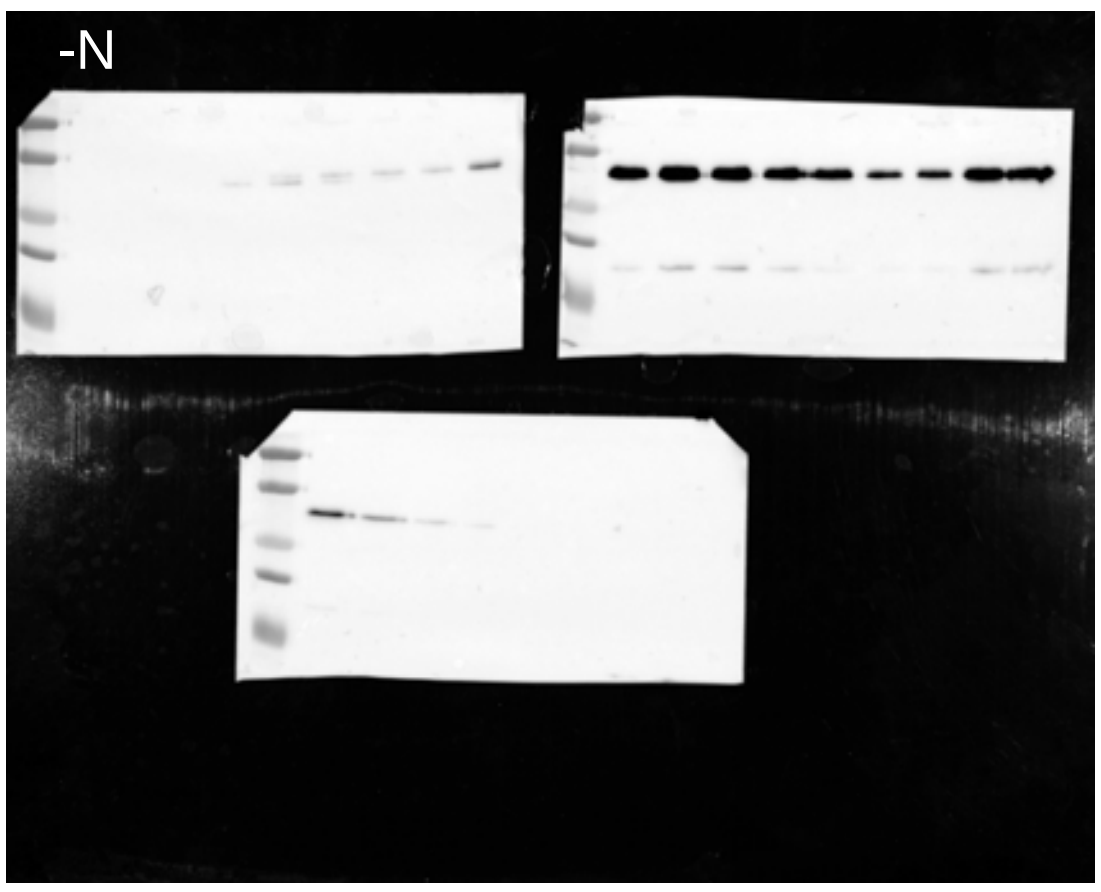

Used areas

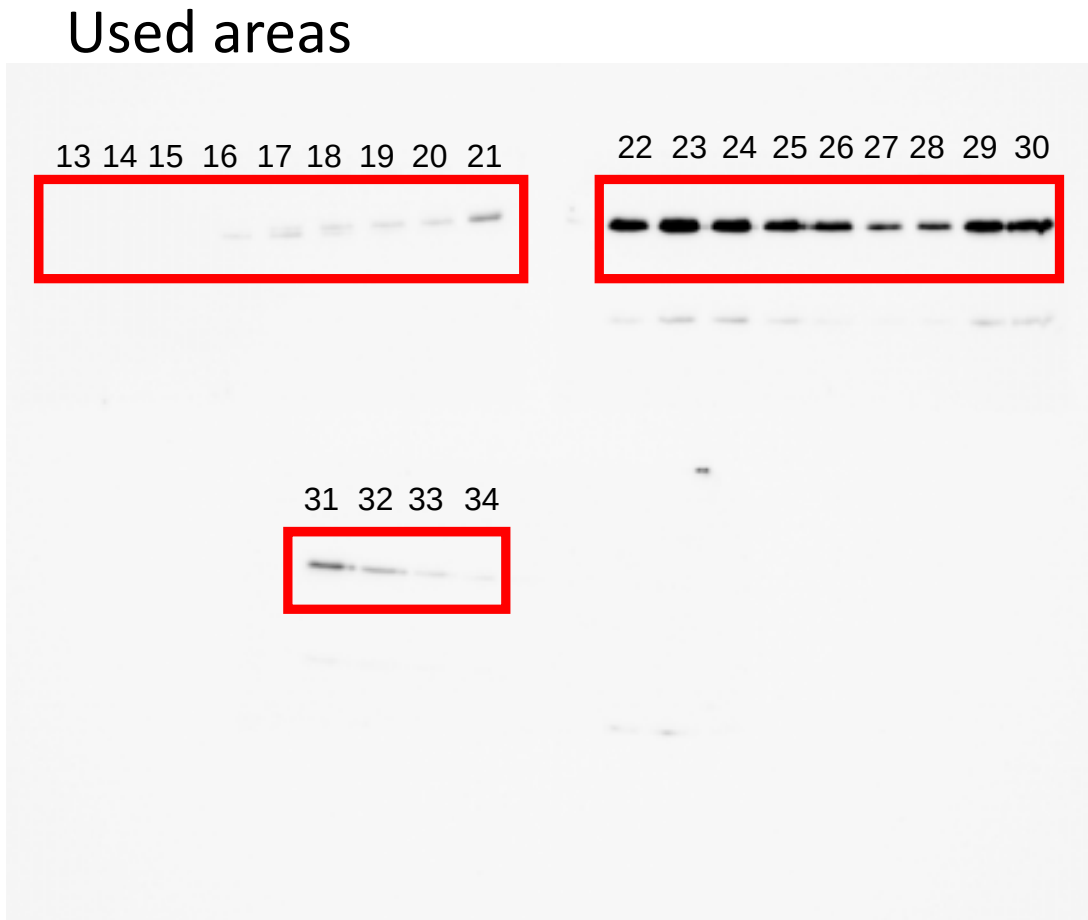

Journal of Cell Science • Supplementary information

Figure 6E

α-HA TRAPPC11 wt +N original images

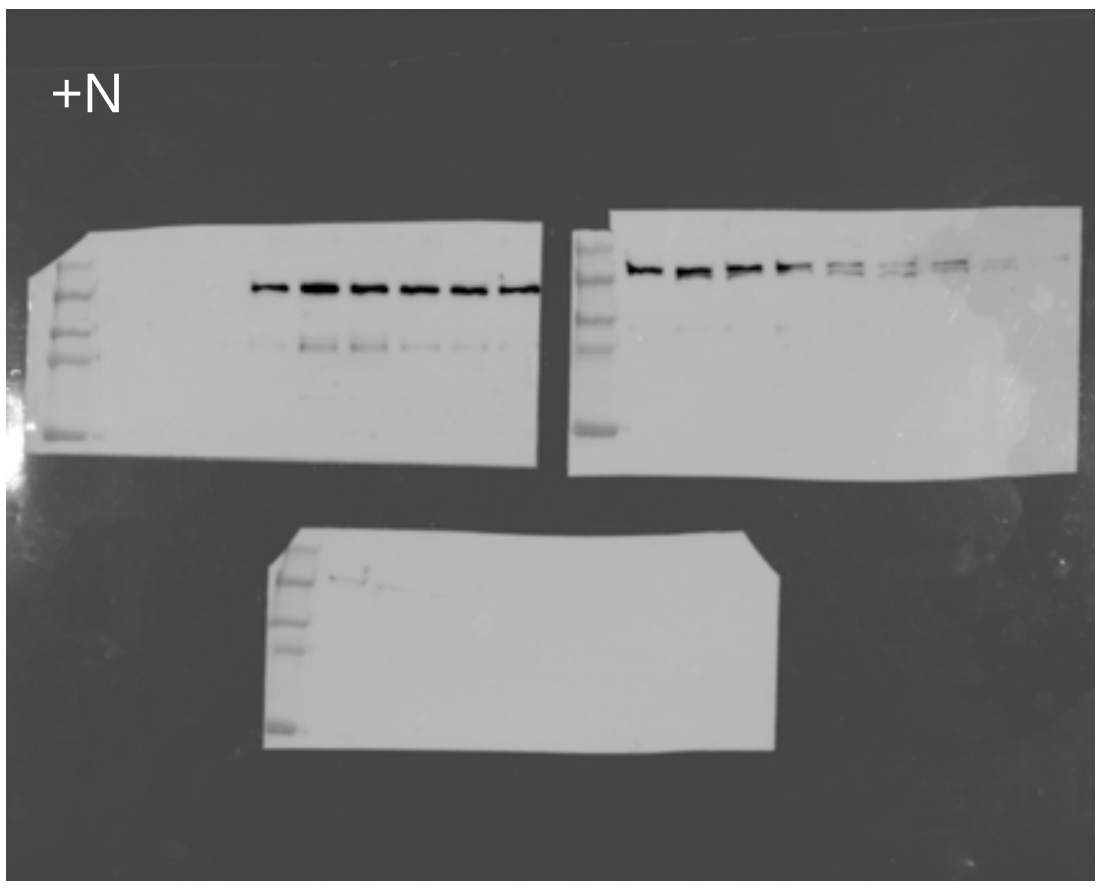

Used areas

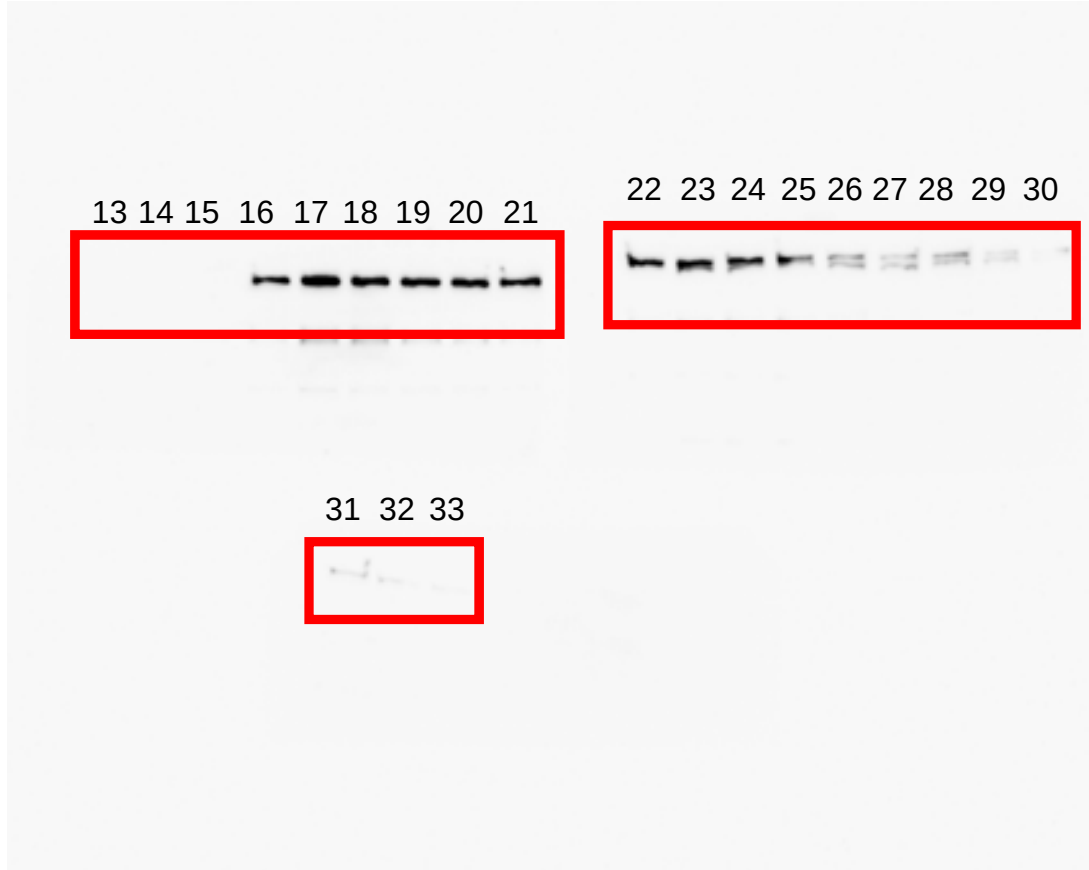

α-HA TRAPPC11 *trs33*Δ +N original images

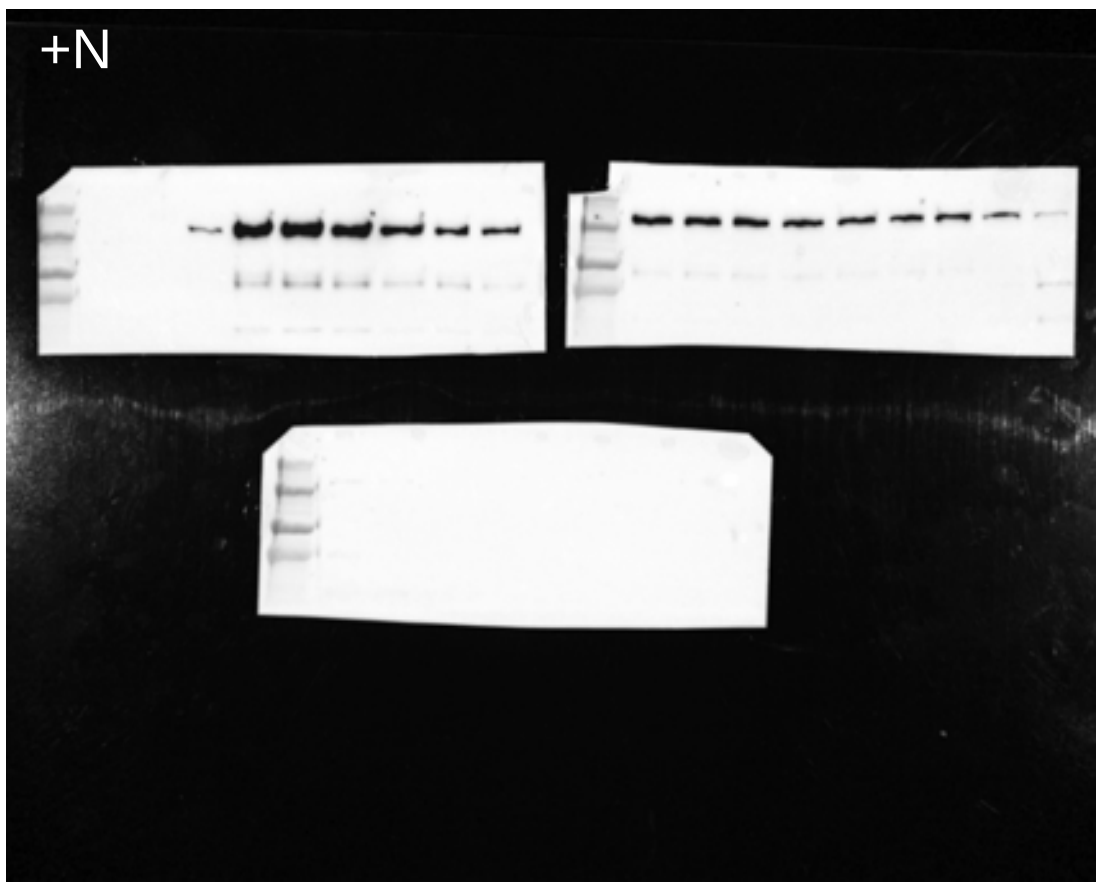

Used areas

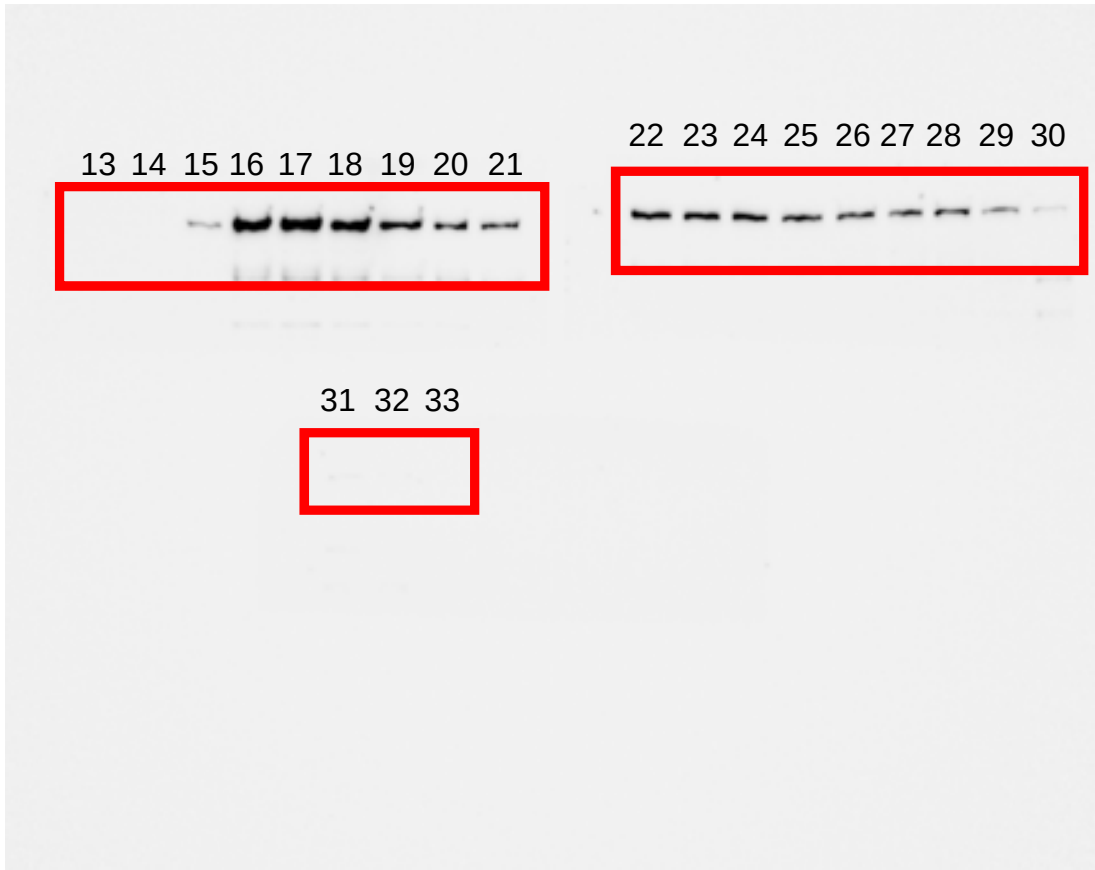

Figure 6F

α-HA TRAPPC11 wt -N original images

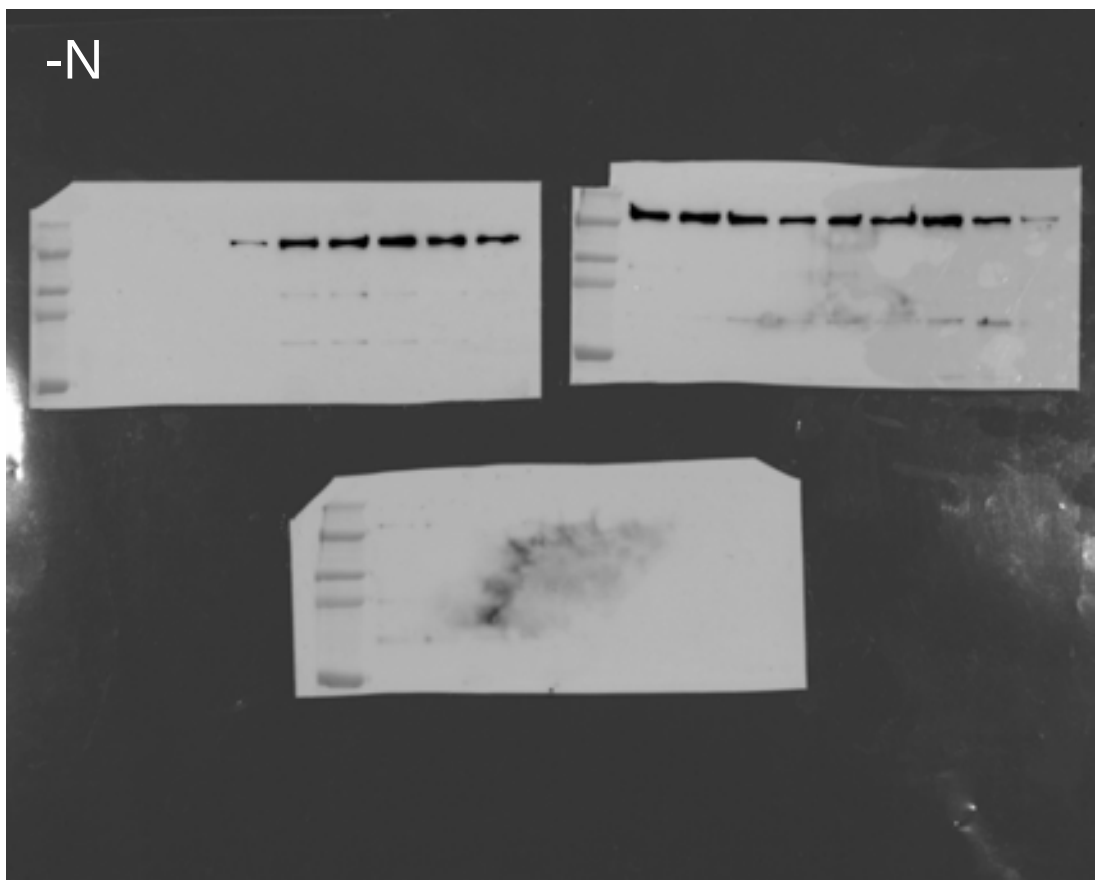

Used areas

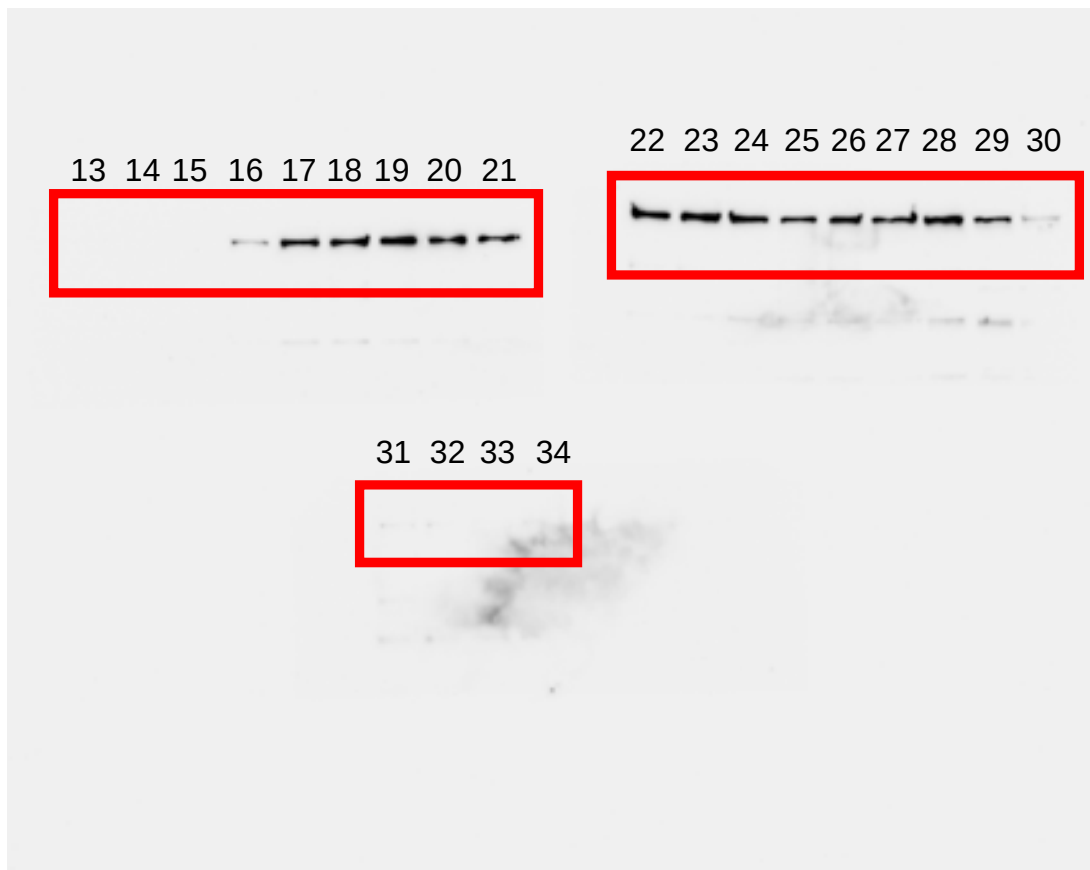

α-HA TRAPPC11 *trs33*Δ -N original images

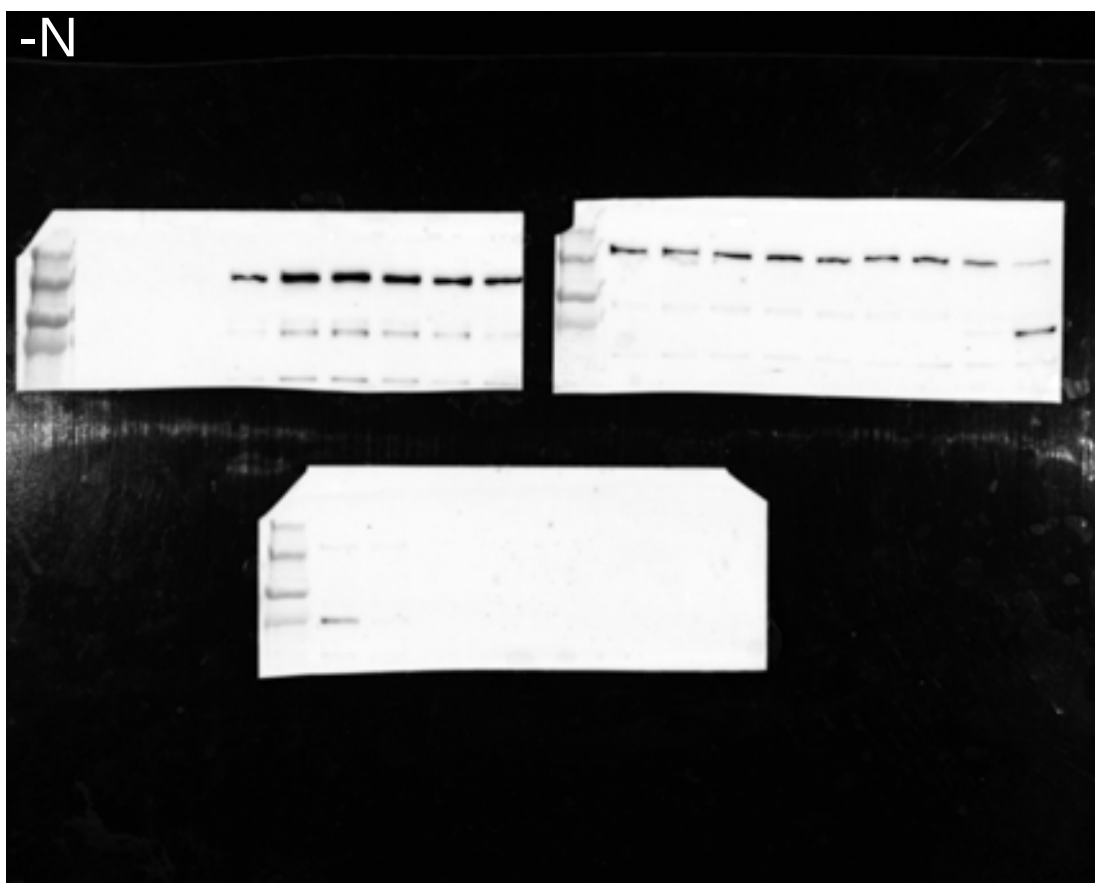

Used areas

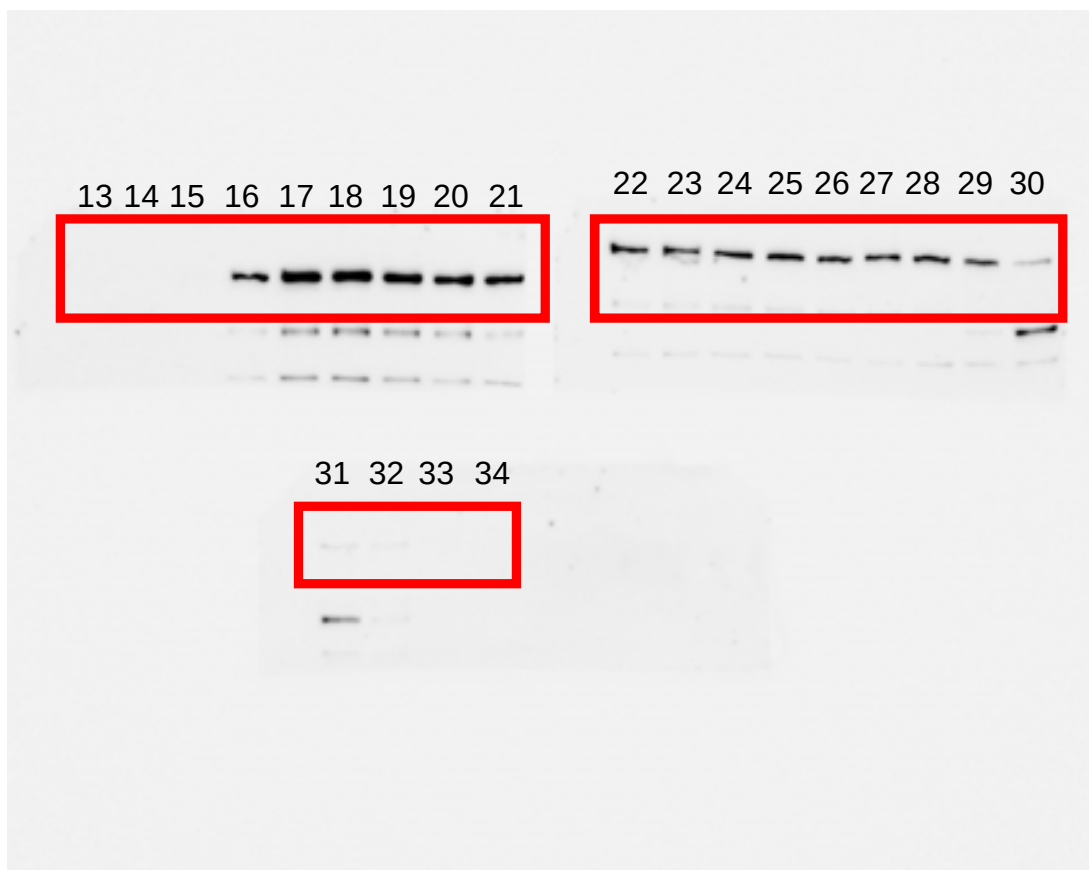

Journal of Cell Science • Supplementary information

Figure 6G

α-HA TRAPPC11 wt +N original images

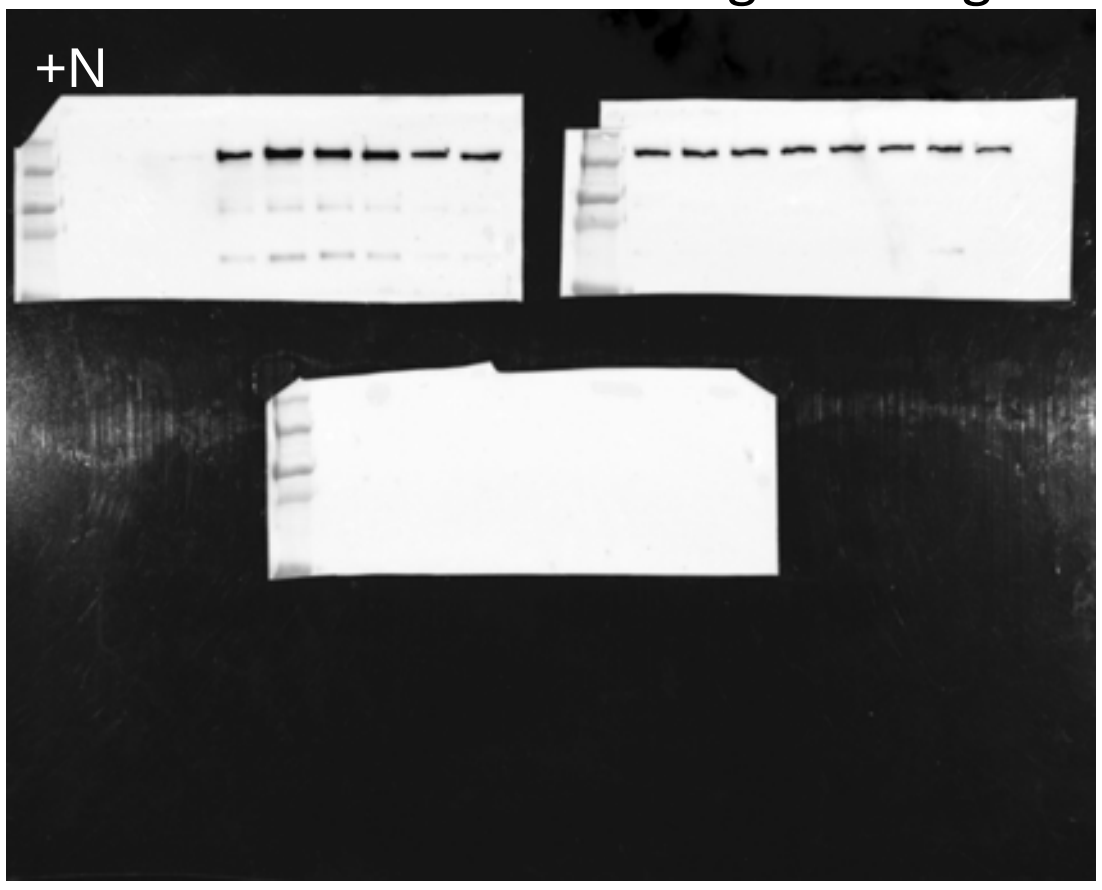

Used areas

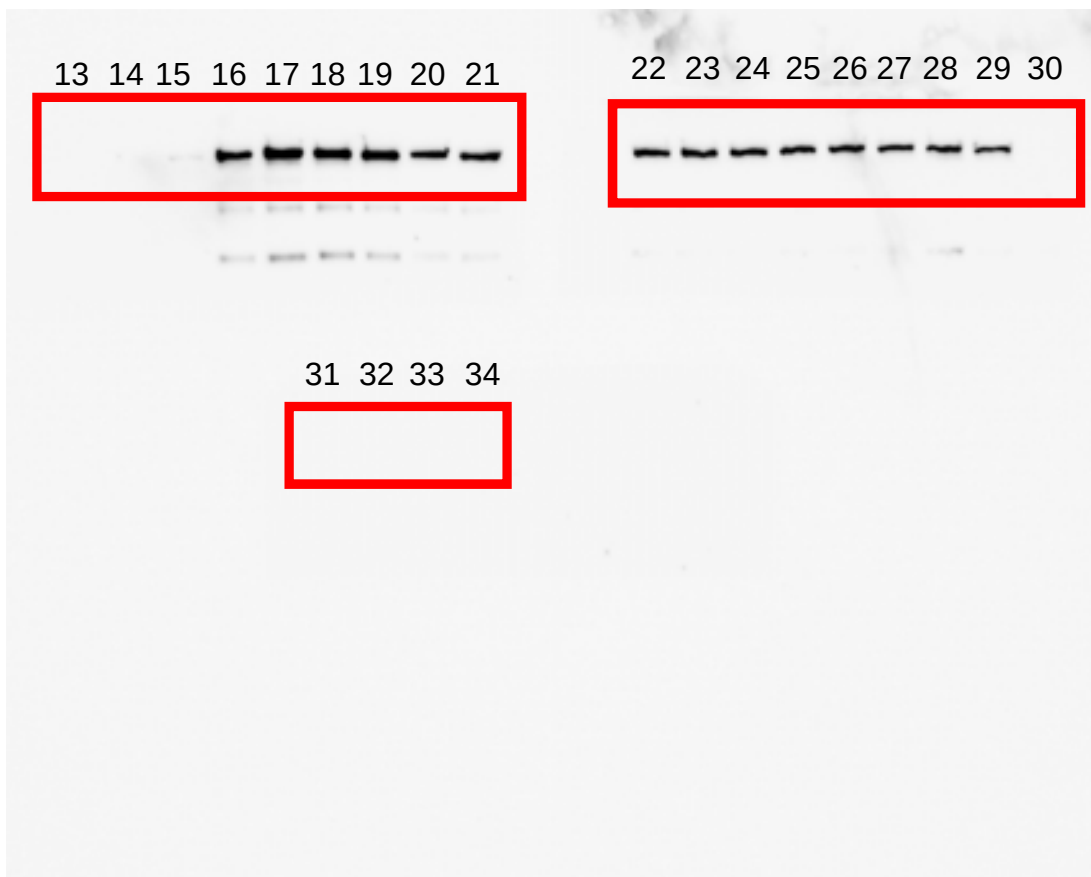

α-HA TRAPPC11 *trs85*Δ +N original images

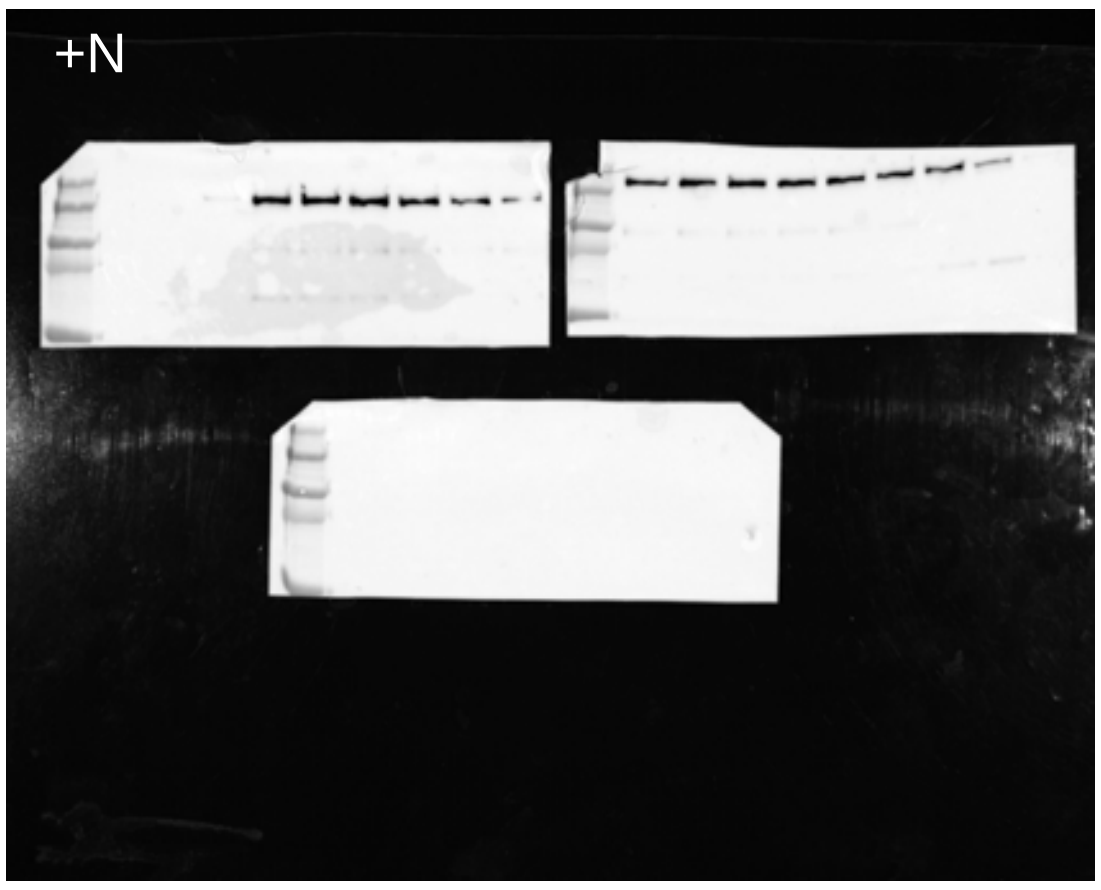

Used areas

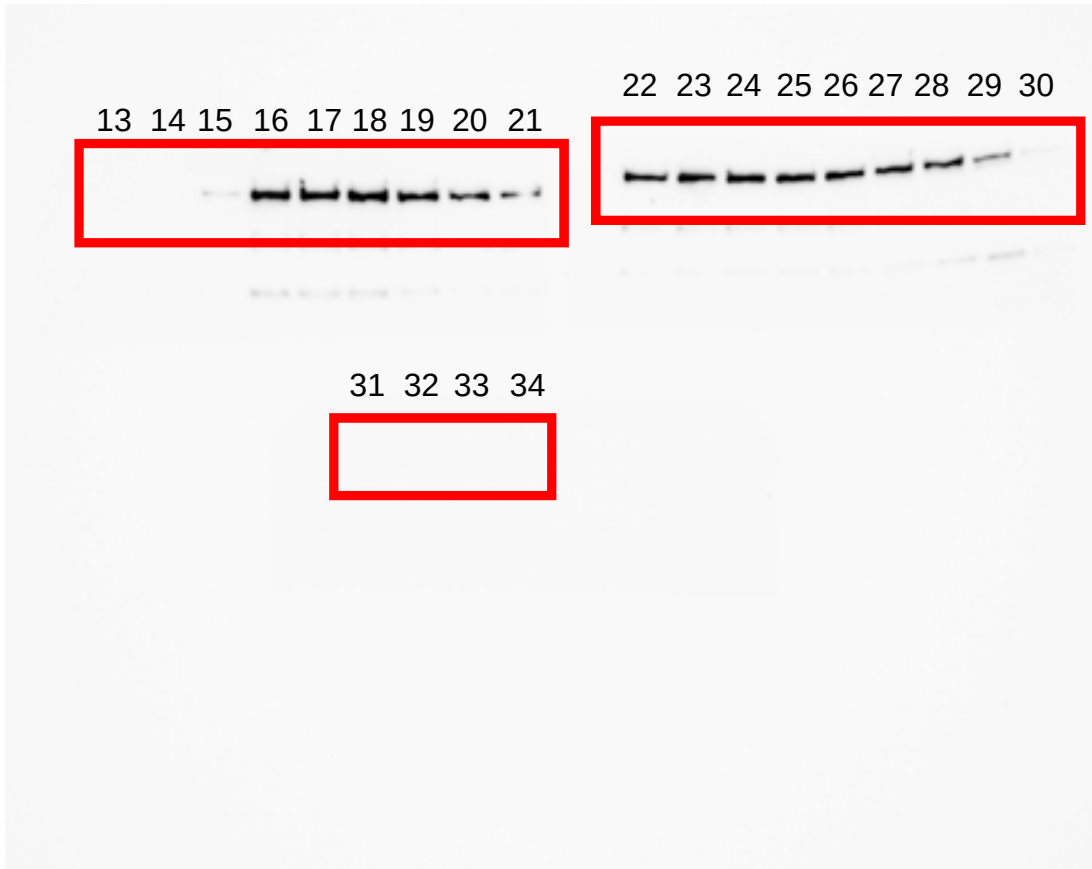

Figure 6H

α-HA TRAPPC11 wt -N original images

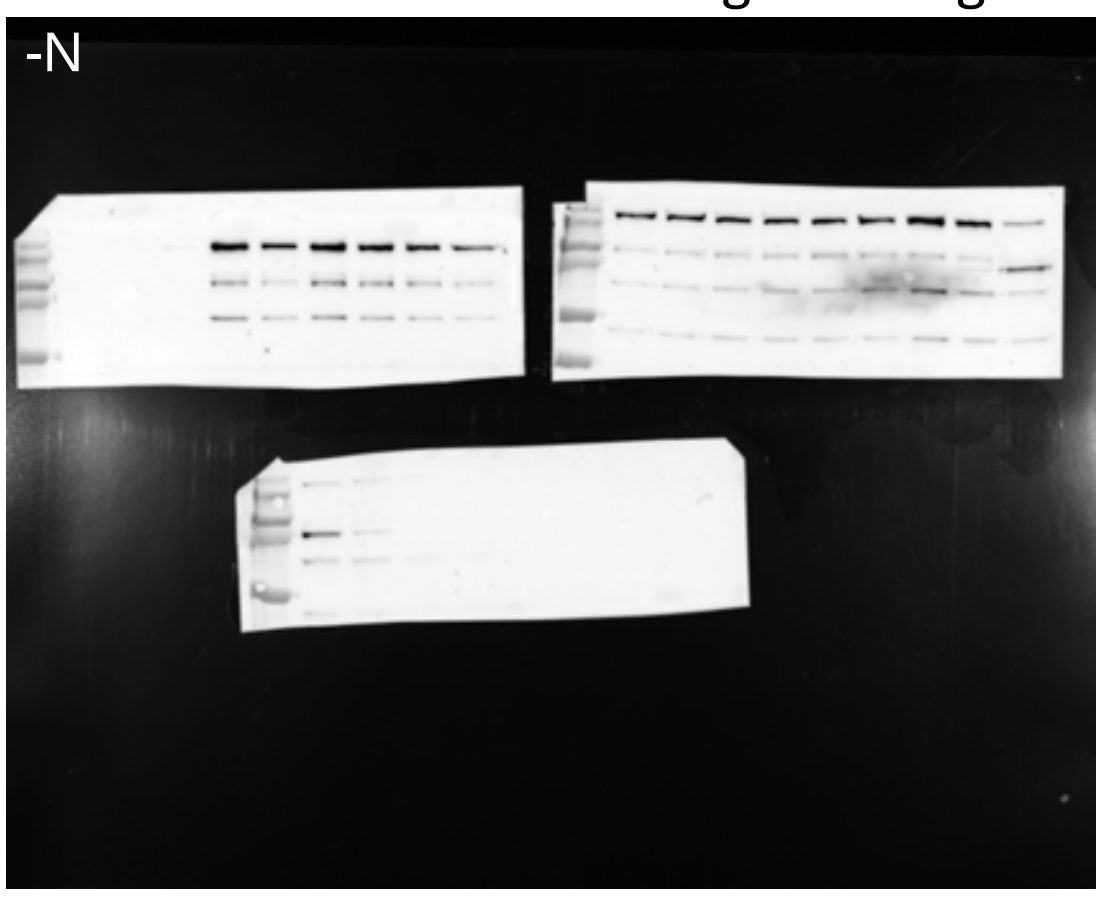

Used areas

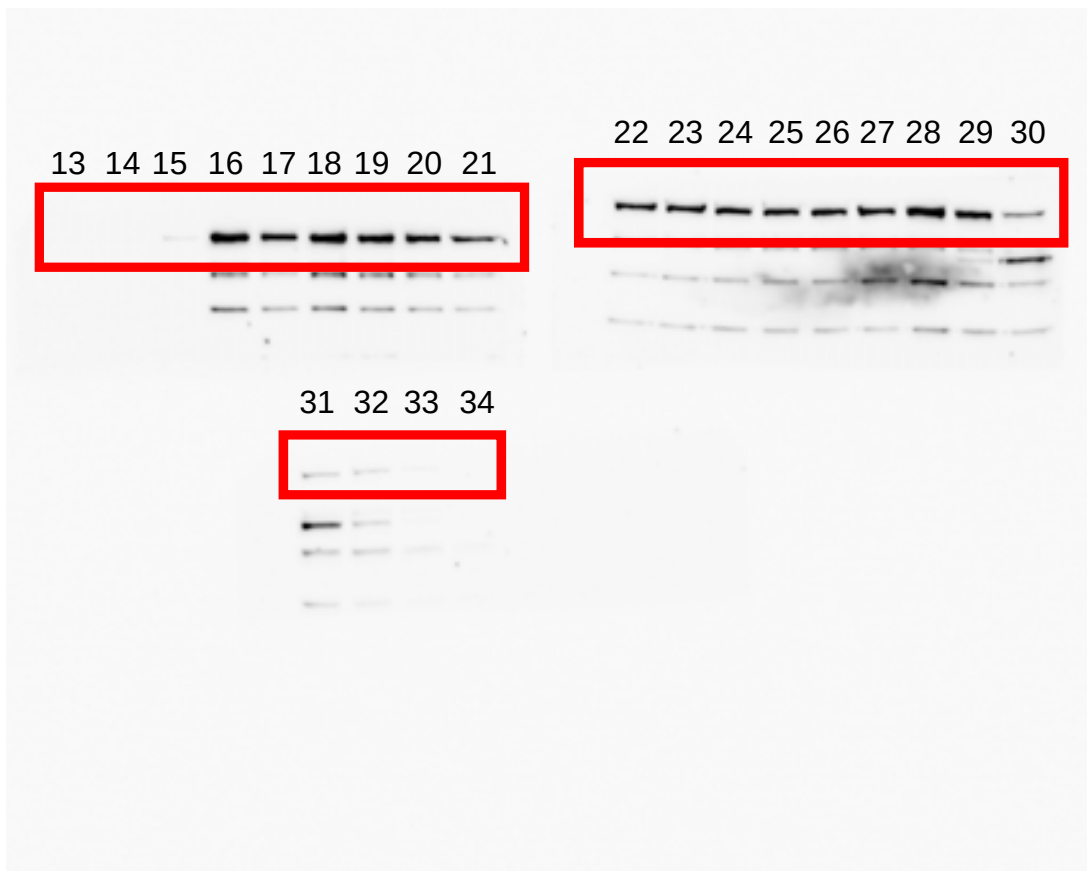

α-HA TRAPPC11 *trs85*Δ -N original images

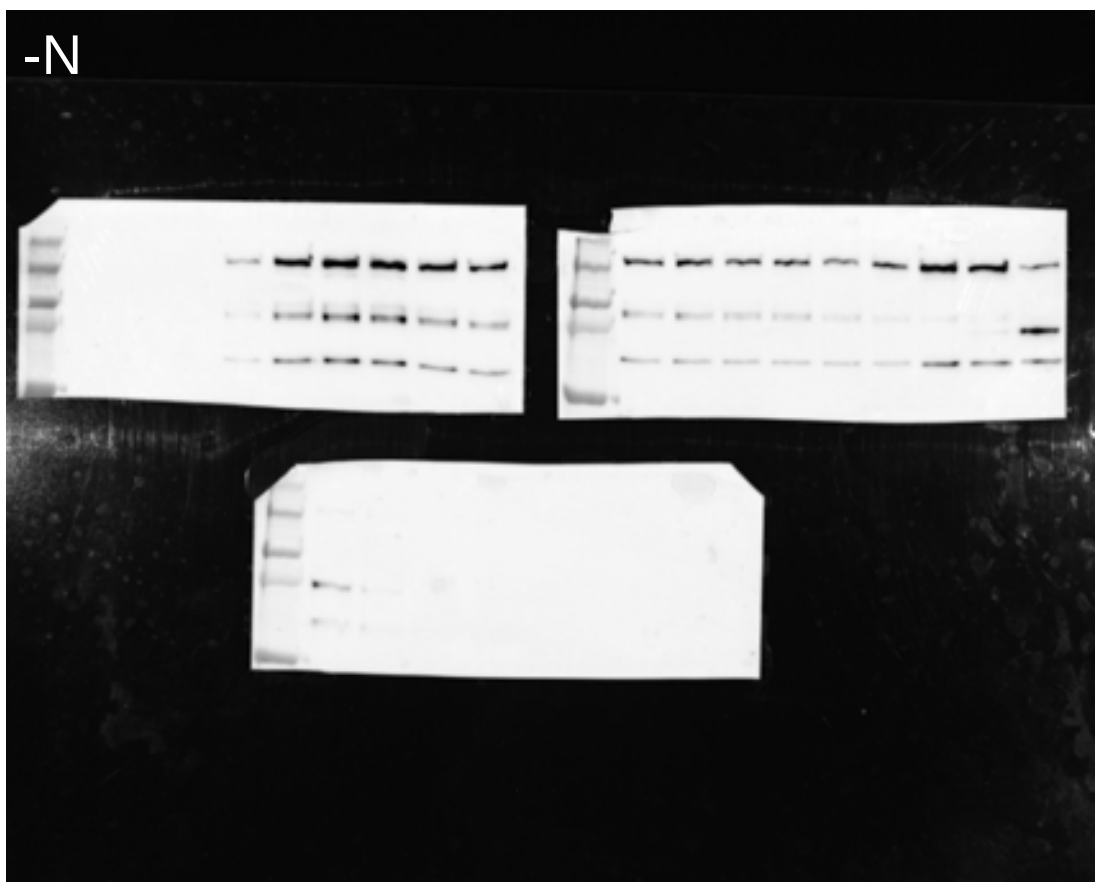

Used areas

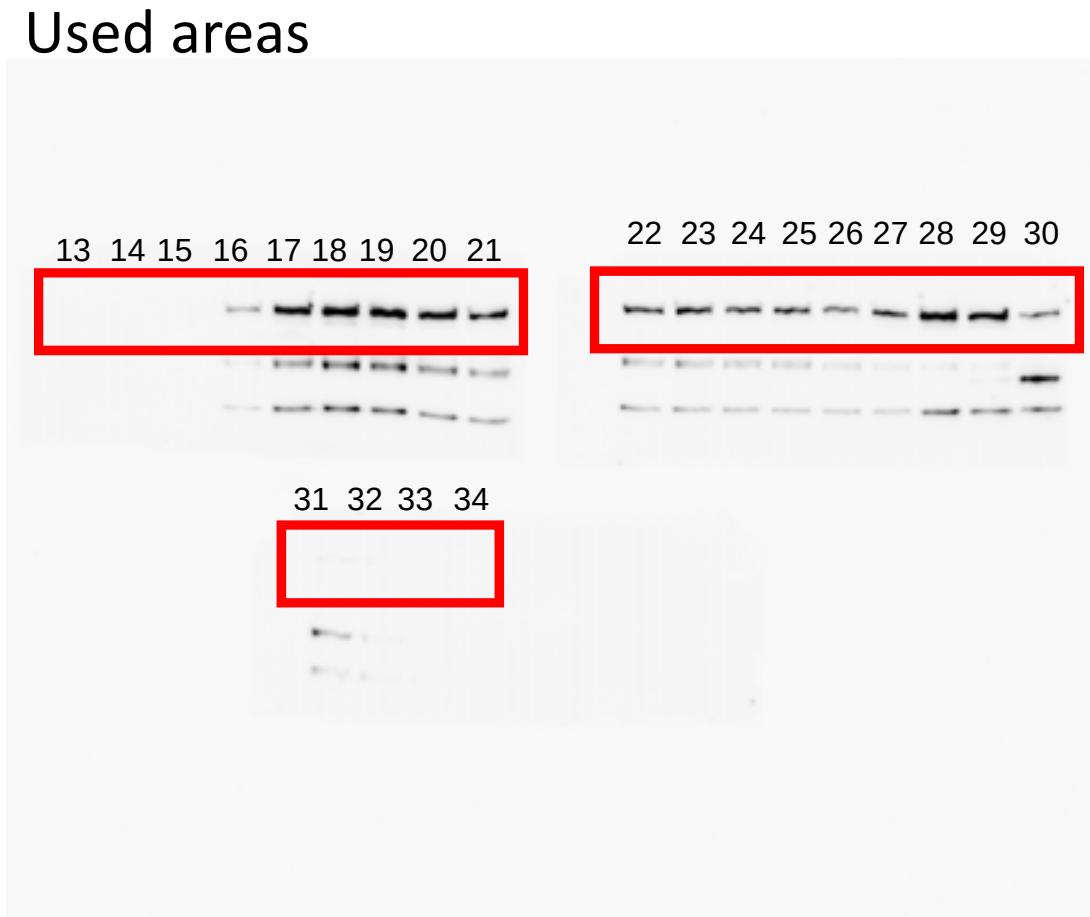

Fig. S5. Blot transparency.

Journal of Cell Science • Supplementary information

Table S1. Strains used in this work

Strain code	Genotipe
MAD005	wild type
MAD3807	pabaA1; pyroA4::[pyroA gpdam::gfp::atg8] nkuAΔ::bar?
MAD4401	yA2; pyroA4::[pyroA gpdam::mcherry::atg8]; pantoB100
MAD5971	trs33Δ::riboB^{Af}; pyroA4::[pyroA gpdam::gfp::atg8] nkuAΔ::bar?; riboB2?
MAD6457	trs33Δ::pyrG^{Af} ; trs85Δ::pyroA^{Af} pyroA4::[pyroA gpdam::gfp::atg8] nkuAΔ::bar?; pantoB100; riboB2
MAD6579	pabaA1 pyrG89; bet5::stag::pyrG^{Af}; nkuAΔ::bar
MAD6855	pyrG89; trappc12::gfp::pyrG^{Af}; pyroA4
MAD6859	pyrG89; pyroA4 nkuAΔ::bar; trappc11::Stag::pyrG^{Af}
MAD6946	pyrG89?; pyroA4::[pyroA gpdam::gfp::atg8] nkuAΔ::bar; trappc11Δ::pyrg^{Af}
MAD6949	pyrG89?; trappc12Δ::pyrg^{Af}; pyroA4::[pyroA gpdam::gfp::atg8] nkuAΔ::bar
MAD7067	pabaA1 pyrG89; trs85::3xgfp::pyrG^{Af} nkuAΔ::bar
MAD8311	pyrG89?; trs85Δ::pyrg^{Af} pyroA4::[pyroA gpdam::gfp::atg8] nkuAΔ::bar?; trappc11Δ::pyrg^{Af}
MAD8313	pyrG89?; trs85Δ::pyrg^{Af} pyroA4::[pyroA gpdam::gfp::atg8] nkuAΔ::bar?
MAD8338	pyrG89? trs33Δ::riboB^{Af};pyroA4::[pyroA gpdam::gfp::atg8] nkuAΔ::bar?; trappc11Δ::pyrg^{Af}
MAD8439	tca17Δ::pyrg^{Af}; pyroA4::[pyroA gpdam::gfp::atg8] nkuAΔ::bar?; rab11^{D125E}
MAD8441	tca17Δ::pyrg^{Af}; trs85Δ::pyrG^{Af} pyroA4::[pyroA gpdam::gfp::atg8] nkuAΔ::bar?; rab11^{D125E}
MAD8604	pyrG89; pyroA4 nkuAΔ::bar; trappc11::3xgfp::pyrG^{Af}
MAD8900	pyrG89?; pyroA4::[pyroA gpdam:: mcherry::atg8] nkuAΔ::bar?; trappc11::3xgfp::pyrG^{Af}
MAD8935	pyrG89; pyroA4 nkuAΔ::bar; trappc11::ha_3::pyrG^{Af}
MAD9005	pyrG89? trs33Δ::riboB^{Af}; pyroA4 nkuAΔ::bar?; pantoB100; trappc11::ha_3::pyrG^{Af}
MAD9026	pyrG89?; trs23::ha_3::pyrG^{Af} pyroA4 nkuAΔ::bar?; trappc11::ha_3::pyrG^{Af}
MAD9033	pyrG89; trs85::ha_3::pyrG^{Af} pyroA4 nkuAΔ::bar; trappc11::ha_3::pyrG^{Af}
MAD9314	trs33Δ::riboB^{Af};pyroA4::[pyroA gpdam:: mcherry::atg8] nkuAΔ::bar?; trappc11::3xgfp::pyrg^{Af}
MAD9332	pyrG89; trs85Δ::pyrG^{Af} pyroA4 nkuAΔ::bar; trappc11::ha_3::pyrG^{Af}
MAD9336	trs85Δ::pyrg^{Af} pyroA4::[pyroA gpdam:: mcherry::atg8] nkuAΔ::bar?; trappc11::3xgfp::pyrG^{Af}
MAD9461	pabaA1; trs85::3xgfp::pyrG^{Af} pyroA4::[pyroA gpdam::mcherry ::atg8] nkuAΔ::bar?; pantoB100
MAD9462	pyrG89? trs33Δ::riboB^{Af}; trs85::3xgfp::pyrg^{Af} pyroA4::[pyroA gpdam::mcherry::atg8] nkuAΔ::bar?;
MAD9725	pabaA1; trs85::3Xgfp::pyrg^{Af} pyroA4::[pyroA gpdam:: mcherry::atg8] nkuAΔ::bar?; trappc11Δ::pyrG^{Af}
MAD9726	pyrG89; bet5-stag::pyrG^{Af}; pyroA4 nkuAΔ::bar; trappc11Δ::pyrg^{Af}
MAD9752	pabaA1 pyrG89?; trappc12Δ::pyrg^{Af}; trs85Δ::pyrG^{Af} pyroA4::[pyroA gpdam::gfp::atg8] nkuAΔ::bar?
MAD9754	pyrG89; trappc13Δ::pyrg^{Af}; trs85Δ::pyrG^{Af} pyroA4::[pyroA gpdam::gfp::atg8] nkuAΔ::bar?
MAD9755	pyrG89; trappc13Δ::pyrg^{Af}; pyroA4::[pyroA gpdam::gfp::atg8] nkuAΔ::bar?
MAD9772	pyrG89?; trappc12Δ::pyrg^{Af}; trs85::3xgfp::pyrG^{Af} pyroA4::[pyroA gpdam::mcherry::atg8] nkuAΔ::bar?
MAD9841	pyrG89? trs33Δ::riboB^{Af}; trappc12::gfp::pyrg^{Af} ; pyroA4::[pyroA gpdam::mcherry::atg8]; pantoB100
MAD9843	trappc12::gfp::pyrG^{Af}; pyroA4::[pyroA gpdam::mcherry::atg8]; pantoB100; trappC11Δ::pyrG^{Af}
MAD9845	trappc12::gfp::pyrG^{Af}; pyroA4::[pyroA gpdam::mcherry::atg8]; pantoB100
MAD9847	wA3; tca17Δ::pyrG^{Af}; pyroA4::[pyroA gpdam::mcherry::atg8]; rab11^{D125E} trappc11::3xgfp::pyrG^{Af}
MAD9850	wA3; tca17Δ::pyrG^{Af}; pyroA4::[pyroA gpdam::mcherry::atg8] trs85::3xgfp::pyrG^{Af}; rab11^{D125E}
MAD9852	pyrG89?; trappc12Δ::pyrg^{Af}; pyroA4::[pyroA gpdam::mcherry::atg8]; trappc11::3xgfp::pyrG^{Af}

Journal of Cell Science • Supplementary information

Table S2. Oligonucleotides used for genetic manipulation.

**Primer name**	**Sequence 5´-3´**
***trappc11* Knock- Out cassette**	
Fw *trappc11*p	CCTTTGGTGAAGTGAGCTGCTA
Rev *trappc11*p	CGGACCTGAAGTGCTGTTGAC
Fw join 5UTR *trappc11-pyrG^{Af}*	GCCGGCTGTCAACAGCACTTCAGGTCCGGTAACGGCCGCCAGTGTGCTG
Rv join 3UTR *trappc11-pyrG^{Af}*	GCAGAGTCCTGCTAGCCATTATGAATGGTCTGAGAGGAGGCACTGATG
Fw *trappc11*t	CATTCATAATGGCTAGCAGGACTC
Rv *trappc11*t	GGATCATCTGGTCCCGAAACTG
***trappc12* Knock- Out cassette**	
Fw *trappc12*p	CACAGGATGATGCACAGCACAC
Rev *trappc12*p	GTGGATTACGCCTCTTACTC
Fw join 5UTR *trappc12-pyrG^{Af}*	GCCAGACGAGTAAGAGGCGTAATCCACGATTTCAGTAACGTTAAGTGG
Rv join 3UTR *trappc12-pyrG^{Af}*	ATGCCACCCTGCACCTTCGTTTGAGTAGGACAGAAGATGATATTGAAGG
Fw *trappc12*t	CTACTCAAACGAAGGTGCAG
Rv *trappc12*t	GCCAATCTCATTCTCCCAGCG
***trappc13* Knock- Out cassette**	
Fw *trappc13*p	GGTTGCCTTTCCTTGCATCCG
Rev *trappc13*p	GAACTTCGAGTTGCTTCAAGCTG
Fw join 5UTR *trappc13-pyrG^{Af}*	GCTTCCCAGCTTGAAGCAACTCGAAGTTCGTAACGGCCGCCAGTGTGCTG
Rv join 3UTR *trappc13-pyrG^{Af}*	GCCTCGCTGTGCTATGTTATAAGAACCGTCTGAGAGGAGGCACTGATG
Fw *trappc13*t	GGTTCTTATAACATAGCACAGCG
Rv *trappc13*t	GTAGAAGCTCGTCTTCGACATCG
***trappc11*::3xgfp cassette**	
Fw *trappc11* ORF	CCATAATTCGGCCATTCGAGGC
Rv *trappc11* ORF join GAs GFPs	CCGGCTCCAGCGCCTGCACCAGCTCCATCCTCAGCATCGACCCATATC
Fw GAs GFPs join *trappc11*	CCTGATATGGGTCGATGCTGAGGATGGAGCTGGTGCAGGCGCTGGAG
Rv GFP join PyrGaf	AGAGGGTGAAGAGCATTGTTTGAGGCTCACTACTTGTAGAGCTCGTC
Fw PyrGAf	GCCTCAAACAATGCTCTTCACC
Rv PyrGAf join 3 UTR *trappc11*	CAGAGTCCTGCTAGCCATTATGAATGGTCTGAGAGGAGGCACTGATGC
Fw 3 UTR *trappc11*	CATTCATAATGGCTAGCAGGACTC
Rv 3 UTR *trappc11*	GGATCATCTGGTCCCGAAACTG
***trappc12*::gfp cassette**	
Fw ORF *trappc12*	GAACATCGCAGCTCAAGAATCG
Rev ORF *trappc12*	CAGTTTGAAATCAGCGTTCGGC
Fw join ORF *trappc12 GFP-PyrG^{Af}*	GATCGGCCGAACGCTGATTTCAAACTGGGAGCTGGTGCAGGCGCTGGAG
Rv join 3UTR *trappc12 -pyrG^{Af}*	GCACCTTCGTTTGAGTAGCTATCTTATGTCTGAGAGGAGGCACTGATG
Fw 3UTR *trappc12*	ATAAGATAGCTACTCAAACGAAGGTG
Rv 3UTR *trappc12*	GCCAATCTCATTCTCCCAGCG
***trappc11*::stag cassette**	
Fw ORF *trappc11*	CCATAATTCGGCCATTCGAGGC

Journal of Cell Science • Supplementary information

Rev ORF *trappc11*	ATCCTCAGCATCGACCCATATC
Fw join ORF *trappc11 stag-PyrG^{Af}*	ATCCTGATATGGGTCGATGCTGAGGATGGAGCTGGTGCAGGCGCTGGAG
Rv join 3UTR *trappc11-pyrG^{Af}*	GCAGAGTCCTGCTAGCCATTATGAATGGTCTGAGAGGAGGCACTGATG
Fw 3UTR *trappc11*	CATTCATAATGGCTAGCAGGACTC
Rv 3UTR *trappc11*	GGATCATCTGGTCCCGAAACTG

***trappc11*::HA cassette**

Fw ORF *trappc11*	CCATAATTCGGCCATTCGAGGC
Rev ORF *trappc11*	ATCCTCAGCATCGACCCATATC
Fw join ORF *trappc11 HA-PyrG^{Af}*	ATCCTGATATGGGTCGATGCTGAGGATGGAGCTGGTGCAGGCGCTGGAG
Rv join 3UTR *trappc11-pyrG^{Af}*	GCAGAGTCCTGCTAGCCATTATGAATGGTCTGAGAGGAGGCACTGATG
Fw 3UTR *trappc11*	CATTCATAATGGCTAGCAGGACTC
Rv 3UTR *trappc11*	GGATCATCTGGTCCCGAAACTG

Journal of Cell Science • Supplementary information
