# Supplementary material for: A subcomplex comprising TRAPPC11, TRAPPC12, TRAPPC13 and the fungal TRAPPC2L homolog, Tca17, directs TRAPPIII to autophagy
Source: J Cell Sci. 2026 Aug 27;139(16):jcs264822. doi: 10.1242/jcs.264822 (PMC13571812; doi:10.1242/jcs.264822)
Supplement: Supplementary file 2 [file joces-139-264822_review_history.pdf]

Journal of Cell Science

A subcomplex comprising TRAPPC11, TRAPPC12, TRAPPC13 and the fungal TRAPPC2L homolog, Tca17, directs TRAPPIII to autophagy

Mario Pinar, Vivian de los Rıos, Silvia Rodríguez-Pires, Eduardo A. Espeso and Miguel A. Peñalva

DOI: 10.1242/jcs.264822

Editor: Tamotsu Yoshimori

Review timeline

Original submission:	24 February 2026
Editorial decision:	23 March 2026
First revision received:	14 May 2026
Editorial decision:	9 June 2026
Second revision received:	11 June 2026
Accepted:	18 June 2026

Original submission

First decision letter

MS ID#: jcs.264822

MS TITLE: A TRAPPC2L/TRAPPC11/12/13 subcomplex directs TRAPPIII to autophagy

AUTHORS: Mario Pinar; Vivian De los Rios; Silvia Rodríguez-Pires; Eduardo A. Espeso; Miguel A Penalva
ARTICLE TYPE: Research Article

Dear Dr Penalva,

We have now reached a decision on the above manuscript.

As you will see, both reviewers are generally positive about your study. Reviewer #1 has suggested minor revisions, while Reviewer #2 has requested two additional experiments to further strengthen the manuscript.

If you are able to address these points satisfactorily, I would be pleased to see a revised manuscript. We would then return it to the reviewers. for further evaluation.

Reviewer 1

In this manuscript from Pinar and colleagues, the authors investigate the composition and function of the TRAPPIII complex, a GEF for Rab1, using A. nidulans, which is an excellent model for studying exocytic trafficking. The new insights in this paper focus on differences in TRAPP composition during nitrogen starvation (autophagy), compared to normal growth.

The authors begin with the observation that the two non-essential components of the TRAPPIII complex, Trs85 and Trs33, exhibit a strong synthetic genetic interaction for growth, and also for autophagy. Trs33 is known to link the TRAPPC11/12/13 subunits to the TRAPP core in TRAPPIII, and to link the Trs130 subunit to the core in TRAPPII.

© 2026. Published by The Company of Biologists under the terms of the Creative Commons Attribution License (https://creativecommons.org/licenses/by/4.0/).

The authors therefore investigated the impact of TRAPPC11/12/13 knockouts, and found no impact on growth or autophagy for individual single knockouts. In contrast, combining C11 knockout with Trs85 knockout had significant impacts on both growth and autophagy.

The authors then examine the localization of C11-GFP and Trs85-GFP to the PAS in cells, and find that Trs85-GFP localization is reduced by ~70% (from 85% to 26%) in TRAPPC11 knockouts, and C11-GFP localization is reduced by ~55% (from 91% to 41%) in Trs85 knockouts. This suggests both C11 and Trs85 cooperate to localize the complex.

The authors then find that upon nitrogen starvation, the amount of TRAPPC11/C12/C13, which is was previously found by this lab to be substoichiometric in the TRAPPIII complex, increases roughly 2-fold. They also observe that under these conditions, a significant fraction of C11 has been incorporated into the TRAPPIII complex. The authors refer to the two versions of the complex as TRAPPIIIa (without C11/C12/C13) and TRAPPIIIb (with C11/C12/C13).

Taken together, the results indicate that Aspergillus contains both a metazoan-like TRAPPIII complex (TRAPPIIIb), and a S. cerevisiae-like TRAPPIII complex (TRAPPIIIb). As nitrogen starvation induces the formation of more TRAPPIIIb, and the C11/C12/C13 subunits are important for autophagy, the TRAPPIIIb complex appears to be specialized for autophagy.

Overall I found this to be a nice study that clarifies the composition and function of the TRAPP complexes in A. nidulans during nitrogen starvation. A. nidulans is an important model that can be considered in this case to bridge metazoans and budding yeast, and therefore sheds light on mechanisms in both systems, and the TRAPP complexes more broadly.

Suggestions for improvement:

The nomenclature sometimes is a bit confusing. For example, the authors use "TCA17/TRAPPC2L" to refer to a single subunit that has two different names. But they use "TRAPPC11/C12/C13" to refer to three different subunits. So it is easy for the reader to think that "TCA17/TRAPPC2L" refers to two subunits instead of one.

I was a little confused by the focus on TCA17/TRAPPC2L. The abstract made me think that some new function of TCA17/TRAPPC2L was being uncovered. But this protein was already known to be required to link C11/C12/C13 to the core complex.

Reviewer 2

The TRAPP complexes are key regulators of intracellular membrane trafficking. TRAPPIII functions as a guanine nucleotide exchange factor (GEF) for RAB1 during autophagosome formation, whereas TRAPPII, despite sharing the same core, acts at the trans-Golgi network through RAB11 activation. In budding yeast, the TRAPPIII-specific subunit Trs85 has been shown to be required for targeting the complex to sites of autophagosome formation. By contrast, in metazoans and plants, additional subunits such as TRAPPC11, TRAPPC12, and TRAPPC13 are present, but how these components contribute to the organization and function of TRAPPIII remains incompletely understood. In this study, using the ascomycete Aspergillus nidulans, the authors demonstrate that TRAPPC11/12/13 interact with the TRAPP GEF core via Tca17 (TRAPPC2L in mammals) and act together with Trs85 to direct TRAPPIII to sites of autophagosome formation. They further propose the existence of two forms of the TRAPPIII complex, one lacking and the other containing TRAPPC11/12/13, which they designate TRAPPIIIa and TRAPPIIIb, respectively. Overall, the conclusions are generally supported by the data presented. However, the manuscript would benefit from further strengthening of the experimental support, as described below..

Major comments

1. Figure 2: The reproducibility and statistical significance of the data should be demonstrated by repeating the experiments. Quantification of Atg8 puncta per cell should also be included. Including the Tca17-delta strain would further strengthen the analysis. In addition, it should be

© 2026. Published by The Company of Biologists under the terms of the Creative Commons Attribution License (https://creativecommons.org/licenses/by/4.0/).

examined whether deletion of TRS33 or TRAPPC11 affects the localization of TRAPPC12 to Atg8 puncta.

2. Figure S1: The colocalization between TRAPPC12 and Atg8 should also be presented.

First revision

Author response to reviewers' comments

We thank the referees for their thoughtful comments. Below follows a detailed description on how we have addressed their requests.

Referee 1

The nomenclature sometimes is a bit confusing. For example, the authors use "TCA17/TRAPPC2L" to refer to a single subunit that has two different names. But they use "TRAPPC11/C12/C13" to refer to three different subunits. So, it is easy for the reader to think that "TCA17/TRAPPC2L" refers to two subunits instead of one.

The referee is right in that this nomenclature was very confusing. We have removed the designation Tca17/TRAPPC2L and denoted Aspergillus TRAPPC2L as Tca17 all across the manuscript, excepting in the title and the abstract, where we have added TRAPPC2L as superscript after Tca17 to remind readers that these proteins are functional homologues (we think that this might help catching attention of potential readers). Therefore, protein names separated by slashes are solely used for TRAPPC11/12/13. In addition, we have added, in places where we thought it would be helpful, a parenthetical indication of the name of the Metazoan homologue of the fungal protein of interest.

I was a little confused by the focus on TCA17/TRAPPC2L. The abstract made me think that some new function of TCA17/TRAPPC2L was being uncovered. But this protein was already known to be required to link C11/C12/C13 to the core complex.

The referee is right in that Tca17 was known to be required to link TRAPPC11/12/13 to the core. The novel, and in our view important, finding is that these four proteins can be isolated as a stable subcomplex from cells and therefore determine the fate of the core complex, targeting it to autophagy in a similar manner than addition of the stable Tca17/Trs120/Trs130/Trs65 subcomplex that we described previously targets the core complex to the secretory pathway. Therefore, our data suggest a Lego-like customization of TRAPPs using preassembled core and at least two different stable subcomplexes in which specificity for membranes resides.

Figure 2: The reproducibility and statistical significance of the data should be demonstrated by repeating the experiments. Quantification of Atg8 puncta per cell should also be included.

Aspergillus nidulans hyphal tip cells are 125 µm long on average, making it very difficult to image whole cells in the same focal plane across their entire length. Considering this limitation, the actual counting of PASs per cell is not informative, as there is a marked variation from cell to cell. Nevertheless, to try to address the request made by the referee, we have added in the corresponding figure legends, for each of the two tables reporting quantitative data in this revised version (Figure 2R panel D and Figure 3R, panel A), the number of cells from which the total counts of Atg8 PASs were derived, such that readers can obtain an approximation to the figures requested by the referee.

Including the Tca17-delta strain would further strengthen the analysis.

Included as requested. *tca17Δ* completely delocalizes TRAPPC11 from the PAS (R-Figure 2F), but does not affect Trs85 located on the opposite side of the complex (R-Figure 3D).

© 2026. Published by The Company of Biologists under the terms of the Creative Commons Attribution License (https://creativecommons.org/licenses/by/4.0/).

Figure S1: The colocalization between TRAPPC12 and Atg8 should also be presented.
Thank you for the suggestion. We have included, in addition to the requested data on the co-localization of Atg8-PASs and TRAPPC12, new data on the localization of TRAPPC12 in *trappc11Δ* (R-Figure 2G) and *trs33Δ* (R-Figure 2E) backgrounds, showing that TRAPPC12 behaves like TRAPPC11. We have additionally demonstrated that, in full agreement with our conclusions, *trappc12Δ* does not delocalize TRAPPC11 from the Atg8 PASs (R-Figure 2G, right panel), nor does it delocalize Trs85 located on the opposite side of the complex (R-Figure 3D).

Second decision letter

MS ID#: jcs.264822R1

MS TITLE: A Tca17TRAPPC2L/TRAPPC11/12/13 subcomplex directs TRAPPIII to autophagy

AUTHORS: Mario Pinar; Vivian De los Rios; Silvia Rodríguez-Pires; Eduardo A. Espeso; Miguel A Penalva
ARTICLE TYPE: Research Article

Dear Dr Penalva,

Thank you for submitting your revised manuscript.

Reviewer 1 is satisfied with the revision. However, Reviewer 2 raises a remaining concern regarding the quantitative analyses in Figs. 2D and 3A. Specifically, it is still unclear how many independent biological experiments were performed and whether the results were reproducibly observed across these experiments.

Please address this point and revise the manuscript accordingly.

Reviewer 1

The authors have done a nice job of addressing the points raised during the first round of review. I recommend publication!

Reviewer 2

While the authors explain the technical limitations associated with counting PASs on a per-cell basis and have now provided the number of hyphae analyzed in the legends to Figs. 2D and 3A, it remains unclear how many independent biological experiments were performed and whether the reported results were consistently observed across these experiments. This information should be clarified.

Second revision

Author response to reviewers' comments

Reviewer 2: While the authors explain the technical limitations associated with counting PASs on a per-cell basis and have now provided the number of hyphae analyzed in the legends to Figs. 2D and 3A, it remains unclear how many independent biological experiments were performed and whether the reported results were consistently observed across these experiments. This information should be clarified.

© 2026. Published by The Company of Biologists under the terms of the Creative Commons Attribution License (https://creativecommons.org/licenses/by/4.0/).

Thank you for your suggestion. To address your concerns, we have now included the following subheading in the Materials and Methods.

Reproducibility of quantitative colocalization data in Figures 2D and 3A.

All results were reproducible across experiments. For all colocalization experiments involving combinations of TRAPPC11-GFP, TRAPPC12-GFP, and Trs85-GFP with mCherry-Atg8 in the wild type and various mutant backgrounds, eight independent gene-replaced clones were screened by fluorescence microscopy to confirm the reproducibility of the observed phenotypes, irrespective of whether they were obtained by transformation or by genetic crossing. Quantitative data were obtained from two biological replicates, except for the TRAPPC11-GFP and Trs85-GFP colocalization experiments with the mCherry-Atg8-labeled PAS in the wild-type background, for which four biological replicates were analyzed. For those mutants in which the GFP reporter was not detectable in the PAS, the presence of the transgene was verified by diagnostic PCR.

Third decision letter

MS ID#: jcs.264822R2

MS Title: A Tca17TRAPPC2L/TRAPPC11/12/13 subcomplex directs TRAPPIII to autophagy

Authors: Mario Pinar; Vivian De los Rios; Silvia Rodríguez-Pires; Eduardo A. Espeso; Miguel A Penalva

Article Type: Research Article

Dear Dr Penalva,

I am happy to tell you that your manuscript has been accepted for publication in Journal of Cell Science, pending standard publication integrity checks.

© 2026. Published by The Company of Biologists under the terms of the Creative Commons Attribution License (https://creativecommons.org/licenses/by/4.0/).
